# Is sibship composition a risk factor for childhood asthma? Systematic review and meta-analysis

**DOI:** 10.1007/s12519-023-00706-w

**Published:** 2023-03-30

**Authors:** Daniil Lisik, Saliha Selin Özuygur Ermis, Athina Ioannidou, Gregorio Paolo Milani, Sungkutu Nyassi, Giulia Carla Immacolata Spolidoro, Hannu Kankaanranta, Emma Goksör, Göran Wennergren, Bright Ibeabughichi Nwaru

**Affiliations:** 1 Krefting Research Centre, Department of Internal Medicine and Clinical Nutrition, Institute of Medicine, University of Gothenburg, Medicinaregatan 1F, 413 90 Göteborg, Sweden; 2 Department of Respiratory Medicine, Dokuz Eylul University, Izmir, Turkey; 3 Department of Clinical Science and Community Health, University of Milan, 20122 Milan, Italy; 4 Pediatric Unit, Fondazione IRCCS Ca’ Granda Ospedale Maggiore Policlinico, 20122 Milan, Italy; 5 Tampere University Respiratory Research Group, Faculty of Medicine and Health Technology, Tampere University, Tampere, Finland; 6 Department of Respiratory Medicine, Seinäjoki Central Hospital, Seinäjoki, Finland; 7 Department of Pediatrics, Sahlgrenska Academy at University of Gothenburg, Gothenburg, Sweden; 8 Wallenberg Centre for Molecular and Translational Medicine, University of Gothenburg, Gothenburg, Sweden

**Keywords:** Asthma, Hygiene hypothesis, Respiratory sounds, Siblings, Systematic review

## Abstract

**Background:**

Following the “hygiene hypothesis”, the role of sibship composition in asthma and wheezing has been extensively studied, but the findings are inconsistent. For the first time, this systematic review and meta-analysis synthesized evidences from studies investigating the association of sibship size and birth order with risk of asthma and wheezing.

**Methods:**

Fifteen databases were searched to identify eligible studies. Study selection and data extraction were performed independently by pairs of reviewers. Meta-analysis with robust variance estimation (RVE) was used to produce pooled risk ratio (RR) effect estimates from comparable numerical data.

**Results:**

From 17,466 identified records, 158 reports of 134 studies (> 3 million subjects) were included. Any wheezing in the last ≤ 1.5 years occurred more frequently in infants with ≥ 1 sibling [pooled RR 1.10, 95% confidence interval (CI) 1.02–1.19] and ≥ 1 older sibling (pooled RR 1.16, 95% CI 1.04–1.29). The pooled effect sizes for asthma were overall statistically nonsignificant, although having ≥ 1 older sibling was marginally protective for subjects aged ≥ 6 years (pooled RR 0.93, 95% CI 0.88–0.99). The effect estimates weakened in studies published after 2000 compared with earlier studies.

**Conclusions:**

Being second-born or later and having at least one sibling is associated with a slightly increased risk of temporary wheezing in infancy. In contrast, being second-born or later is associated with marginal protection against asthma. These associations appear to have weakened since the turn of the millennium, possibly due to lifestyle changes and socioeconomic development.

**Video Abstract**

**Supplementary Information:**

The online version contains supplementary material available at 10.1007/s12519-023-00706-w.

## Introduction

Asthma is a heterogeneous chronic inflammatory pulmonary disease [1–3] characterized by usually reversible [4] expiratory airflow limitation and wheezing, dyspnea, cough, and chest tightness [2, 5]. Following a sharp increase in prevalence—particularly in high-income countries [3, 6]—in the second half of the twentieth century [7], hundreds of millions are affected worldwide [8]; however, recent trends are mixed, with reports of levelling-off/decrease in some regions and increase in others [9–11]. Numerous studies [3, 12, 13] have been published aiming to elucidate risk factors responsible for the heterogeneous distribution and clinical burden of asthma [14]. Susceptibility to developing asthma may be partially established in utero [15]; therefore, some studies focus on identifying prenatal and early life environmental risk factors for asthma, such as sibship composition, which gained widespread attention after Strachan found an inverse correlation between birth order and atopic diseases such as allergic rhinitis [16]. This association is commonly attributed to the “hygiene hypothesis”, which suggests that cross-infection between siblings, particularly early in life, influences the immune system, decreasing the risk of inappropriate response to innocuous antigens and subsequent development of asthma and/or allergy [3, 17, 18].

Asthma and other atopic diseases commonly coexist [19] and demonstrate diverse trajectories [20, 21], complicating the establishment of putative risk and protective factors. While wheezing, a common symptom in asthma [22–26], is a predictor of asthma development in early life [27], the cause and course of wheezing vary widely [19], particularly in infancy, when respiratory infections and remission are common [15, 28]. Previous studies examining the association between sibship composition and asthma have produced conflicting findings [29, 30]. The aim of this work was to evaluate the association of (1) the number of siblings (sibship size) and (2) the number of older siblings (birth order) with the risk of asthma, including symptoms of wheezing. Given continuous lifestyle changes in recent decades, we also aimed to elucidate whether the role of sibship composition in asthma reflects these societal transitions by stratifying studies between those published before and after the turn of the millennium. Furthermore, using the World Bank’s classification of countries by income, we evaluated whether the association between sibship composition and asthma varies by socioeconomic development.

## Methods

This study was conducted according to an a priori published protocol [31], which was reported following the Preferred Reporting Items for Systematic Review and Meta-Analysis Protocols (PRISMA-P) [32] and prospectively registered on the International Prospective Register of Systematic Reviews (PROSPERO; CRD42020207905). We reported our work in accordance with the Preferred Reporting Items for Systematic Reviews and Meta-Analyses (PRISMA) [33] checklist (supplementary table S1) and the Meta-analysis of Observational Studies in Epidemiology (MOOSE) [34] reporting guidelines (supplementary table S2).

### Data sources and search strategy

AMED, CABI, CINAHL, Embase, Google Scholar, OAIster, Open Access Theses and Dissertations, Open Grey, ProQuest Dissertations & Theses Global, PsycINFO, PubMed, SciELO, Scopus, Web of Science, and WHO Global Index Medicus were searched from inception through the search date (30 September 2020). An updated search was performed on 20 October 2021. From Google Scholar, the first 300 results were retrieved [35]. Articles in languages other than English were translated using Google Translate [36]. References of included studies were screened for additional studies. The search strategy (supplementary table S3A-I) was developed by DL and BIN.

### Inclusion and exclusion criteria

Observational studies (including cohort studies, case‒control studies, and cross-sectional studies) of any publication status (e.g., preprint, in embargo, or in press) were eligible. Reviews, case series/studies, and expert opinions were excluded. There was no restriction on subject characteristics or sample size. Studies with defined sibship composition as an independent variable and asthma—either self-reported, including symptom-based definitions, e.g., wheezing [37], or based on clinician diagnosis or clinical measurements, e.g., spirometry findings of variable expiratory airflow limitation [38]—as the dependent variable were eligible.

### Study selection and data extraction

Deduplication was performed by DL in EndNote X9 (Clarivate Analytics, 2020) using a method proposed by Bramer et al. [39]. DL and SSÖE independently screened titles/abstracts and assessed the full texts of reports that did not meet any exclusion criteria. After each step, the decisions were unblinded and compared for differences, which were arbitrated by a third reviewer (BIN) if necessary. Data extraction was conducted in pairs (DL, SSÖE, AI, GPM, SN, and GCIS) using an a priori developed data extraction form, following the same methodology. From each article, we extracted the following: first author; year of publication; study design; source of subjects (e.g., medical records or registry); number, age, and country of subjects; definition/assessment of exposure and outcome; and numerical data of findings.

### Quality assessment

Assessment of quality in the included studies was performed using the Effective Public Health Practice Project (EPHPP) [40] tool, modified based on a systematic review by Smith et al. [41]. Six domains (study design, selection bias, confounding, blinding, data collection, and withdrawals/dropouts) were rated as “strong”, “moderate”, or “weak”. The overall rating was based on the number of “weak” domain ratings: “weak” (≥ 2 “weak” ratings), “moderate” (1 “weak” rating), and “strong” (no “weak” ratings). Pairs of reviewers (DL, SSÖE, AI, GPM, SN, and GCIS) independently assessed quality. The ratings were unblinded after completion and compared for differences, which were arbitrated by a third reviewer (BIN) if necessary.

### Data synthesis and statistical analysis

Descriptive tables summarizing key characteristics of the included studies were generated. Findings were narratively synthesized. Comparable (regarding independent/dependent variables and participant characteristics) numerical data from ≥ 2 separate studies [42] were analyzed using meta-analysis with robust variance estimation (RVE) using the *robu()* function from the *robumeta* [43] R package. RVE can account for statistically dependent estimates, e.g., estimates from individual studies that compare the effect in several (similar) treatment groups against one control group, thereby making use of a larger proportion of available data and facilitating a more comprehensive assessment [44]. In the present study, a common dependency structure was measurements of multiple cardinalities (e.g., sibship sizes) against the same reference group. The correlated effects model, small sample correction (to increase accuracy) [43], and the default rho value of 0.8 were used. Meta-analysis results were presented in forest plots created using the *forest()* function from the *forestploter* [45] R package. Separate meta-analyses were performed for each exposure type (birth order and sibship size) in relation to (a) current asthma (in the last year), (b) ever asthma, (c) any wheezing in the last ≤ 1.5 years, and (d) recurrent wheezing (≥ 2 episodes) in the last ≤ 1.5 years. For sibship size, subjects without siblings constituted the reference group. Similarly, first-born subjects were the reference group for birth order. Risk ratio (RR) was used as measure of effect, due to the exposures’ prospective nature and intuitive interpretation of results [46–48], with 95% confidence interval (95% CI). Data in odds ratio (OR) and hazard ratio (HR) were converted to estimates of RR if outcome was ≥ 15% (at the end of follow-up) [49]:

- RR ≈$$\sqrt{\mathrm{OR}}$$
- RR ≈$$\frac{1-{0.5}^{\sqrt{\mathrm{HR}}}}{1-{0.5}^{\sqrt{\frac{1}{\mathrm{HR}}}}}$$

Incidence risk ratio (IRR), prevalence ratio (PR), and relative risk ratio (RRR) estimates were used without conversion, as these are mathematically identical to RR [46]. Effect sizes were recalculated using the reciprocal of the estimate where the reference exposure was not the lower cardinality, e.g., birth order < 3 vs. ≥ 3. Subgroup analyses were performed to assess potential causes for heterogeneity using the following variables: (a) study design; (b) overall rating; (c) classification of country into “high income”, “upper-middle income”, “lower-middle income”, and “low income” economy, as defined at the year of publication by the World Bank [50]; (d) year(s) during which data were collected, divided into < 2000 and ≥ 2000; (e) continent, divided into Africa, Asia, Europe, North America, Oceania, and South America; (f) participant age (in years), divided arbitrarily into ≤ 1.5 and > 1.5 for wheezing outcomes, to differentiate infants who likely wheeze due to bronchiolitis, as this is a common cause of wheezing in the lower age group [51, 52], and < 6 and ≥ 6 for asthma outcomes, also selected arbitrarily to better differentiate transient obstructive airway disease from genuine asthma, the former more commonly presenting in the lower age group [53, 54]; and (g) exposure cardinality (e.g., sibship size 2). Subgroup analysis was performed in cases of ≥ 4 comparable studies in ≥ 2 subgroups [55].

Sensitivity analysis was conducted by excluding studies with a “weak” overall rating and studies where the outcome was not clinically confirmed (medical records or clinical examination). Furthermore, sensitivity analysis on the basis of the rho value in the meta-analyses was performed using the *sensitivity()* function from the *robumeta* [43] R package, in which the pooled effect size was calculated with increments of 0.2 from 0 to 1. The I-squared (*I*^*2*^) statistic was calculated to quantify the proportion of variance across study estimates not due to random sampling error [56, 57]. Tau-squared (*τ*^2^) was calculated to assess the between-study variance [58]. Meta-analysis results with Satterwhite degrees of freedom (*df*) < 4 were considered unreliable [43].

Publication bias was assessed: (a) visually for indications of asymmetry with funnel plots (using the *funnel()* function); (b) statistically, with Begg and Mazumdar rank correlation test [59] (using the *ranktest()* function) and Egger’s regression test [60] (using the *regtest()* function), regarding *P* < 0.05 as significant. The trim-and-fill method [61] was implemented to assess the number of studies needed to normalize asymmetric funnel plots using the *trimfill()* function. Publication bias assessment was performed with the *metafor* [62] R package in exposure-outcome pairs with ≥ 10 studies [63]. The R scripts and compiled datasets used in the analyses are available at Open Science Framework (https://osf.io/kmfe2).

## Results

**Fig. 1:**
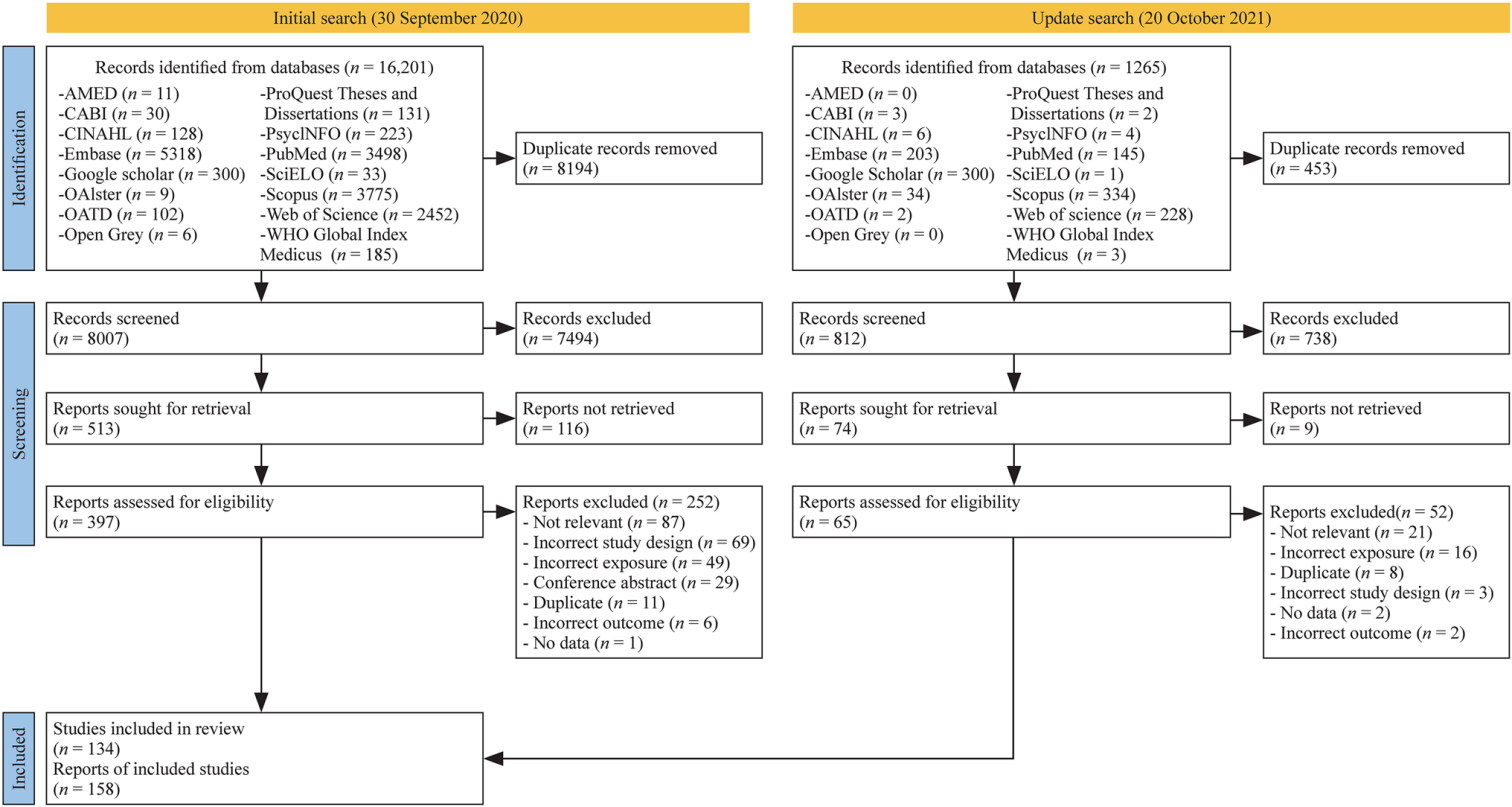
Preferred reporting items for systematic reviews and meta-analyses (PRISMA) flow diagram. http://www.prisma-statement.org/

In total, 17,466 records were identified. Following deduplication, 8819 records proceeded to screening by title/abstract. Of these, 462 full-text reports were assessed for eligibility. A total of 158 reports of 134 studies met the full inclusion criteria (Fig. 1).

### Study characteristics

**Fig. 2:**
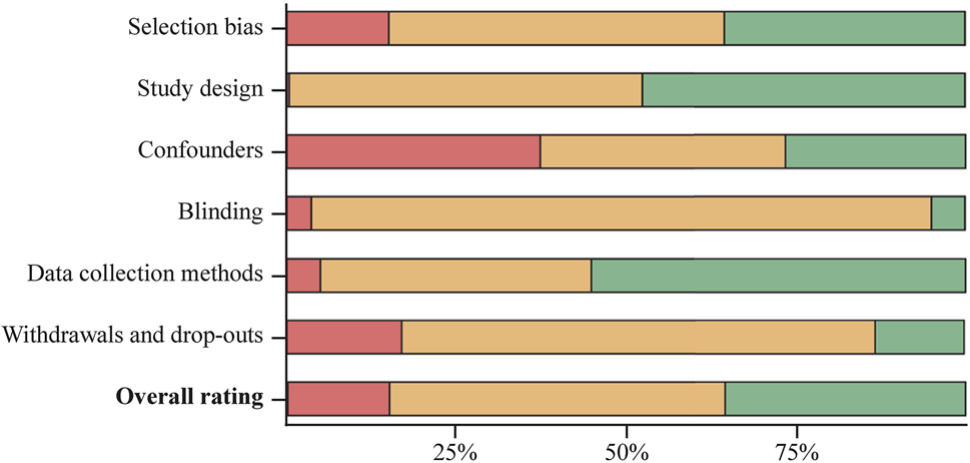
Domain ratings and overall rating of the included studies. Red: “weak”, yellow: “moderate”, green: “strong” rating

**Fig. 3:**
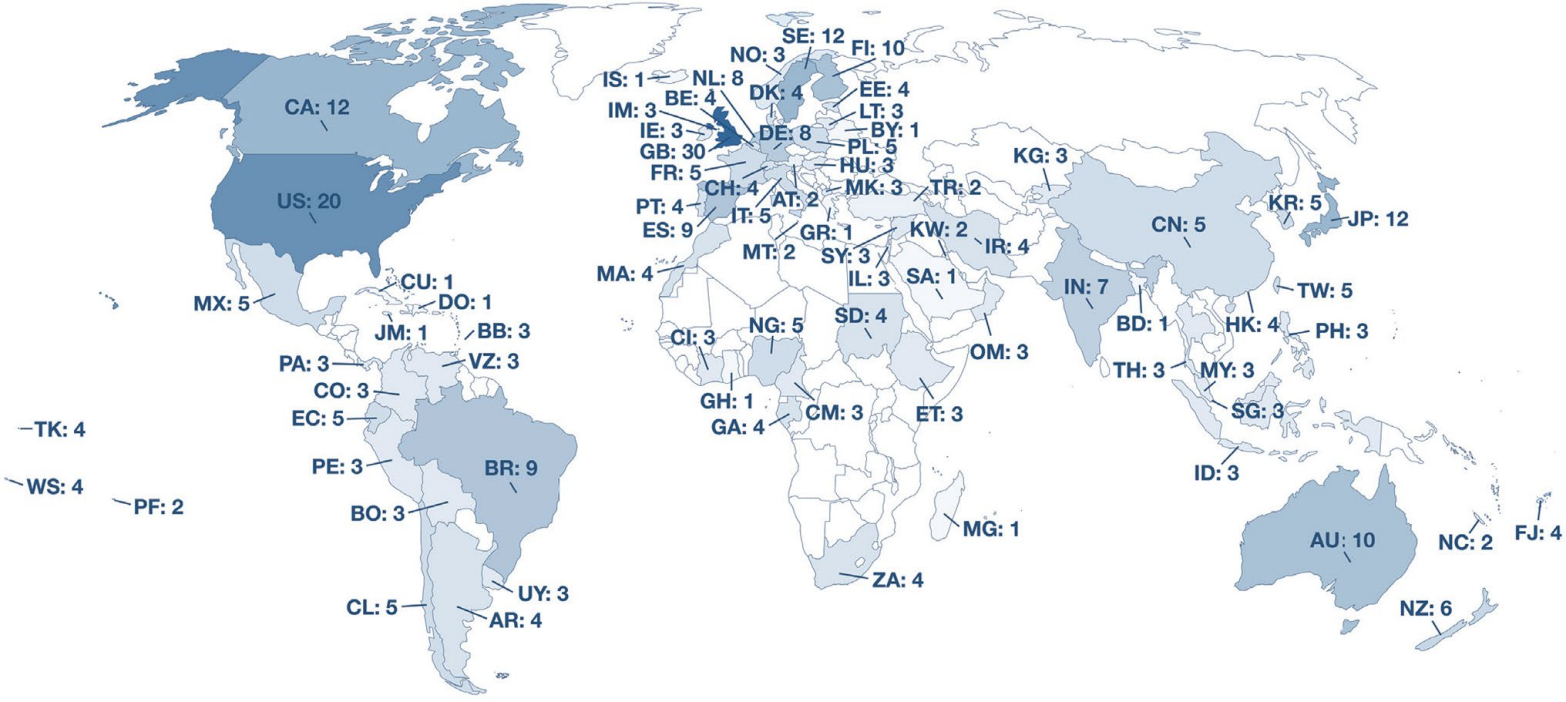
Map of the countries of participants in the included studies. The two letter code indicates the country name and the number indicates how many reports there are from said country. *AR* Argentina, *AT* Austria, *AU* Australia, *BB* Barbados, *BD* Bangladesh, *BE* Belgium, *BO* Bolivia (Plurinational State of), *BR* Brazil, *BY* Belarus, *CA* Canada, *CH* Switzerland, *CI* Côte d'Ivoire, *CL* Chile, *CM* Cameroon, *CN* China, *CO* Colombia, *CU* Cuba, *DE* Germany, *DK* Denmark, *DO* Dominican Republic, *EC* Ecuador, *EE* Estonia, *ES* Spain, *ET* Ethiopia, *FI* Finland, *FJ* Fiji, *FR* France, *GA* Gabon, *GB* United Kingdom of Great Britain and Northern Ireland, *GH* Ghana, *GR* Greece, *HK* Hong Kong, *HU* Hungary, *ID* Indonesia, *IE* Ireland, *IL* Israel, *IM* Isle of Man, *IN* India, *IR* Iran (Islamic Republic of), *IS* Iceland, *IT* Italy, *JM* Jamaica, *JP* Japan, *KG* Kyrgyzstan, KR Korea, Republic of, KW Kuwait, LT Lithuania, MA Morocco, *MG* Madagascar, *MK* North Macedonia, *MT* Malta, *MX* Mexico, *MY* Malaysia, *NC* New Caledonia, *NG* Nigeria, *NL* Netherlands, *NO* Norway, *NZ* New Zealand, *OM* Oman, *PA* Panama, *PE* Peru, *PF* French Polynesia, *PH* Philippines, *PL* Poland, *PT* Portugal, *SA* Saudi Arabia, *SD* Sudan, *SE* Sweden, *SG* Singapore, *SY* Syrian Arab Republic, *TH* Thailand, *TK* Tokelau, *TR* Turkey, *TW* Taiwan, Province of China, *US* United States of America, *UY* Uruguay, *VE* Venezuela (Bolivarian Republic of), *WS* Samoa, *ZA* South Africa

**Fig. 4:**
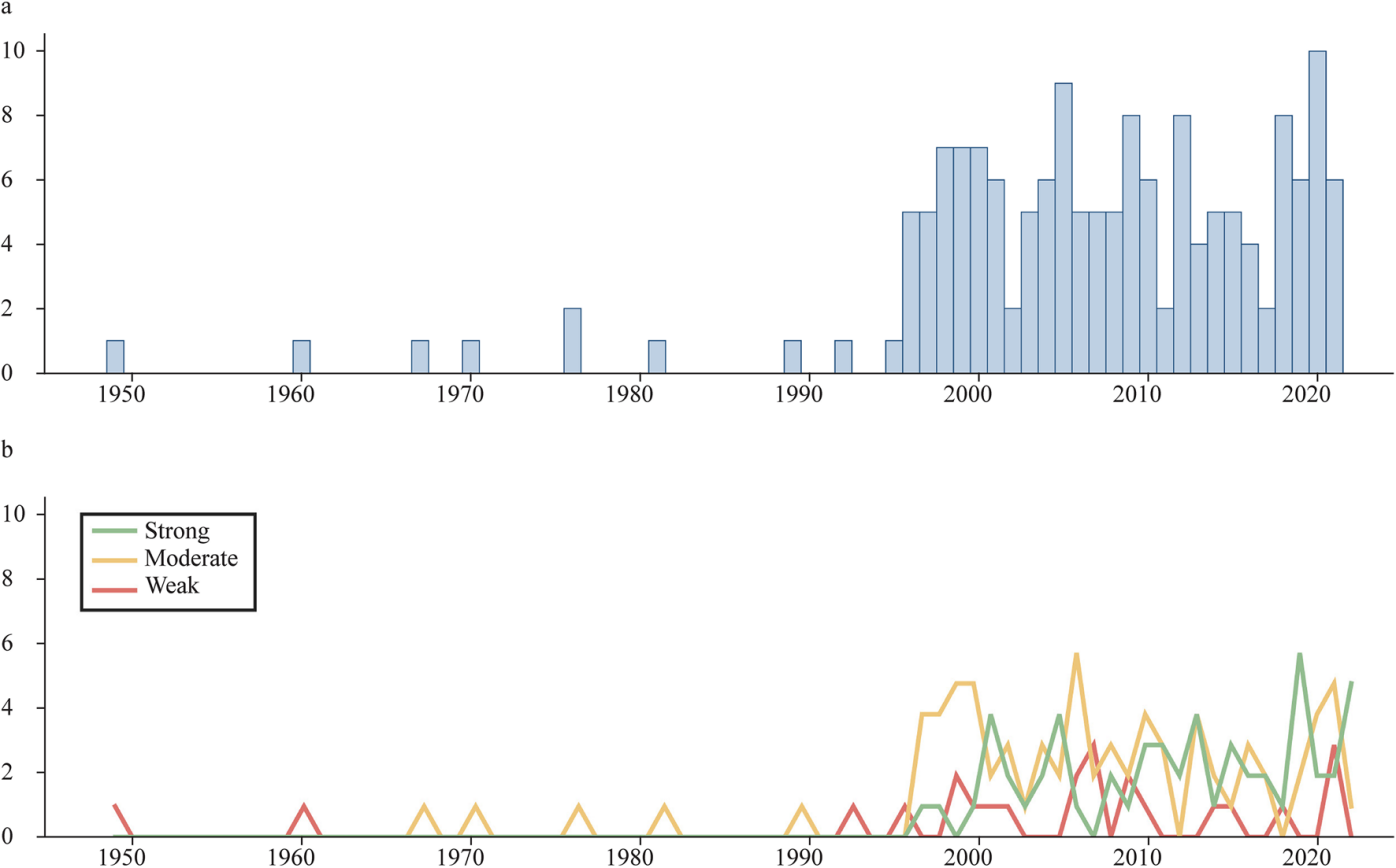
Number of reports published by year among included reports (**a**) and overall rating of the included reports by year (**b**)

The characteristics of the included reports are shown in Supplementary Table S4A-C. Most were cohort *(n* = 70) or cross-sectional (*n* = 71) designs, while 13 were case‒control studies and four were nested case‒control studies. Fifty-six reports had “high”, 78 had “moderate”, and 24 had “weak” overall ratings (Fig. 2, Supplementary Table S5). In total, the studies included data from over 3 million participants across 79 countries (Fig. 3). Most studies were conducted in high-income countries, particularly in Europe, on the American continent, northeastern Asia, and Oceania. Reports with high overall ratings were mostly published in recent times (Fig. 4).

### Any wheezing

**Fig. 5:**
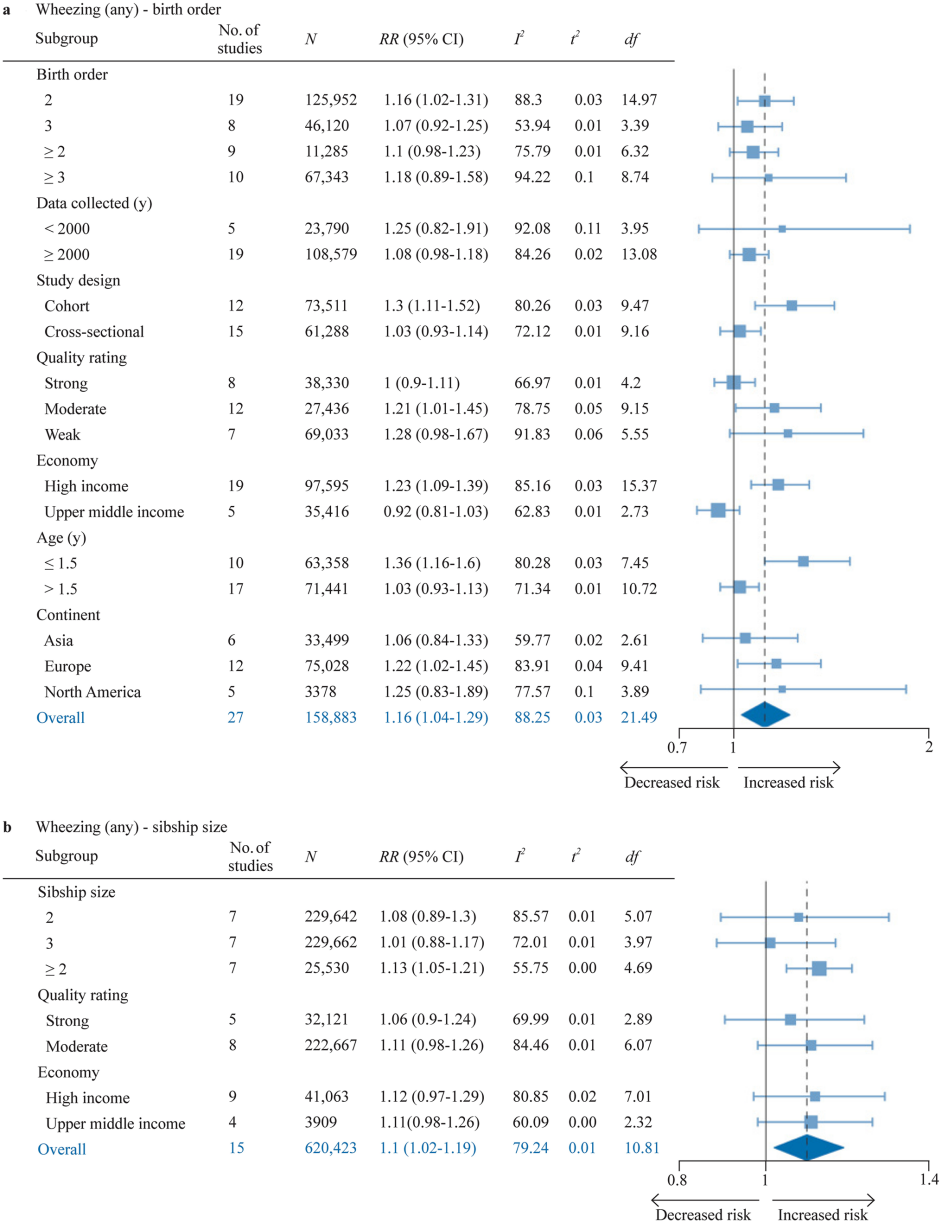
Forest plot for birth order ≥ 2 vs. 1 (**a**) and sibship size ≥ 2 vs 1 (**b**) in relation to any wheezing (≥ 1 episode in last ≤ 1.5 years). *df* Satterwhite degrees of freedom, *K* number of studies, *N* number of subjects (if not available, the number of subjects for the most similar exposure-outcome pair or for the whole study is stated), *No.* number, *RR* risk ratio

Any wheezing was assessed with meta-analysis in 27 reports for birth order and 15 reports for sibship (Fig. 5, Supplementary Figure S1a-b). The pooled effect size for sibship size ≥ 2 vs. 1 indicated significantly increased risk (RR 1.10, 95% CI 1.02–1.19). Similarly, the pooled effect size for birth order ≥ 2 vs. 1 indicated an increased risk (RR 1.16, 95% CI 1.04–1.29). However, the effect only remained significant for subjects aged ≤ 1.5 years (RR 1.36, 95% CI 1.16–1.6). Similarly, the increased risk was only statistically significant in studies conducted in Europe (RR 1.22, 95% CI 1.02–1.45). Finally, a marginally stronger association, albeit with wider 95% CIs, could be seen for birth order ≥ 2 in studies of moderate or low overall quality compared to studies of high overall quality. Heterogeneity was high for both sibship size (*I*^*2*^ = 79.2%, *τ*^2^ = 0.01) and birth order (*I*^*2*^ = 88.3%, *τ*^2^ = 0.03).

### Recurrent wheezing

Recurrent wheezing was assessed with meta-analysis in five reports for birth order and three reports for sibship size (Supplementary Figure S2a-b). There were insufficient studies to perform subgroup analysis, and the pooled effect sizes were nonsignificant. Heterogeneity was moderate for both birth order ≥ 2 vs. 1 (*I*^*2*^ = 72.8%, *τ*^2^ = 0.13) and sibship size ≥ 2 vs. 1 (*I*^*2*^ = 72.7%, *τ*^2^ = 0.08).

### Current asthma

**Fig. 6:**
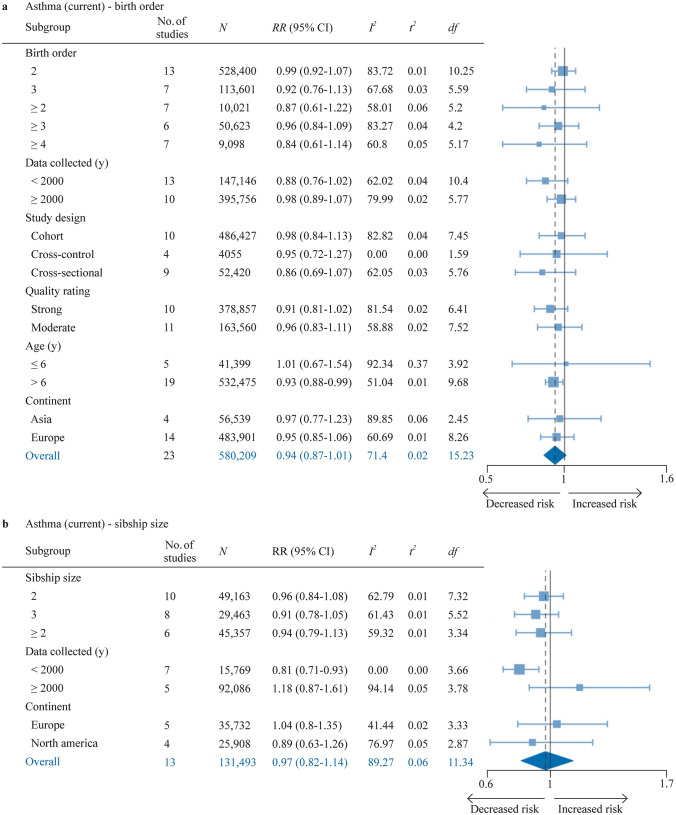
Forest plot for birth order ≥ 2 vs. 1 (**a**) and sibship size ≥ 2 vs. 1 (**b**) in relation to current asthma (in last year). *df* Satterwhite degrees of freedom, *K* number of studies, *N* number of subjects (if not available, the number of subjects for the most similar exposure-outcome pair or for the whole study is stated), *No.* number, *RR* risk ratio

Current asthma was assessed with meta-analysis in 23 reports for birth order and 13 reports for sibship size (Fig. 6, Supplementary Figure S3a-b). The pooled effect sizes for sibship size ≥ 2 vs. 1 and birth order ≥ 2 vs. 1 were nonsignificant. However, for subjects aged ≥ 6 years, having ≥ 1 older sibling was associated with a marginally reduced risk (RR 0.94, 95% CI 0.88–0.99; *I*^*2*^ = 51.0%, *τ*^2^ = 0.01). A dose-dependent increase could be discerned in the subgroup analysis for birth order, but none of the cardinalities had a significant pooled effect size. The association did not vary notably by overall quality. For sibship size ≥ 2 vs. 1, the only significant finding was for studies published before 2000 (RR 0.81, 95% CI 0.71–0.93; *I*^2^ = 0%, *τ*^2^ = 0).

### Ever asthma

**Fig. 7:**
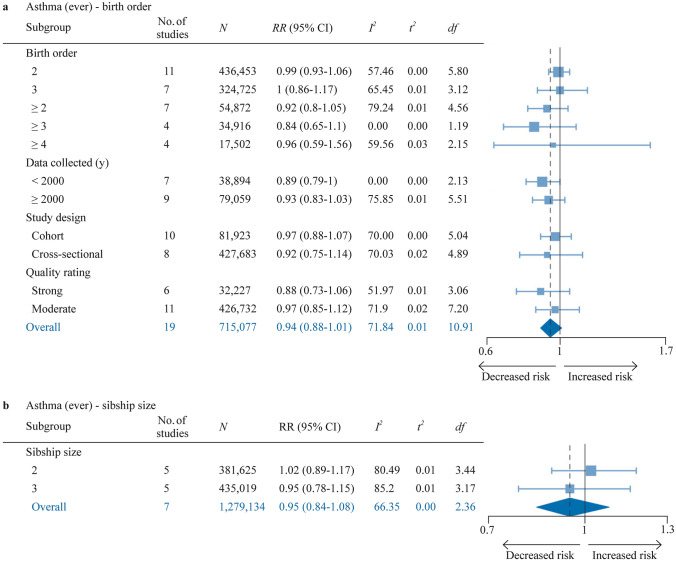
Forest plot for birth order ≥ 2 vs. 1 (**a**) and sibship size ≥ 2 vs. 1 (**b**) in relation to ever asthma. *df* Satterwhite degrees of freedom, *K* number of studies, *N* number of subjects (if not available, the number of subjects for the most similar exposure-outcome pair or for the whole study is stated), *No.* number, *RR* risk ratio

Ever asthma was assessed with meta-analysis in 19 reports for birth order and 7 reports for sibship size (Fig. 7, Supplementary Figure S4a-b). None of the pooled effect sizes were significant, and subgroup analyses did not produce any significant findings. A slight trend of weakening association with time could potentially be discerned for birth order, with the pooled effect size on the edge of being significant – albeit with *df* = 2.13—for studies published before 2000 (RR 0.89, 95% CI 0.79–1) compared to later studies (RR 0.93, 95% CI 0.83–1.03). Studies of strong overall quality indicated a slightly stronger association than studies of moderate overall quality. Heterogeneity was moderate for both birth order (*I*^*2*^ = 71.8%, *τ*^2^ = 0.01) and sibship size (*I*^*2*^ = 66.4%, *τ*^2^ = 0).

### Publication bias and sensitivity analysis

The funnel plots (Supplementary Fig. S5) did not indicate substantial publication bias. For birth order on current asthma, Egger’s regression test was significant (*P* = 0.002; Supplementary Table S6c), and the corresponding funnel plot appeared asymmetric; however, the outlying estimates were mostly derived from one study [64] that reported on multiple outcomes. Similarly, the funnel plot for sibship size on current asthma after trim-and-fill did not indicate any genuine publication bias (Supplementary Fig. S6b).

When excluding studies with a “weak” overall rating, the effect of birth order ≥ 2 vs. 1 on any wheezing became barely nonsignificant (RR 1.12, 95% CI 0.99–1.26) compared to the pooled effect size of all studies (RR 1.16, 95% CI 1.04–1.29), while the association of sibship size with any wheezing remained significant. For asthma outcomes, the overall pooled effect sizes remained similar across sensitivity analyses, while there were too few studies on recurrent wheezing to draw any conclusions (Supplementary Table S7). Different values of rho only marginally shifted the pooled effect sizes (Supplementary Table S8).

## Discussion

### Summary of key findings

We found a slight but significantly increased risk of wheezing in infants with siblings and second-born or later infants. This association was not significant for recurrent wheezing and did not remain beyond infancy; however, the risk of current asthma was marginally lower for individuals aged ≥ 6 years with at least one older sibling. The investigated associations weakened in studies published after 2000 compared to earlier studies. This trend was also seen in studies of moderate or strong overall rating from before vs. after the turn of the millennium. The findings were comparable between continents for most outcomes; however, for the association of birth order with any wheezing, a statistically significant increased risk was indicated in Europe, in contrast to Asia and North America.

### Strengths and limitations

To our knowledge, this systematic review is the first to assess the association between sibship composition and the risk of asthma. We searched 15 databases and identified a substantial body of relevant research, allowing for precise meta-analysis and detailed analyses of trends and associations at the subgroup level. However, most studies were from high-income countries, which limited subgroup analysis by income. Similarly, as most studies were conducted in geographically limited areas, we had insufficient data to discern any clear and consistent trend or difference in the association by continent. Furthermore, the included studies were heterogeneous in methodology, participants, and definition of asthma, restricting generalizability of the results. This also limited the number of studies eligible for meta-analysis, particularly due to substantial differences in the cardinalities (e.g., sibship size) and reference groups used, as well as heterogeneous outcome definitions, e.g., with variations in healthcare use, concomitant symptoms such as wheezing, presence of atopy, etc. Observational studies—constituting the basis for our analyses—are prone to risk of confounding [65]; thus, the findings may not indicate a true causal effect of sibship composition on risk of asthma. Finally, most studies used self-reports of investigated outcomes, which may have reduced precision and clinical validity [54, 66–68].

### Comparison of findings to previous studies

To the best of our knowledge, this is the first systematic review on the role of sibship composition in asthma. Furthermore, we performed the first quantitative synthesis including dependent data for this association, enabling precise pooled effect size estimates.

### Interpretation of findings

Between birth order and sibship size in relation to the risk of asthma, the impact of birth order appears stronger than that of sibship size, which may be because being second-born presupposes having at least one sibling, while having at least one sibling does not presuppose having at least one older sibling. Furthermore, the proposed “hygiene hypothesis” is likely driven by older siblings, who may be old enough to attend school or have outdoor activities from which contracted infections can be transmitted to younger siblings [64, 69, 70]. While the association of sibship composition with allergy appears more consistent, the weaker results for asthma can perhaps be explained by the heterogeneous nature of asthma, with inconsistent diagnosis and classification during childhood. As a heterogeneous disease, asthma consists of multiple endotypes and phenotypes [71, 72], with varying underlying mechanisms and influencing factors [1], some of which do not involve allergic components [73, 74]. These factors significantly complicate the interpretation of our findings regarding asthma as an outcome. Although most of the studies were conducted in a small number of countries, subgroup analysis by continent indicated that some differences in the association may exist, at least for wheezing, possibly explained by differences in exposure or lifestyle factors between geographical regions. Finally, it appears as if the association of sibship composition with risk of asthma and wheezing is diminishing over time, as pooled effect sizes weakened in studies from year ≥ 2000 compared with earlier studies. This could be related to socioeconomic and lifestyle changes, e.g., more children attending daycare [75], or other factors influencing the risk of asthma that we did not have sufficient data to account for, such as air pollution, which varies substantially by region and time [76], but this trend could also be an indication of improved diagnosis of asthma, with newer studies commonly implementing more rigorous and accurate assessment methods.

### Clinical and research implications

The association between sibship composition and transient wheezing appears to stem from respiratory infections, commonly caused by cross-infection between siblings during infancy [51, 77], and does persist into childhood, during which wheezing is more commonly caused by obstructive airway disease. Current asthma, however, was marginally less common among second-born or later subjects aged ≥ 6 years. Given the subtle difference in risk, together with the heterogeneity of the included studies, the complexity of the disease, and the seemingly weakening effect in recent decades, the protection of having older siblings may not constitute a protection of relevance in practice.

In conclusion, our findings indicate that having siblings and being second-born or later, respectively, may constitute a slightly increased risk of transient wheezing in infancy. This association does not extend beyond infancy. In contrast, being second-born or later appears to be associated with marginal protection against asthma. These associations have seemingly weakened since the turn of the millennium, possibly due to lifestyle changes and socioeconomic development.

### Supplementary Information

Below is the link to the electronic supplementary material.

Supplementary file1 (DOCX 3823 KB)

## Data Availability

All data and code used for the analyses are available at https://osf.io/kmfe2/.
