# Supplementary material for: Is sibship composition a risk factor for childhood asthma? Systematic review and meta-analysis
Source: World J Pediatr. 2023 Mar 30;19(12):1127–38. doi: 10.1007/s12519-023-00706-w (PMC10590346; doi:10.1007/s12519-023-00706-w)
Supplement: Supplementary file 2 — Supplementary file1 (DOCX 3823 KB) [file 12519_2023_706_MOESM1_ESM.docx]

**Is sibship composition a risk factor for childhood asthma? Systematic review and meta-analysis of the global literature**

**Supplementary material**

[Tables 3](#_Toc116592328)

[Table S1. Preferred Reporting Items for Systematic Reviews and Meta-Analyses (PRISMA) 2020 checklist 3](#_Toc116592329)

[Table S2. Meta-analyses Of Observational Studies in Epidemiology (MOOSE) checklist 5](#_Toc116592330)

[Search strategy 7](#_Toc116592331)

[Table S3A. Search strategy for AMED 7](#_Toc116592332)

[Table S3B. Search strategy for: CABI; OAIster; Open Access Theses and Dissertations; Open Grey; ProQuest Dissertations & Theses Global; SciELO; WHO Global Index Medicus 8](#_Toc116592333)

[Table S3C. Search strategy for CINAHL 9](#_Toc116592334)

[Table S3D. Search strategy for EMBASE 10](#_Toc116592335)

[Table S3E. Search strategy for Google Scholar 11](#_Toc116592336)

[Table S3F. Search strategy for PsycINFO 11](#_Toc116592337)

[Table S3G. Search strategy for PubMed 13](#_Toc116592338)

[Table S3H. Search strategy for Scopus 14](#_Toc116592339)

[Table S3I. Search strategy for Web of Science 15](#_Toc116592340)

[Tables of characteristics 16](#_Toc116592341)

[Table S4A. Table of characteristics for asthma 16](#_Toc116592342)

[Table S4B. Table of characteristics for wheezing 26](#_Toc116592343)

[Table S4C. Table of characteristics for other asthma-related outcomes 36](#_Toc116592344)

[Table S5. Quality appraisal 38](#_Toc116592345)

[Table S6. Publication bias 43](#_Toc116592346)

[Table S7. Sensitivity analysis (subgroups) 44](#_Toc116592347)

[Table S8. Sensitivity analysis (rho) 44](#_Toc116592348)

[Excluded studies 46](#_Toc116592349)

[Table S9A. Excluded studies (initial search) 46](#_Toc116592350)

[Table S9B. Excluded studies (update search) 65](#_Toc116592351)

[Figures 69](#_Toc116592352)

[Figure S1A. Forest plot (birth order on any wheezing) 69](#_Toc116592353)

[Figure S1B. Forest plot (sibship size on any wheezing) 70](#_Toc116592354)

[Figure S2A. Forest plot (birth order on recurrent wheezing) 71](#_Toc116592355)

[Figure S2B. Forest plot (sibship size on recurrent wheezing) 72](#_Toc116592356)

[Figure S3A. Forest plot (birth order on current asthma) 73](#_Toc116592357)

[Figure S3B. Forest plot (sibship size on current asthma) 74](#_Toc116592358)

[Figure S4A. Forest plot (birth order on ever asthma) 75](#_Toc116592359)

[Figure S4B. Forest plot (sibship size on ever asthma) 76](#_Toc116592360)

[Figure S5. Funnel plots (prior to trim-and-fill) 77](#_Toc116592361)

[Figure S6. Funnel plots (after trim-and-fill) 78](#_Toc116592362)

[References to all included studies 79](#_Toc116592363)

# Tables

## Table S1. Preferred Reporting Items for Systematic Reviews and Meta-Analyses (PRISMA) 2020 checklist

| **Section and Topic** | **Item #** | **Checklist item** | **Location where item is reported** |
| --- | --- | --- | --- |
| **TITLE** | | |  |
| Title | 1 | Identify the report as a systematic review. | Title/first page (p. 1) |
| **ABSTRACT** | | |  |
| Abstract | 2 | See the PRISMA 2020 for Abstracts checklist. | Abstract (p. 2) |
| **INTRODUCTION** | | |  |
| Rationale | 3 | Describe the rationale for the review in the context of existing knowledge. | Introduction (p. 3-4) |
| Objectives | 4 | Provide an explicit statement of the objective(s) or question(s) the review addresses. | Introduction (p. 4) |
| **METHODS** | | |  |
| Eligibility criteria | 5 | Specify the inclusion and exclusion criteria for the review and how studies were grouped for the syntheses. | Methods > Inclusion and exclusion criteria (p. 5) |
| Information sources | 6 | Specify all databases, registers, websites, organisations, reference lists and other sources searched or consulted to identify studies. Specify the date when each source was last searched or consulted. | Methods > Data sources and search strategy (p. 5) |
| Search strategy | 7 | Present the full search strategies for all databases, registers and websites, including any filters and limits used. | Methods > Data sources and search strategy (p. 5) |
| Selection process | 8 | Specify the methods used to decide whether a study met the inclusion criteria of the review, including how many reviewers screened each record and each report retrieved, whether they worked independently, and if applicable, details of automation tools used in the process. | Methods > Study selection and data extraction (p. 5-6) |
| Data collection process | 9 | Specify the methods used to collect data from reports, including how many reviewers collected data from each report, whether they worked independently, any processes for obtaining or confirming data from study investigators, and if applicable, details of automation tools used in the process. | Methods > Study selection and data extraction (p. 5-6) |
| Data items | 10a | List and define all outcomes for which data were sought. Specify whether all results that were compatible with each outcome domain in each study were sought (e.g. for all measures, time points, analyses), and if not, the methods used to decide which results to collect. | Methods > Study selection and data extraction (p. 6) |
|  | 10b | List and define all other variables for which data were sought (e.g. participant and intervention characteristics, funding sources). Describe any assumptions made about any missing or unclear information. | Methods > Study selection and data extraction (p. 6) |
| Study risk of bias assessment | 11 | Specify the methods used to assess risk of bias in the included studies, including details of the tool(s) used, how many reviewers assessed each study and whether they worked independently, and if applicable, details of automation tools used in the process. | Methods > Quality assessment (p. 6) |
| Effect measures | 12 | Specify for each outcome the effect measure(s) (e.g. risk ratio, mean difference) used in the synthesis or presentation of results. | Methods > Data synthesis and statistical analysis (p. 7) |
| Synthesis methods | 13a | Describe the processes used to decide which studies were eligible for each synthesis (e.g. tabulating the study intervention characteristics and comparing against the planned groups for each synthesis (item #5)). | Methods > Data synthesis and statistical analysis (p. 6-9) |
|  | 13b | Describe any methods required to prepare the data for presentation or synthesis, such as handling of missing summary statistics, or data conversions. | Methods > Data synthesis and statistical analysis (p. 7) |
|  | 13c | Describe any methods used to tabulate or visually display results of individual studies and syntheses. | Methods > Data synthesis and statistical analysis (p. 6-9) |
|  | 13d | Describe any methods used to synthesize results and provide a rationale for the choice(s). If meta-analysis was performed, describe the model(s), method(s) to identify the presence and extent of statistical heterogeneity, and software package(s) used. | Methods > Data synthesis and statistical analysis (p. 6-7) |
|  | 13e | Describe any methods used to explore possible causes of heterogeneity among study results (e.g. subgroup analysis, meta-regression). | Methods > Data synthesis and statistical analysis (p. 8) |
|  | 13f | Describe any sensitivity analyses conducted to assess robustness of the synthesized results. | Methods > Data synthesis and statistical analysis (p. 8-9) |
| Reporting bias assessment | 14 | Describe any methods used to assess risk of bias due to missing results in a synthesis (arising from reporting biases). | Methods > Data synthesis and statistical analysis (p. 9) |
| Certainty assessment | 15 | Describe any methods used to assess certainty (or confidence) in the body of evidence for an outcome. | Methods > Data synthesis and statistical analysis (p. 8) |
| **RESULTS** | | |  |
| Study selection | 16a | Describe the results of the search and selection process, from the number of records identified in the search to the number of studies included in the review, ideally using a flow diagram. | Results (p. 9) |
|  | 16b | Cite studies that might appear to meet the inclusion criteria, but which were excluded, and explain why they were excluded. | Supplementary table S9A-B |
| Study characteristics | 17 | Cite each included study and present its characteristics. | Supplementary material (“References to all included studies”) |
| Risk of bias in studies | 18 | Present assessments of risk of bias for each included study. | Supplementary table S5 |
| Results of individual studies | 19 | For all outcomes, present, for each study: (a) summary statistics for each group (where appropriate) and (b) an effect estimate and its precision (e.g. confidence/credible interval), ideally using structured tables or plots. | Figure 5-7, supplementary figure S1A-4B, supplementary table S4A-C |
| Results of syntheses | 20a | For each synthesis, briefly summarise the characteristics and risk of bias among contributing studies. | Results > Any wheezing (p. 10), Results > Recurrent wheezing (p. 10), Results > Current asthma (p. 10-11), Results > Ever asthma (p. 11), figure 5-7, supplementary figure S1A-4B |
|  | 20b | Present results of all statistical syntheses conducted. If meta-analysis was done, present for each the summary estimate and its precision (e.g. confidence/credible interval) and measures of statistical heterogeneity. If comparing groups, describe the direction of the effect. | Results > Any wheezing (p. 10), Results > Recurrent wheezing (p. 10), Results > Current asthma (p. 10-11), Results > Ever asthma (p. 11), figure 5-7, supplementary figure S1A-4B, supplementary table S4A-C |
|  | 20c | Present results of all investigations of possible causes of heterogeneity among study results. | Results, figure 5-7, supplementary figure S1A-4B, supplementary table S7 |
|  | 20d | Present results of all sensitivity analyses conducted to assess the robustness of the synthesized results. | Results > Publication bias and sensitivity analysis (p. 12), supplementary table S7-8 |
| Reporting biases | 21 | Present assessments of risk of bias due to missing results (arising from reporting biases) for each synthesis assessed. | Results > Publication bias and sensitivity analysis (p. 11-12), supplementary figure S5-6 |
| Certainty of evidence | 22 | Present assessments of certainty (or confidence) in the body of evidence for each outcome assessed. | Discussion > Strengths and limitations (p. 13), Discussion > Clinical and research implications (p. 14) |
| **DISCUSSION** | | |  |
| Discussion | 23a | Provide a general interpretation of the results in the context of other evidence. | Discussion > Comparison of findings to previous studies (p. 13) |
|  | 23b | Discuss any limitations of the evidence included in the review. | Discussion > Strengths and limitations (p. 12-13) |
|  | 23c | Discuss any limitations of the review processes used. | Discussion > Strengths and limitations (p. 12) |
|  | 23d | Discuss implications of the results for practice, policy, and future research. | Discussion > Clinical and research implications (p. 14) |
| **OTHER INFORMATION** | | |  |
| Registration and protocol | 24a | Provide registration information for the review, including register name and registration number, or state that the review was not registered. | Methods (p. 4) |
|  | 24b | Indicate where the review protocol can be accessed, or state that a protocol was not prepared. | Methods (p. 4) |
|  | 24c | Describe and explain any amendments to information provided at registration or in the protocol. | Discussion > Strengths and limitations (p. 13) |
| Support | 25 | Describe sources of financial or non-financial support for the review, and the role of the funders or sponsors in the review. | Acknowledgements (p. 15) |
| Competing interests | 26 | Declare any competing interests of review authors. | Conflict of interest (p. 15) |
| Availability of data, code and other materials | 27 | Report which of the following are publicly available and where they can be found: template data collection forms; data extracted from included studies; data used for all analyses; analytic code; any other materials used in the review. | Methods > Data synthesis and statistical analysis (p. 9) |

*From:*  Page MJ, McKenzie JE, Bossuyt PM, Boutron I, Hoffmann TC, Mulrow CD, et al. The PRISMA 2020 statement: an updated guideline for reporting systematic reviews. BMJ 2021;372:n71. doi: 10.1136/bmj.n71. For more information, visit: <http://www.prisma-statement.org/>.

##

## Table S2. Meta-analyses Of Observational Studies in Epidemiology (MOOSE) checklist

| **Item** | **Page number** |
| --- | --- |
| **Reporting of background** | |
| Problem definition | 3-4 |
| Hypothesis statement | 3 |
| Description of study outcome(s) | 4-5 |
| Type of exposure or intervention used | 4-5 |
| Type of study designs used | 5 |
| Study population | 5 |
| **Reporting of search strategy** | |
| Qualifications of searchers (eg, librarians and investigators) | N/A |
| Search strategy, including time period included in the synthesis and key words | 4-5 |
| Effort to include all available studies, including contact with authors | 4-5 |
| Databases and registries searched | 4-5 |
| Search software used, name and version, including special features used (eg, explosion) | Supplementary table S3A-I |
| Use of hand searching (eg, reference lists of obtained articles) | 5 |
| List of citations located and those excluded, including justification | Supplementary table S9A-B, supplementary material (“References to all included studies”) |
| Method of addressing articles published in languages other than English | 5 |
| Method of handling abstracts and unpublished studies | 5 |
| Description of any contact with authors | N/A |
| **Reporting of methods** | |
| Description of relevance or appropriateness of studies assembled for assessing the hypothesis to be tested | 5 |
| Rationale for the selection and coding of data (eg, sound clinical principles or convenience) | 6 |
| Documentation of how data were classified and coded (eg, multiple raters, blinding and interrater reliability) | 6 |
| Assessment of confounding (eg, comparability of cases and controls in studies where appropriate) | 7-8 |
| Assessment of study quality, including blinding of quality assessors, stratification or regression on possible predictors of study results | 6 |
| Assessment of heterogeneity | 7-8 |
| Description of statistical methods (eg, complete description of fixed or random effects models, justification of whether the chosen models account for predictors of study results, dose-response models, or cumulative meta-analysis) in sufficient detail to be replicated | 6-9 |
| Provision of appropriate tables and graphics | 6-9, figure 1-7, supplementary material |
| **Reporting of results** | |
| Graphic summarizing individual study estimates and overall estimate | Figure 5-7, supplementary figure S1A-4B |
| Table giving descriptive information for each study included | Supplementary table S4A-C |
| Results of sensitivity testing (eg, subgroup analysis) | 10-11, Figure 5-7, supplementary table S7 |
| Indication of statistical uncertainty of findings | Supplementary table S8 |
| **Reporting of discussion** | |
| Quantitative assessment of bias (eg, publication bias) | 11-12, supplementary figure S5-6, supplementary table S6 |
| Justification for exclusion (eg, exclusion of non-English language citations) | 9, figure 1, supplementary table S9A-B |
| Assessment of quality of included studies | 9, 12-13, figure 2-4, supplementary table S5 |
| **Reporting of conclusions** | |
| Consideration of alternative explanations for observed results | 13-14 |
| Generalization of the conclusions (ie, appropriate for the data presented and within the domain of the literature review) | 14 |
| Guidelines for future research | N/A |
| Disclosure of funding source | 15 |

*From*: Stroup DF, Berlin JA, Morton SC, et al, for the Meta-analysis Of Observational Studies in Epidemiology (MOOSE) Group. Meta-analysis of Observational Studies in Epidemiology. A Proposal for Reporting. *JAMA*. 2000;283(15):2008-2012. doi: 10.1001/jama.283.15.2008.

## Search strategy

**Colorization**

Red: controlled vocabulary/thesaurus

Blue: free-text

Green: referral to search query component (table row; #)

### Table S3A. Search strategy for AMED

| **#** | **Search term(s)** |
| --- | --- |
| 1 | (birth order* or birth rank* or multiple birth* or parity).mp. |
| 2 | exp Family Characteristics/ or (family characteristic* or family size* or family structure* or family demograph* or family composition or household size* or household demograph* or household composition).mp. |
| 3 | exp Sibling Relations/ or (sibling* or sister* or brother* or sibship size* or sibship*).mp. |
| 4 | or/1-3 |
| 5 | exp Asthma/ or (bronchial asthma* or exercise-induced asthma* or exercise-induced bronchospasm* or asthma* or respiratory hypersensitivit* or airway hyper responsiveness or airway hyperresponsiveness or respiratory hyper responsiveness or respiratory hyper-responsiveness or wheez*).mp. |
| 6 | exp Hypersensitivity/ or exp Hypersensitivity Immediate/ or exp Hypersensitivity Delayed/ or (immediate hypersensitivit* or delayed hypersensitivit* or IgE-mediated hypersensitivit* or type I hypersensitivit* or type IV hypersensitivit* or atopic sensitization or atop* or allergic sensitization or allerg*).mp. |
| 7 | exp Dermatitis/ or exp Anaphylaxis/ or (atopic dermatitis or dermatitis or neurodermatiti* or besniers prurigo or besnier prurigo or atopic eczema or eczema or urticari* or anaphyla* or quinckes edema or quincke edema or angioneurotic edema or angioedema or hives).mp. |
| 8 | exp Food Hypersensitivity/ or (food hypersensitivit* or food allerg* or egg hypersensitivit* or egg allerg* or milk hypersensitivit* or milk allerg* or shellfish hypersensitivit* or shellfish allerg* or wheat hypersensitivit* or wheat allerg* or nut hypersensitivit* or nut allerg* or peanut hypersensitivit* or peanut allerg* or groundnut hypersensitivit* or groundnut allerg*).mp. |
| 9 | exp Rhinitis/ or exp Conjunctivitis/ or (allergic rhinoconjunctiviti* or rhinoconjunctiviti* or allergic rhiniti* or seasonal allergic rhiniti* or perennial allergic rhiniti* or rhiniti* or allergic conjunctiviti* or vernal keratoconjunctiviti* or vernal conjunctiviti* or giant papillary conjunctiviti* or hay fever or hayfever or pollinosis or nasal catarrh*).mp. |
| 10 | or/5-9 |
| 11 | 4 and 10 |
| **Full query**  ((birth order* or birth rank* or multiple birth* or parity).mp. or exp Family Characteristics/ or (family characteristic* or family size* or family structure* or family demograph* or family composition or household size* or household demograph* or household composition).mp. or exp Sibling Relations/ or (sibling* or sister* or brother* or sibship size* or sibship*).mp.) and (exp Asthma/ or (bronchial asthma* or exercise-induced asthma* or exercise-induced bronchospasm* or asthma* or respiratory hypersensitivit* or airway hyper responsiveness or airway hyperresponsiveness or respiratory hyper responsiveness or respiratory hyper-responsiveness or wheez*).mp. or exp  Hypersensitivity/ or exp Hypersensitivity Immediate/ or exp Hypersensitivity Delayed/ or (immediate hypersensitivit* or delayed hypersensitivit* or IgE-mediated hypersensitivit* or type I hypersensitivit* or type IV hypersensitivit* or atopic sensitization or atop* or allergic sensitization or allerg*).mp. or exp Dermatitis/ or exp Anaphylaxis/ or (atopic dermatitis or dermatitis or neurodermatiti* or besniers prurigo or besnier prurigo or atopic eczema or eczema or urticari* or anaphyla* or quinckes edema or quincke edema or angioneurotic edema or angioedema or hives).mp. or exp Food Hypersensitivity/ or (food hypersensitivit* or food allerg* or egg hypersensitivit* or egg allerg* or milk hypersensitivit* or milk allerg* or shellfish hypersensitivit* or shellfish allerg* or wheat hypersensitivit* or wheat allerg* or nut hypersensitivit* or nut allerg* or peanut hypersensitivit* or peanut allerg* or groundnut hypersensitivit* or groundnut allerg*).mp. or exp Rhinitis/ or exp Conjunctivitis/ or (allergic rhinoconjunctiviti* or rhinoconjunctiviti* or allergic rhiniti* or seasonal allergic rhiniti* or perennial allergic rhiniti* or rhiniti* or allergic conjunctiviti* or vernal keratoconjunctiviti* or vernal conjunctiviti* or giant papillary conjunctiviti* or hay fever or hayfever or pollinosis or nasal catarrh*).mp.) | |

exp = include all narrower subject headings; mp= abstract, heading words, title

### Table S3B. Search strategy for: CABI; OAIster; Open Access Theses and Dissertations; Open Grey; ProQuest Dissertations & Theses Global; SciELO; WHO Global Index Medicus

| **#** | **Search term(s)** |
| --- | --- |
| 1 | "birth order" OR "multiple births" OR "birth rank" OR "parity" |
| 2 | "family characteristics" OR "family size" OR "family structure" OR "family demography" OR "family composition" OR "household size" OR "household demography" OR "household composition" |
| 3 | "siblings" OR "sibling relations" OR "sister" OR "brother" OR "sibship" |
| 4 | "exercise-induced bronchospasm" OR "asthma" OR "airway hyper-responsiveness" OR "respiratory hyper-responsiveness" OR "wheeze" OR "wheezing" |
| 5 | ”hypersensitivity" OR "atopic sensitization" OR "atopy" OR "allergic sensitization" OR "allergic disease" OR "allergic condition" OR "allergy" OR "allergies" |
| 6 | "dermatitis" OR "eczema" OR "neurodermatitis" OR "besnier’s prurigo" OR "urticaria" OR "anaphylaxis" OR "anaphylactic shock" OR "quincke’s edema" OR "angionuerotic edema" OR "angioedema" OR "hives" |
| 7 | "rhinoconjunctivitis" OR "rhinitis" OR "allergic conjunctivitis" OR "vernal keratoconjunctivitis" OR "vernal conjunctivitis" OR "giant papillary conjunctivitis" OR "hay fever" OR "pollinosis" OR "pollenosis" OR "nasal catarrh" |
| 8 | 1 OR 2 OR 3 |
| 9 | 4 OR 5 OR 6 OR 7 |
| 10 | 8 AND 9 |
| **Full query**  ("birth order" OR "multiple births" OR "birth rank" OR "parity" OR "family characteristics" OR "family size" OR "family structure" OR "family demography" OR "family composition" OR "household size" OR "household demography" OR "household composition" OR "siblings" OR "sibling relations" OR "sister" OR "brother" OR "sibship") AND ("exercise-induced bronchospasm" OR "asthma" OR "airway hyper-responsiveness" OR "respiratory hyper-responsiveness" OR "wheeze" OR "wheezing" OR "hypersensitivity" OR "atopic sensitization" OR "atopy" OR "allergic sensitization" OR "allergic disease" OR "allergic condition" OR "allergy" OR "allergies" OR "dermatitis" OR "eczema" OR "neurodermatitis" OR "besnier’s prurigo" OR "urticaria" OR "anaphylaxis" OR "anaphylactic shock" OR "quincke’s edema" OR "angionuerotic edema" OR "angioedema" OR "hives" OR "rhinoconjunctivitis" OR "rhinitis" OR "allergic conjunctivitis" OR "vernal keratoconjunctivitis" OR "vernal conjunctivitis" OR "giant papillary conjunctivitis" OR "hay fever" OR "pollinosis" OR "pollenosis" OR "nasal catarrh") | |

### Table S3C. Search strategy for CINAHL

| **#** | **Search term(s)** |
| --- | --- |
| 1 | (MH ’Birth Order+’) OR (MH ’Parity+’) OR ’birth order*’ OR ’birth rank*’ OR ’multiple birth*’ OR ’parity’ |
| 2 | (MH ’Family Characteristics+’) OR (MH ’Family Health+’) OR ’family charactersitic*’ OR ’family size*’ OR ’family structure*’ OR ’family demograph*’ OR ’family composition’ OR ’household size*’ OR ’household demograph*’ OR ’household composition’ |
| 3 | (MH ’Siblings+’) OR (MH ’Sibling Relations+’) OR ’sibling*’ OR ’sister*’ OR ’brother*’ OR ’sibship size*’ OR ’sibship*’ |
| 4 | (MH ’Asthma+’) OR (MH ’Respiratory Hypersensitivity+’) OR ’bronchial asthma*’ OR ’exerciseinduced asthma’ OR ’asthma*’ OR ’exercise-induced bronchospasm’ OR ’respiratory hypersensitivit*’ OR ‘airway hyper responsiveness’ OR ’airway hyper-responsiveness’ OR ’respiratory hyper responsiveness’ OR ’respiratory hyper-responsiveness’ OR ’wheez*’ |
| 5 | (MH ’Hypersensitivity+’) OR (MH ’Hypersensitivity, Immediate+’) OR (MH ’Hypersensitivity, Delayed+’) OR (MH ’Allergy and Immunology+’) OR ’immedate hypersensitivit*’ OR ’delayed hypersensitivit*’ OR ’IgE-mediated hypersensitivit*’ OR ’type I hypersensitivit*’ OR ’type IV hypersensitivit*’ OR ’hypersensitivit*’ OR ’atopic sensitiziation’ OR ’atop*’ OR ’allergic sensitization’ OR ’allergic disease* OR ’allerg*’ |
| 6 | (MH ’Dermatitis, Atopic+’) OR (MH ’Eczema+’) OR (MH ‘Angioedema+’) OR (MH ‘Anaphylaxis+’) OR (MH ’Urticaria+’) OR ’atopic dermatitis’ OR ’dermatitis’ OR ’atopic eczema’ OR ’eczema’ OR ’nerudoarmatiti*’ OR “besnier’s prurigo” OR ‘besniers prurigo’ OR ‘besnier prurigo’ OR ’urticari*’ OR ’hives’ OR ’anaphyla*’ OR ”quincke’s edema” OR ’quinckes edema’ OR ’quincke edema’ OR ’angioneurotic edema’ OR ’angioedema’ |
| 7 | (MH ’Food Hypersensitivity+’) OR ’food hypersensitivit*’ OR ’food allerg*’ OR ’egg hypersensitivit*’ OR ’egg allerg*’ OR ’milk hypersensitivit*’ OR ’milk allerg*’ OR ’shellfish hypersensitivit*’ OR ’shellfish allerg*’ OR ’wheat hypersensitivit*’ OR ’wheat allerg*’ OR ’nut hypersensitivit*’ OR ’nut allerg*’ OR ’peanut hypersensitivit*’ OR ’peanut allerg*’ OR ’groundnut hypersensitivit*’ OR ’groundnut allerg*’ |
| 8 | (MH ’Rhinitis, Allergic+’) OR (MH ’Rhinitis, Allergic, Seasonal+’) OR (MH ’Rhinitis, Allergic, Perennial+’) OR (MH ’Rhinitis+’) OR (MH ’Conjunctivitis, Allergic+’) OR (MH ’Conjunctivitis+’) OR ’allergic rhinoconjunctiviti*’ OR ’rhinoconjunctiviti*’ OR ’allergic rhiniti*’ OR ’seasonal allergic rhiniti*’ OR ’perennial allergic rhiniti*’ OR ’rhiniti*’ OR ’allergic conjunctiviti*’ OR ’vernal keratoconjunctiviti*’ OR ’vernal conjunctiviti*’ OR ’giant papillary conjunctiviti*’ OR ’hay fever’ OR ’hayfever’ OR ’pollinosis’ OR ’pollenosis’ OR ’nasal catarrh’ |
| 9 | 1 OR 2 OR 3 |
| 10 | 4 OR 5 OR 6 OR 7 OR 8 |
| 11 | 9 AND 10 |
| **Full query**  ((MH "Birth Order+") OR (MH "Parity+") OR "birth order*" OR "birth rank*" OR "multiple birth*" OR "parity" OR (MH "Family Characteristics+") OR (MH "Family Health+") OR "family charactersitic*" OR "family size*" OR "family structure*" OR "family demograph*" OR "family composition" OR "household size*" OR "household demograph*" OR "household composition" OR (MH "Siblings+") OR (MH "Sibling Relations+") OR "sibling*" OR "sister*" OR "brother*" OR "sibship size*" OR "sibship*") AND ((MH "Asthma+") OR (MH "Respiratory Hypersensitivity+") OR "bronchial asthma*" OR "exercise-induced asthma" OR "asthma*" OR "exercise-induced bronchospasm" OR "respiratory hypersensitivit*" OR ‘airway hyper responsiveness" OR "airway hyper-responsiveness" OR "respiratory hyper responsiveness" OR "respiratory hyper-responsiveness" OR "wheez*" OR (MH "Hypersensitivity+") OR (MH "Hypersensitivity, Immediate+") OR (MH "Hypersensitivity, Delayed+") OR (MH "Allergy and Immunology+") OR "immedate hypersensitivit*" OR "delayed hypersensitivit*" OR "IgE-mediated hypersensitivit*" OR "type I hypersensitivit*" OR "type IV hypersensitivit*" OR "hypersensitivit*" OR "atopic sensitiziation" OR "atop*" OR "allergic sensitization" OR "allergic disease* OR "allerg*" OR (MH "Dermatitis, Atopic+") OR (MH "Eczema+") OR (MH ‘Angioedema+") OR (MH ‘Anaphylaxis+") OR (MH "Urticaria+") OR "atopic dermatitis" OR "dermatitis" OR "atopic eczema" OR "eczema" OR "nerudoarmatiti*" OR "besnier's prurigo" OR "besniers prurigo" OR "besnier prurigo" OR "urticari*" OR "hives" OR "anaphyla*" OR "quincke's edema" OR "quinckes edema" OR "quincke edema" OR "angioneurotic edema" OR "angioedema" OR (MH "Food Hypersensitivity+") OR "food hypersensitivit*" OR "food allerg*" OR "egg hypersensitivit*" OR "egg allerg*" OR "milk hypersensitivit*" OR "milk allerg*" OR "shellfish hypersensitivit*" OR "shellfish allerg*" OR "wheat hypersensitivit*" OR "wheat allerg*" OR "nut hypersensitivit*" OR "nut allerg*" OR "peanut hypersensitivit*" OR "peanut allerg*" OR "groundnut hypersensitivit*" OR "groundnut allerg*" OR (MH "Rhinitis, Allergic+") OR (MH "Rhinitis, Allergic, Seasonal+") OR (MH "Rhinitis, Allergic, Perennial+") OR (MH "Rhinitis+") OR (MH "Conjunctivitis, Allergic+") OR (MH "Conjunctivitis+") OR "allergic rhinoconjunctiviti*" OR "rhinoconjunctiviti*" OR "allergic rhiniti*" OR "seasonal allergic rhiniti*" OR "perennial allergic rhiniti*" OR "rhiniti*" OR "allergic conjunctiviti*" OR "vernal keratoconjunctiviti*" OR "vernal conjunctiviti*" OR "giant papillary conjunctiviti*" OR "hay fever" OR "hayfever" OR "pollinosis" OR "pollenosis" OR "nasal catarrh")) | |

MH = subject heading

### Table S3D. Search strategy for EMBASE

| **#** | **Search term(s)** |
| --- | --- |
| 1 | sibship.mp. |
| 2 | birth order.mp. or exp birth order/ |
| 3 | birth rank.mp. |
| 4 | multiple birth.mp. |
| 5 | parity.mp. or exp parity/ |
| 6 | family characteristic.mp. or exp family size/ |
| 7 | family structure.mp. |
| 8 | family demograph.mp. |
| 9 | family demograph*.mp. |
| 10 | family composition.mp. |
| 11 | household size.mp. |
| 12 | household demograph*.mp. |
| 13 | household composition.mp. |
| 14 | exp sibling relation/ or sibling.mp. or sibling/ |
| 15 | exp sister/ or sister.mp. |
| 16 | siblings.mp. |
| 17 | sisters.mp. |
| 18 | brother.mp. or exp brother/ |
| 19 | brothers.mp. |
| 20 | or/1-19 |
| 21 | exp Asthma/ or (bronchial asthma* or exercise-induced asthma* or exercise-induced bronchospasm* or asthma* or respiratory hypersensitivit* or airway hyper responsiveness or airway hyperresponsiveness or respiratory hyper responsiveness or respiratory hyper-responsiveness or wheez*).mp. |
| 22 | exp Hypersensitivity/ or exp Hypersensitivity Immediate/ or exp Hypersensitivity Delayed/ or (immediate hypersensitivit* or delayed hypersensitivit* or IgE-mediated hypersensitivit* or type I hypersensitivit* or type IV hypersensitivit* or atopic sensitization or atop* or allergic sensitization or allerg*).mp. |
| 23 | exp Dermatitis/ or exp Anaphylaxis/ or (atopic dermatitis or dermatitis or neurodermatiti* or besnier prurigo or besniers prurigo or besnier prurigo or atopic eczema or eczema or urticari* or anaphyla* or quincke edema or quinckes edema or quincke edema or angioneurotic edema or angioedema or hives).mp. |
| 24 | exp Food Hypersensitivity/ or (food hypersensitivit* or food allerg* or egg hypersensitivit* or egg allerg* or milk hypersensitivit* or milk allerg* or shellfish hypersensitivit* or shellfish allerg* or wheat hypersensitivit* or wheat allerg* or nut hypersensitivit* or nut allerg* or peanut hypersensitivit* or peanut allerg* or groundnut hypersensitivit* or groundnut allerg*).mp. |
| 25 | exp Rhinitis/ or exp Conjunctivitis/ or (allergic rhinoconjunctiviti* or rhinoconjunctiviti* or allergi rhiniti* or seasonal allergic rhiniti* or perennial allergic rhiniti* or rhiniti* or allergic conjunctiviti* or vernal keratoconjunctiviti* or vernal conjunctiviti* or giant papillary conjunctiviti* or hay fever or hayfever or pollinosis or pollinosis or nasal catarrh*).mp. |
| 26 | or/21-25 |
| 27 | 20 and 26 |

### Table S3E. Search strategy for Google Scholar

| **#** | **Search term(s)** |
| --- | --- |
| 1 | "family size" OR "family structure" OR "household size" |
| 2 | "sibling" OR "sibship" |
| 3 | "asthma" OR "wheezing" |
| 4 | "atopy" OR "allergy" |
| 5 | "eczema" OR "urticaria" OR "angioedema" OR "anaphylaxis" |
| 6 | "rhinitis" OR "allergic conjunctivitis" OR "hay fever" |
| 7 | 1 OR 2 |
| 8 | 3 OR 4 OR 5 OR 6 |
| 9 | 7 AND 8 |
| **Full query**  ("family size" OR "family structure" OR "household size" OR "sibling" OR "sibship") AND ("asthma" OR "wheezing" OR "atopy" OR "allergy" OR "anaphylaxis" OR "eczema" OR "urticaria" OR "angioedema" OR "rhinitis" OR "allergic conjunctivitis" OR "hay fever") | |

### Table S3F. Search strategy for PsycINFO

| **#** | **Search term(s)** |
| --- | --- |
| 1 | SU.EXACT.EXPLODE("Birth Order") OR TI,AB(”birth order*”) OR TI,AB(”multiple birth*”) OR TI,AB(”birth rank*”) OR TI,AB(”parity”) |
| 2 | SU.EXACT.EXPLODE("Family Structure") OR SU.EXACT.EXPLODE("Family Size") OR TI,AB(”family characteristic*”) OR TI,AB(”family size*”) OR TI,AB(”family structure*”) OR TI,AB(”family demograph*”) OR TI,AB(”family composition”) OR TI,AB(”household size*”) OR TI,AB(”household demograph*”) OR TI,AB(”household composition”) |
| 3 | SU.EXACT.EXPLODE("Siblings") OR SU.EXACT.EXPLODE("Sibling Relations") OR TI,AB(”sibling*’”) OR TI,AB(”sister*”) OR TI,AB(”brother*”) OR TI,AB(”sibship size*”) OR TI,AB(”sibship size*”) OR TI,AB(”sibship*”) |
| 4 | SU.EXACT.EXPLODE("Asthma") OR TI,AB(”bronchial asthma*”) OR TI,AB(”exercise-induced asthma*”) OR TI,AB(”asthma*”) OR TI,AB(”exercise-induced bronchospasm*”) OR TI,AB(”respiratory hypersensitivit*”) OR TI,AB(”airway hyper responsiveness”) OR TI,AB(”airway hyper-responsiveness”) OR TI,AB(”respiratory hyperresponsiveness”) OR TI,AB(”respiratory hyper-responsiveness”) OR TI,AB(”wheez*”) |
| 5 | SU.EXACT.EXPLODE("Allergic Disorders") OR TI,AB(”immediate hypersensitivit*”) OR TI,AB(”delayed hypersensitivit*”) OR TI,AB(”hypersensitivit*”) OR TI,AB(”IgE-mediated hypersensitivit*”) OR TI,AB(”type I hypersensitivit*”) OR TI,AB(”type IV hypersensitivit*”) OR TI,AB(”atopic sensitization”) OR TI,AB(”atop*”) OR TI,AB(”allergic sensitization”) OR TI,AB(”allergic disease*”) OR TI,AB(”allerg*”) |
| 6 | SU.EXACT.EXPLODE("Allergic Skin Disorders") OR SU.EXACT.EXPLODE("Neurodermatitis") OR SU.EXACT.EXPLODE("Dermatitis") OR SU.EXACT.EXPLODE("Eczema") OR SU.EXACT.EXPLODE("Anaphylactic Shock") OR TI,AB(”atopic dermatitis”) OR TI,AB(”dermatitis”) OR TI,AB(”atopic eczema”) OR TI,AB(”eczema”) OR TI,AB(”neurodermatiti*”) OR TI,AB(”besnier’s prurigo”) OR TI,AB(”besniers prurigo”) OR TI,AB(”besnier prurigo”) OR TI,AB(”urticari*”) OR TI,AB(”hives”) OR TI,AB(”anaphyla*”) OR TI,AB(”quincke’s edema”) OR TI,AB(”quinckes edema”) OR TI,AB(”quincke edema”) OR TI,AB(”angioneurotic edema”) OR TI,AB(”angioedema”) |
| 7 | SU.EXACT.EXPLODE("Food Allergies") OR TI,AB(”food hypersensitivit*”) OR TI,AB(”food allerg*”) OR TI,AB(”egg hypersensitivit*”) OR TI,AB(”egg allerg*”) OR TI,AB(”milk hypersensitivit*”) OR TI,AB(”milk allerg*”) OR TI,AB(”shellfish hypersensitivit*”) OR TI,AB(”shellfish allerg*”) OR TI,AB(”wheat hypersensitivit*”) OR TI,AB(”wheat allerg*”) OR  TI,AB(”nut hypersensitivit*”) OR TI,AB(”nut allerg*”) OR TI,AB(”peanut hypersensitivit*”) OR TI,AB(”peanut allerg*”) OR TI,AB(”groundnut hypersensitivit*”) OR TI,AB(”groundnut allerg*”) |
| 8 | TI,AB(”allergic rhinoconjunctiviti*”) OR TI,AB(”rhinoconjunctiviti*”) OR TI,AB(”allergic rhiniti*”) OR TI,AB(”rhiniti*”) OR TI,AB(”seasonal allergic rhiniti*”) OR TI,AB(”perennial allergic rhiniti*”) OR TI,AB(”allergic conjunctiviti*”) OR TI,AB(”vernal keratoconjunctiviti*”) OR TI,AB(”vernal conjunctiviti*”) OR TI,AB(”giant papillary conjunctiviti*”) OR TI,AB(”hay fever”) OR TI,AB(”hayfever”) OR TI,AB(“pollinosis”) OR TI,AB(”pollenosis”) OR TI,AB(”nasal catarrh*”) |
| 9 | 1 OR 2 OR 3 |
| 10 | 4 OR 5 OR 6 OR 7 OR 8 |
| 11 | 9 AND 10 |
| **Full query**  (SU.EXACT.EXPLODE("Birth Order") OR TI,AB("birth order*") OR TI,AB("multiple birth*") OR TI,AB("birth rank*") OR TI,AB("parity") OR SU.EXACT.EXPLODE("Family Structure") OR SU.EXACT.EXPLODE("Family  Size") OR TI,AB("family characteristic*") OR TI,AB("family size*") OR TI,AB("family structure*") OR TI,AB("family demograph*") OR TI,AB("family composition") OR TI,AB("household size*") OR TI,AB("household demograph*") OR TI,AB("household composition") OR SU.EXACT.EXPLODE("Siblings") OR SU.EXACT.EXPLODE("Sibling Relations") OR TI,AB("sibling*") OR TI,AB("sister*") OR TI,AB("brother*") OR TI,AB("sibship size*") OR TI,AB("sibship size*") OR TI,AB("sibship*")) AND (SU.EXACT.EXPLODE("Asthma") OR TI,AB("bronchial asthma*") OR TI,AB("exercise-induced asthma*") OR TI,AB("asthma*") OR TI,AB("exerciseinduced bronchospasm*") OR TI,AB("respiratory hypersensitivit*") OR TI,AB("airway hyper responsiveness") OR TI,AB("airway hyper-responsiveness") OR TI,AB("respiratory hyper responsiveness") OR TI,AB("respiratory hyperresponsiveness") OR TI,AB("wheez*") OR SU.EXACT.EXPLODE("Allergic Disorders") OR TI,AB("immediate hypersensitivit*") OR TI,AB("delayed hypersensitivit*") OR TI,AB("hypersensitivit*") OR TI,AB("IgE-mediated hypersensitivit*") OR TI,AB("type I hypersensitivit*") OR TI,AB("type IV hypersensitivit") OR TI,AB("atopic sensitization") OR TI,AB("atop*") OR TI,AB("allergic sensitization") OR TI,AB("allergic disease*") OR TI,AB("allerg*") OR SU.EXACT.EXPLODE("Allergic Skin Disorders") OR SU.EXACT.EXPLODE("Neurodermatitis") OR SU.EXACT.EXPLODE("Dermatitis") OR SU.EXACT.EXPLODE("Eczema") OR SU.EXACT.EXPLODE("Anaphylactic Shock") OR TI,AB("atopic dermatitis") OR TI,AB("dermatitis") OR TI,AB("atopic eczema") OR TI,AB("eczema") OR TI,AB("neurodermatiti*") OR TI,AB("besnier’s prurigo") OR TI,AB("besniers prurigo") OR TI,AB("besnier prurigo") OR TI,AB("urticari*") OR TI,AB("hives") OR TI,AB("anaphyla*") OR TI,AB("quincke’s edema") OR TI,AB("quinckes edema") OR TI,AB("quincke edema") OR TI,AB("angioneurotic edema") OR TI,AB("angioedema") OR SU.EXACT.EXPLODE("Food Allergies") OR TI,AB("food hypersensitivit*") OR TI,AB("food allerg*") OR TI,AB("egg hypersensitivit*") OR TI,AB("egg allerg*") OR TI,AB("milk hypersensitivit*") OR TI,AB("milk allerg*") OR TI,AB("shellfish hypersensitivit*") OR TI,AB("shellfish allerg*") OR TI,AB("wheat hypersensitivit*") OR TI,AB("wheat allerg*") OR TI,AB("nut hypersensitivit*") OR TI,AB("nut allerg*") OR TI,AB("peanut hypersensitivit*") OR TI,AB("peanut allerg*") OR TI,AB("groundnut hypersensitivit*") OR TI,AB("groundnut allerg*") OR TI,AB("allergic rhinoconjunctiviti*") OR TI,AB("rhinoconjunctiviti*") OR TI,AB("allergic rhiniti*") OR TI,AB("rhiniti*") OR TI,AB("seasonal allergic rhiniti*") OR TI,AB("perennial allergic rhiniti*") OR TI,AB("allergic conjunctiviti*") OR TI,AB("vernal keratoconjunctiviti*") OR TI,AB("vernal conjunctiviti*") OR TI,AB("giant papillary conjunctiviti*") OR TI,AB("hay fever") OR TI,AB("hayfever") OR TI,AB(“pollinosis") OR TI,AB("pollenosis") OR TI,AB("nasal catarrh*")) | |

SU = all subjects and indexing; TI,AB = title, abstract

### Table S3G. Search strategy for PubMed

| **#** | **Search term(s)** |
| --- | --- |
| 1 | Birth Order[mh] OR Parity[mh] OR birth order*[tiab] OR multiple birth*[tiab] OR birth rank*[tiab] OR parity[tiab] |
| 2 | Family Characteristics[mh] OR Family Health[mh] OR family characteristic*[tiab] OR family size*[tiab] OR family structure*[tiab] OR family demograph*[tiab] OR family composition[tiab] OR household size*[tiab] OR household demograph*[tiab] OR household composition[tiab] |
| 3 | Siblings[mh] OR Sibling Relations[mh] OR sibling*[tiab] OR sister*[tiab] OR brother*[tiab] OR sibship size*[tiab] OR sibship*[tiab] |
| 4 | Asthma[mh] OR Asthma, Exercise-Induced[mh] OR Respiratory Hypersensitivity[mh] OR bronchial asthma*[tiab] OR exercise-induced asthma*[tiab] OR exercise-induced bronchospasm*[tiab] OR asthma*[tiab] OR respiratory hypersensitivit*[tiab] OR airway hyper responsiveness[tiab] OR airway hyper-responsiveness[tiab] OR respiratory hyper responsiveness[tiab] OR respiratory hyperresponsiveness[tiab] OR wheez*[tiab] |
| 5 | Hypersensitivity[mh] OR Hypersensitivity, Immediate[mh] OR Hypersensitivity, Delayed[mh] OR Allergy and Immunology[mh] OR Allergens / Immunology[mh] OR immediate hypersensitivit*[tiab] OR delayed hypersensitivit*[tiab] OR hypersensitivit*[tiab] OR IgE-mediated hypersensitivit*[tiab] OR type I hypersensitivit*[tiab] OR type IV hypersensitivit*[tiab] OR atopic sensitization[tiab] OR atop*[tiab] OR allergic sensitization[tiab] OR allergic disease*[tiab] OR allerg*[tiab] |
| 6 | Dermatitis, Atopic[mh] OR Eczema[mh] OR Angioedema[mh] OR Anaphylaxis[mh] OR Urticaria[mh] OR atopic dermatitis[tiab] OR dermatitis[tiab] OR atopic eczema[tiab] OR eczema[tiab] OR neurodermatiti*[tiab] OR besnier’s prurigo[tiab] OR besniers prurigo[tiab] OR besnier prurigo[tiab] OR urticari*[tiab] OR anaphyla*[tiab] OR quincke edema[tiab] OR quinckes edema[tiab] OR quincke’s edema[tiab] OR angioneurotic edema[tiab] OR angioedema[tiab] OR hives[tiab] |
| 7 | Food Hypersensitivity[mh] OR food hypersensitivit*[tiab] OR food allerg*[tiab] OR egg allerg*[tiab] OR egg hypersensitivit*[tiab] OR milk allerg*[tiab] OR milk hypersensitivit*[tiab] OR shellfish allerg*[tiab] OR shellfish hypersensitivit*[tiab] OR wheat allerg*[tiab] OR wheat hypersensitivit*[tiab] OR nut allerg*[tiab] OR nut hypersensitivit*[tiab] OR peanut allerg*[tiab] OR peanut hypersensitivit*[tiab] OR groundnut hypersensitivit*[tiab] |
| 8 | Pollen / Immunology[mh] OR Rhinitis, Allergic[mh] OR Rhinitis, Allergic, Seasonal[mh] OR Rhinitis, Allergic, Perennial[mh] OR Rhinitis[mh] OR Conjunctivitis, Allergic[mh] OR Conjunctivitis / Immunology[mh] OR Conjunctivitis / Epidemiology[mh] OR Conjunctivitis / Etiology[mh] OR allergic rhinoconjunctiviti*[tiab] OR rhinoconjunctiviti*[tiab] OR allergic rhiniti*[tiab] OR rhiniti*[tiab] OR seasonal allergic rhiniti*[tiab] OR perennial allergic rhiniti*[tiab] OR allergic conjunctiviti*[tiab] OR vernal keratoconjunctiviti*[tiab] OR vernal conjunctiviti*[tiab] OR giant papillary conjunctiviti*[tiab] OR hay fever[tiab] OR hayfever[tiab] OR pollinosis[tiab] OR pollenosis[tiab] OR nasal catarrh*[tiab] |
| 9 | 1 OR 2 OR 3 |
| 10 | 4 OR 5 OR 6 OR 7 OR 8 |
| 11 | 9 AND 10 |
| **Full query**  (Birth Order[mh] OR Parity[mh] OR birth order*[tiab] OR multiple birth*[tiab] OR birth rank*[tiab] OR parity[tiab] OR Family Characteristics[mh] OR Family Health[mh] OR family characteristic*[tiab] OR family size*[tiab] OR family structure*[tiab] OR family demograph*[tiab] OR family composition[tiab] OR household size*[tiab] OR household demograph*[tiab] OR household composition[tiab] OR Siblings[mh] OR Sibling Relations[mh] OR sibling*[tiab] OR sister*[tiab] OR brother*[tiab] OR sibship size*[tiab] OR sibship*[tiab]) AND (Asthma[mh] OR Asthma, Exercise-Induced[mh] OR Respiratory Hypersensitivity[mh] OR bronchial asthma*[tiab] OR exercise-induced asthma*[tiab] OR exercise-induced bronchospasm*[tiab] OR asthma*[tiab] OR respiratory hypersensitivit*[tiab] OR airway hyper responsiveness[tiab] OR airway hyper-responsiveness[tiab] OR respiratory hyper responsiveness[tiab] OR respiratory hyper-responsiveness[tiab] OR wheez*[tiab] OR Hypersensitivity[mh] OR Hypersensitivity, Immediate[mh] OR Hypersensitivity, Delayed[mh] OR Allergy and Immunology[mh] OR Allergens / Immunology[mh] OR immediate hypersensitivit*[tiab] OR delayed hypersensitivit*[tiab] OR hypersensitivit*[tiab] OR IgE-mediated hypersensitivit*[tiab] OR type I hypersensitivit*[tiab] OR type IV hypersensitivit*[tiab] OR atopic sensitization[tiab] OR atop*[tiab] OR allergic sensitization[tiab] OR allergic disease*[tiab] OR allerg*[tiab] OR Dermatitis, Atopic[mh] OR Eczema[mh] OR Angioedema[mh] OR Anaphylaxis[mh] OR Urticaria[mh] OR atopic dermatitis[tiab] OR dermatitis[tiab] OR atopic eczema[tiab] OR eczema[tiab] OR neurodermatiti*[tiab]OR besnier’s prurigo[tiab] OR besniers prurigo[tiab] OR besnier prurigo[tiab] OR urticari*[tiab] OR anaphyla*[tiab] OR quincke edema[tiab] OR quinckes edema[tiab] OR quincke’s edema[tiab] OR angioneurotic edema[tiab] OR angioedema[tiab] OR hives[tiab] OR Food Hypersensitivity[mh] OR food hypersensitivit*[tiab] OR food allerg*[tiab] OR egg allerg*[tiab] OR egg hypersensitivit*[tiab] OR milk allerg*[tiab] OR milk hypersensitivit*[tiab] OR shellfish allerg*[tiab] OR shellfish hypersensitivit*[tiab] OR wheat allerg*[tiab] OR wheat hypersensitivit*[tiab] OR nut allerg*[tiab] OR nut hypersensitivit*[tiab] OR peanut allerg*[tiab] OR peanut hypersensitivit*[tiab] OR groundnut hypersensitivit*[tiab] OR Pollen / Immunology[mh] OR Rhinitis, Allergic[mh] OR Rhinitis, Allergic, Seasonal[mh] OR Rhinitis, Allergic, Perennial[mh] OR Rhinitis[mh] OR Conjunctivitis, Allergic[mh] OR Conjunctivitis / Immunology[mh] OR Conjunctivitis / Epidemiology[mh] OR Conjunctivitis / Etiology[mh] OR allergic rhinoconjunctiviti*[tiab] OR rhinoconjunctiviti*[tiab] OR allergic rhiniti*[tiab] OR rhiniti*[tiab] OR seasonal allergic rhiniti*[tiab] OR perennial allergic rhiniti*[tiab] OR allergic conjunctiviti*[tiab] OR vernal keratoconjunctivitis*[tiab] OR vernal conjunctiviti*[tiab] OR giant papillary conjunctiviti*[tiab] OR hay fever[tiab] OR hayfever[tiab] OR pollinosis[tiab] OR pollenosis[tiab] OR nasal catarrh*[tiab]) | |

mh = MeSH; tiab = title, abstract

### Table S3H. Search strategy for Scopus

| **#** | **Search term(s)** |
| --- | --- |
| 1 | TITLE-ABS-KEY(”birth order*”) OR TITLE-ABS-KEY(”multiple birth*”) OR TITLE-ABS-KEY(”birth rank*”) OR TITLE-ABS-KEY(”parity”) |
| 2 | TITLE-ABS-KEY(”family characteristic*”) OR TITLE-ABS-KEY(”family size*”) OR TITLEABS-KEY(”family structure*”) OR TITLE-ABS-KEY(”family demograph*”) OR TITLE-ABS-KEY(”family composition”) OR TITLE-ABS-KEY(”household size*”) OR TITLE-ABS-KEY(”household demograph*”) OR TITLE-ABS-KEY(”household composition*”) |
| 3 | TITLE-ABS-KEY(”sibling relation*”) OR TITLE-ABS-KEY(”sibling*”) OR TITLE-ABSKEY(”brother*”) OR TITLE-ABS-KEY(”sister*”) OR TITLE-ABS-KEY(”sibship size*”) OR TITLE-ABS-KEY(”sibship*”) |
| 4 | TITLE-ABS-KEY(”bronchial asthma*”) OR TITLE-ABS-KEY(”exercise-induced asthma*”) OR TITLE-ABS-KEY(”asthma*”) OR TITLE-ABS-KEY(”exercise-induced bronchospasm*”) OR TITLE-ABS-KEY(”respiratory hypersensitivit*”) OR TITLE-ABS-KEY(”airway hyper responsiveness”) OR TITLE-ABS-KEY(”airway hyper-responsiveness”) OR TITLE-ABSKEY(”respiratory hyper responsiveness”) OR TITLE-ABS-KEY(”respiratory hyperresponsiveness”) OR TITLE-ABS-KEY(”wheez*”) |
| 5 | TITLE-ABS-KEY(”immediate hypersensitivit*”) OR TITLE-ABS-KEY(”delayed hypersensitivit*”) OR TITLE-ABS-KEY(”IgE-mediated hypersensitivit*”) OR TITLE-ABS-KEY(”type I hypersensitivit*”) OR TITLE-ABS-KEY(”type IV hypersensitivit*”) OR TITLE-ABS-KEY(”atopic sensitization”) OR TITLE-ABS-KEY(”atop*”) OR TITLE-ABS-KEY(”allergic sensitization”) OR TITLE-ABS-KEY(”allergic disease”) OR TITLE-ABS-KEY(”allerg*”) |
| 6 | TITLE-ABS-KEY(”atopic dermatitis”) OR TITLE-ABS-KEY(”dermatitis”) OR TITLE-ABS-KEY(”atopic eczema”) OR TITLE-ABS-KEY(”eczema”) OR TITLE-ABS-KEY(”neurodermatitis*”) OR TITLE-ABS-KEY(”besnier’s prurigo”) OR TITLE-ABS-KEY(”besniers prurigo”) OR TITLE-ABS-KEY(”besnier prurigo”) OR TITLE-ABSKEY(”quincke’s edema”) OR TITLE-ABS-KEY(”quinckes edema”) OR TITLE-ABSKEY(”quincke edema”) OR TITLE-ABS-KEY(”angioneurotic edema”) OR TITLE-ABS-KEY(”hives”) OR TITLE-ABS-KEY(”anaphyla*”) OR TITLE-ABS-KEY(”urticari*”) |
| 7 | TITLE-ABS-KEY(”food hypersensitivit*”) OR TITLE-ABS-KEY(”food allerg*”) OR TITLE-ABSKEY(”egg allerg*”) OR TITLE-ABS-KEY(”egg hypersensitivit*”) OR TITLE-ABS-KEY(”milk allerg*”) OR TITLE-ABS-KEY(”milk hypersensitivit*”) OR TITLE-ABS-KEY(”shellfish allerg*”) OR TITLE-ABS-KEY(”shellfish hypersensitivit*”) OR TITLE-ABS-KEY(”wheat allerg*”) OR TITLE-ABS-KEY(”wheat hypersensitivit*”) OR TITLE-ABS-KEY(”nut allerg*”) OR TITLE-ABS-KEY(”nut hypersensitivit*”) OR TITLE-ABS-KEY(”peanut allerg*”) OR TITLE-ABS-KEY(”peanut hypersensitivit*”) OR TITLE-ABS-KEY(”groundnut allerg*”) OR TITLE-ABS-KEY(”groundnut hypersensitivit*”) |
| 8 | TITLE-ABS-KEY(”allergic rhinoconjunctiviti*”) OR TITLE-ABS-KEY(”rhinoconjunctiviti*”) OR TITLE-ABS-KEY(”seasonal allergic rhiniti*”) OR TITLE-ABS-KEY(”perennial allergic rhiniti*”) OR TITLE-ABS-KEY(”allergic rhiniti*”) OR TITLE-ABS-KEY(”rhiniti*”) OR TITLE-ABSKEY(”allergic conjunctiviti*”) OR TITLE-ABS-KEY(”vernal keratoconjunctiviti*”) OR TITLE-ABS-KEY(”vernal conjunctiviti*”) OR TITLE-ABS-KEY(”giant papillary conjunctiviti*”) OR TITLE-ABS-KEY(”hay fever”) OR TITLE-ABS-KEY(”hayfever”) OR TITLE-ABS-KEY(”pollinosis”) OR TITLE-ABS-KEY(”pollenosis”) OR TITLE-ABS-KEY(”nasal catarrh*”) |
| 9 | 1 OR 2 OR 3 |
| 10 | 4 OR 5 OR 6 OR 7 OR 8 |
| 11 | 9 AND 10 |
| **Full query**  (TITLE-ABS-KEY(”birth order*”) OR TITLE-ABS-KEY(”multiple birth*”) OR TITLE-ABS-KEY(”birth rank*”) OR TITLE-ABS-KEY(”parity”) OR TITLE-ABS-KEY(”family characteristic*”) OR TITLE-ABS-KEY(”family size*”) OR TITLE-ABS-KEY(”family structure*”) OR TITLE-ABS-KEY(”family demograph*”) OR TITLE-ABS-KEY(”family composition”) OR TITLE-ABS-KEY(”household size*”) OR TITLE-ABS-KEY(”household demograph*”) OR TITLE-  ABS-KEY(”household composition*”) OR TITLE-ABS-KEY(”sibling relation*”) OR TITLE-ABS-KEY(”sibling*”) OR TITLE-ABS-KEY(”brother*”) OR TITLE-ABS-KEY(”sister*”) OR TITLE-ABS-KEY(”sibship size*”) OR TITLE-ABS-KEY(”sibship*”)) AND (TITLE-ABS-KEY(”bronchial asthma*”) OR TITLE-ABS-KEY(”exerciseinduced asthma*”) OR TITLE-ABS-KEY(”asthma*”) OR TITLE-ABS-KEY(”exercise-induced bronchospasm*”) OR TITLE-ABS-KEY(”respiratory hypersensitivit*”) OR TITLE-ABS-KEY(”airway hyper responsiveness”) OR TITLE-ABS-KEY(”airway hyper-responsiveness”) OR TITLE-ABS-KEY(”respiratory hyper responsiveness”) OR TITLEABS-KEY(”respiratory hyper-responsiveness”) OR TITLE-ABS-KEY(”wheez*”) OR TITLE-ABS-KEY(”immediate hypersensitivit*”) OR TITLE-ABS-KEY(”delayed hypersensitivit*”) OR TITLE-ABS-KEY(”IgE-mediated hypersensitivit*”) OR TITLE-ABS-KEY(”type I hypersensitivit*”) OR TITLE-ABS-KEY(”type IV hypersensitivit*”) OR TITLE-ABS-KEY(”atopic sensitization”) OR TITLE-ABS-KEY(”atop*”) OR TITLE-ABS-KEY(”allergic sensitization”) OR TITLE-ABS-KEY(”allergic disease”) OR TITLE-ABS-KEY(”allerg*”) OR TITLE-ABSKEY(”atopic dermatitis”) OR TITLE-ABS-KEY(”dermatitis”) OR TITLE-ABS-KEY(”atopic eczema”) OR TITLEABS-KEY(”eczema”) OR TITLE-ABS-KEY(”neurodermatitis*”) OR TITLE-ABS-KEY(”besnier’s prurigo”) OR TITLE-ABS-KEY(”besniers prurigo”) OR TITLE-ABS-KEY(”besnier prurigo”) OR TITLE-ABS-KEY(”quincke’s edema”) OR TITLE-ABS-KEY(”quinckes edema”) OR TITLE-ABS-KEY(”quincke edema”) OR TITLE-ABSKEY(”angioneurotic edema”) OR TITLE-ABS-KEY(”hives”) OR TITLE-ABS-KEY(”anaphyla*”) OR TITLE-ABS-KEY(”urticari*”) OR TITLE-ABS-KEY(”food hypersensitivit*”) OR TITLE-ABS-KEY(”food allerg*”) OR TITLE-ABS-KEY(”egg allerg*”) OR TITLE-ABS-KEY(”egg hypersensitivit*”) OR TITLE-ABS-KEY(”milk allerg*”) OR TITLE-ABS-KEY(”milk hypersensitivit*”) OR TITLE-ABS-KEY(”shellfish allerg*”) OR TITLE-ABS-KEY(”shellfish hypersensitivit*”) OR TITLE-ABS-KEY(”wheat allerg*”) OR TITLE-ABS-KEY(”wheat hypersensitivit*”) OR TITLE-ABS-KEY(”nut allerg*”) OR TITLE-ABS-KEY(”nut hypersensitivit*”) OR TITLE-ABS-KEY(”peanut allerg*”) OR TITLE-ABS-KEY(”peanut hypersensitivit*”) OR TITLE-ABS-KEY(”groundnut allerg*”) OR TITLE-ABS-KEY(”groundnut hypersensitivit*”) OR TITLE-ABS-KEY(”allergic rhinoconjunctiviti*”) OR TITLE-ABSKEY(”rhinoconjunctiviti*”) OR TITLE-ABS-KEY(”seasonal allergic rhiniti*”) OR TITLE-ABS-KEY(”perennial allergic rhiniti*”) OR TITLE-ABS-KEY(”allergic rhiniti*”) OR TITLE-ABS-KEY(”rhiniti*”) OR TITLE-ABSKEY(”allergic conjunctiviti*”) OR TITLE-ABS-KEY(”vernal keratoconjunctiviti*”) OR TITLE-ABS-KEY(”vernal conjunctiviti*”) OR TITLE-ABS-KEY(”giant papillary conjunctiviti*”) OR TITLE-ABS-KEY(”hay fever”) OR TITLE-ABS-KEY(”hayfever”) OR TITLE-ABS-KEY(”pollinosis”) OR TITLE-ABS-KEY(”pollenosis”) OR TITLE-ABS-KEY(”nasal catarrh*”)) | |

TITLE-ABS-KEY = title, abstract, keywords

### Table S3I. Search strategy for Web of Science

| **#** | **Search term(s)** |
| --- | --- |
| 1 | TS=”birth order*” OR TS=”multiple birth*” OR TS=”birth rank*” OR TS=”parity” |
| 2 | TS=”family characteristic*” OR TS=”family size*” OR TS=”family structure*” OR TS=”family demograph*” OR TS=”family composition” OR TS=”household size*” OR TS=”household demograph*” OR TS=”household composition” |
| 3 | TS=”sibling*” OR TS=”sister*” OR TS=”brother*” OR TS=”sibship size*” OR TS=”sibship*” |
| 4 | TS=”bronchial asthma*” OR TS=”exercise-induced asthma*” OR TS=”exercise-induced bronchospasm*” OR TS=”asthma*” OR TS=“respiratory hypersensitivit*” OR TS=“respiratory hyper-responsiveness*” OR TS=“airway hyper responsiveness*” OR TS=“airway hyperresponsiveness*” OR TS=wheez* |
| 5 | TS=”immediate hypersensitivit*” OR TS=”delayed hypersensitivit*” OR TS=”IgE-mediated hypersensitivit*” OR TS=”type I hypersensitivit*” OR TS=”type IV hypersensitivit*” OR TS=”hypersensitivit*” OR TS=”atopic sensitization” OR TS=”atop*” OR TS=”allergic sensitization” OR TS=”allergic disease*” OR TS=”allerg*” |
| 6 | TS=”atopic dermatitis” OR TS=”dermatitis” OR TS=”atopic eczema” OR TS=”eczema” OR TS=”neurodermatiti*” OR TS=”besnier’s prurigo” OR TS=”besniers prurigo” OR TS=”besnier prurigo” OR TS=”urticari*” OR TS=”anaphyla*” OR TS=”quincke’s edema” OR TS=”quinckes edema” OR TS=”quincke edema” OR TS=”angioneurotic edema” OR TS=”angioedema” OR TS=”hives” |
| 7 | TS=”food hypersensitivit*” OR TS=”food allerg*” OR TS=”egg allerg*” OR TS=”egg hypersensitivit*” OR TS=”milk allerg*” OR TS=”milk hypersensitivit*” OR TS=”shellfish allerg*” OR TS=”shellfish hypersensitivit*” OR TS=”wheat allerg*” OR TS=”wheat hypersensitivit*” OR TS=”nut allerg*” OR TS=”nut hypersensitivit*” OR TS=”peanut allerg*” OR TS=”peanut hypersensitivit*” OR TS=”groundnut allerg*” OR TS=”groundnut hypersensitivit*” |
| 8 | TS=”pollen allerg*” OR TS=”allergic rhinoconjunctiviti*” OR TS=”rhinoconjunctiviti*” OR TS=”seasonal allergic rhiniti*” OR TS=”perennial allergic rhiniti*” OR TS=”allergic rhiniti*” OR TS=”rhiniti*” OR TS=”allergic conjunctiviti*” OR TS=”vernal keratoconjunctiviti*” OR TS=”vernal conjunctiviti*” OR TS=”giant papillary conjunctiviti*” OR TS=”hay fever” OR TS=”hayfever” OR TS=”pollinosis” OR TS=”pollenosis” OR TS=”nasal catarrh*” |
| 9 | 1 OR 2 OR 3 |
| 10 | 4 OR 5 OR 6 OR 7 OR 8 |
| 11 | 9 AND 10 |
| **Full query**  (TS="birth order*" OR TS="multiple birth*" OR TS="birth rank*" OR TS="parity" OR TS="family characteristic*" OR TS="family size*" OR TS="family structure*" OR TS="family demograph*" OR TS="family composition" OR TS="household size*" OR TS="household demograph*" OR TS="household composition" OR TS="sibling*" OR TS="sister*" OR TS="brother*" OR TS="sibship size*" OR TS="sibship*") AND (TS="bronchial asthma*" OR TS="exercise-induced asthma*" OR TS="exercise-induced bronchospasm*" OR TS="asthma*" OR TS=“respiratory hypersensitivit*" OR TS=“respiratory hyper-responsiveness*" OR TS=“airway hyper responsiveness*" OR TS=“airway hyper-responsiveness*" OR TS=wheez* OR TS="immediate hypersensitivit*" OR TS="delayed hypersensitivit*" OR TS="IgE-mediated hypersensitivit*" OR TS="type I hypersensitivit*" OR TS="type IV hypersensitivit*" OR TS="hypersensitivit*" OR TS="atopic sensitization" OR TS="atop*" OR TS="allergic sensitization" OR TS="allergic disease*" OR TS="allerg*" OR TS="atopic dermatitis" OR TS="dermatitis" OR TS="atopic eczema" OR TS="eczema" OR TS="neurodermatiti*" OR TS="besnier’s prurigo" OR TS="besniers prurigo" OR TS="besnier prurigo" OR TS="urticari*" OR TS="anaphyla*" OR TS="quincke’s edema" OR TS="quinckes edema" OR TS="quincke edema" OR TS="angioneurotic edema" OR TS="angioedema" OR TS="hives" OR TS="food hypersensitivit*" OR TS="food allerg*" OR TS="egg allerg*" OR TS="egg hypersensitivit*" OR TS="milk allerg*" OR TS="milk hypersensitivit*" OR TS="shellfish allerg*" OR TS="shellfish hypersensitivit*" OR TS="wheat allerg*" OR TS="wheat hypersensitivit*" OR TS="nut allerg*" OR TS="nut hypersensitivit*" OR TS="peanut allerg*" OR TS="peanut hypersensitivit*" OR TS="groundnut allerg*" OR TS="groundnut hypersensitivit*" OR TS="pollen allerg*" OR TS="allergic rhinoconjunctiviti*" OR TS="rhinoconjunctiviti*" OR TS="seasonal allergic rhiniti*" OR TS="perennial allergic rhiniti*" OR TS="allergic rhiniti*" OR TS="rhiniti*" OR TS="allergic conjunctiviti*" OR TS="vernal keratoconjunctiviti*" OR TS="vernal conjunctiviti*" OR TS="giant papillary conjunctiviti*" OR TS="hay fever" OR TS="hayfever" OR TS="pollinosis" OR TS="pollenosis" OR TS="nasal catarrh*") | |

TS = title, abstract, author keywords, Keywords Plus

## Tables of characteristics

### Table S4A. Table of characteristics for asthma

| **Study, country** | **Study design** | **Years of data collection** | **Source of study population** | **Subjects analyzed^a^** | **Age (in years)^b^** | **Exposure and assessment method** | **Outcome assessment method** | **Main findings^c^** |
| --- | --- | --- | --- | --- | --- | --- | --- | --- |
| **Aaron 1967 [1]**, US | Case-control | N/A | **Asthma group**  Clinician diagnosis of asthma, no other ailments  **Allergy group**  Clinician diagnosis of major allergy, no other ailments  **Controls**  General population | 60 | 8-11 | *Birth order*  Questionnaire | Clinician diagnosis | **Clinician diagnosis of asthma (current)**  ↑ (N/A) |
| **Addo-Yobo 2001 [2]**, GH | Case-control | N/A | **Cases**  Clinician diagnosis of asthma  **Controls**  General population | 100 | 9-16 | *Birth order*  Questionnaire | Questionnaire | **Clinician diagnosis of asthma, use of asthma medication, and presence of asthma symptoms (in last year)**  ↓ (≥ 5 vs < 5) |
| **Akcay 2014 [4]**, TR | Cross-sectional | 2004-2005 | General population | 9,991 | 13-14 | *Sibship size*  Questionnaire | Questionnaire | **Clinician diagnosis of asthma (ever)**  ↓ (≥ 3 vs 1-2) |
| **Almqvist 2016 [5]**, SE | Cohort | 2008 | General population | 314,477 | 6-12 | *Birth order*  Register data | Register data | **Clinician diagnosis of asthma or ≥ 2 asthma medication dispensings (in last year)**  ↓ (2 vs 1) |
| **Attena 1999 [6]**, IT | Cross-sectional | 1995 | Male potential conscripts | 3,065 | ~18 | *Birth order*  Questionnaire | Clinician diagnosis | **Clinician diagnosis of asthma (current)**  ↓ (≥ 2 vs 1) |
| **Ayatollahi 2006 [7]**, IR | Case-control | N/A | **Cases**  Clinician diagnosis of asthma  **Controls**  General population | 2,228 | 6-12 | *Birth order*  Questionnaire | Clinician diagnosis | **Clinician diagnosis of asthma (current)**  NS (N/A) |
| **Bager 2003 [9]**, DK | Cohort | 1997-2001 | Pregnant women | 9,722 | 20-28 | *Birth order*  Register data | Questionnaire | **Clinician diagnosis of asthma (ever)**  NS (≥ 2 vs 1)  **Clinician diagnosis (ever) and current symptoms**  NS (≥ 2 vs 1) |
| **Ball 2000 [11]**, US | Cohort | 1980-1997 | General population | 1,035 | ~13 | *Birth order*  Questionnaire | Questionnaire | **Clinician diagnosis of asthma (at age 6-13 years)**  NS (≥ 2 vs 1) |
| **Batllés-Garrido 2010 [12]**, ES | Cross-sectional | N/A | General population | 1,143 | 10-11 | *Birth order*  Questionnaire | Questionnaire | **Asthma (ever)**  NS (N/A) |
| **Bedolla-Barajas 2018 [13]**, MX | Cross-sectional | 2012-2013 | General population | 1,003 | 6-7 | *Sibship size*  Questionnaire | Questionnaire | **Asthma (current)**  NS (N/A) |
| **Bernsen 2003 [15]**, NL | Cohort | N/A | General population | 1,727 | ~6 | *Birth order, sibship size*  Medical records | Medical records and clinician consultation | **Clinician diagnosis of asthma (current)**  NS (≥ 2 vs 1) |
| **Bodner 1998 [16]**, GB | Cross-sectional | 1964 | General population | 2,111 | 10-14 | *Birth order, sibship size*  Questionnaire | Clinician diagnosis | **Clinician diagnosis of asthma (current)**  NS (≥ 2 vs 1) |
| **Bodner 2000 [17]**, GB | Nested case-control | 1995 | **Cases**  Self-reported adult-onset asthma  **Controls**  General population | 319 | 39-45 | *Birth order, sibship size*  Questionnaire | Questionnaire | **Clinician diagnosis of asthma (onset-age ≥ 15 years)**  NS (≥ 2 vs 1) |
| **Boneberger 2011 [18]**, CL | Case-control | 2008-2009 | **Cases**  Clinician diagnosis of asthma  **Controls**  Accidental injury or routine visit to healthcare institution | 482 | 6-15 | *Birth order*  Questionnaire | Clinician diagnosis | **Clinician diagnosis of asthma (current)**  NS (≥ 2 vs 1) |
| **Bråbäck 1998 [19]**, SE | Cohort | 1990-1995 | Male potential conscripts | 149,398 | 17-20 | *Birth order*  Register data | Clinician diagnosis | **Clinician diagnosis of asthma (current)**  ↑ (2-4 vs 1)  NS (≥ 5 vs 1)  **Clinician diagnosis of asthma and allergic rhinitis (current)**  ↑ (≥ 2 vs 1)  **Clinician diagnosis of asthma, no allergic rhinitis (current)**  NS (≥ 2 vs 1) |
| **Chunmei 2014 [23]**, CN | Case-control | 2010-2013 | **Cases**  Clinician diagnosis of asthma  **Controls**  General population | 375 | 1-14 | *Birth order*  Medical records | Clinician diagnosis | **Clinician diagnosis of asthma (current)**  NS (≥ 2 vs 1) |
| **De Meer 2005 [29]**, NL | Cross-sectional | 1997-1998 | General population | 1,555 | 8-13 | *Birth order*  Questionnaire | Questionnaire | **Clinician diagnosis of asthma and ≥ 1 episode of wheezing or asthma medication usage in last year**  NS (≥ 2 vs 1) |
| **Dik 2004 [30]**, CA | Cohort | 1980-1996 | General population | 170,960 | ~6 | *Sibship size*  Register data | Medical records of clinician diagnosis | **Clinician diagnosis of asthma (ever)**  ↓ (≥ 2 vs 1) |
| **Foliaki 2008 [33]**, FJ, NC, PF, TK, WS | Cross-sectional | 2002-2003 | General population | 17,683 | 6-14 | *Birth order*  Questionnaire | Questionnaire | **Asthma (ever)**  ↓ (≥ 3 vs 1) |
| **Furuhata 2020 [35]**, JP | Cohort | 2001-2010 | General population | 34,138 | ~10 | *Sibship size*  Questionnaire | Questionnaire | **Asthma-related hospital or clinic visit (at age 9-10 years)**  ↑ (≥ 2 vs 1)  **Asthma-related hospital or clinic visit (at age 6-10 years)**  ↑ (≥ 2 vs 1) |
| **Gao 2005 [36]**, CA | Cohort | 1994-1999 | General population | 1,223 | ≤ 11 | *Birth order, sibship size*  Questionnaire | Questionnaire | **Incidence of asthma (unspecified time frame)**  NS (≥ 2 vs 1) |
| **Goldberg 2007 [38]**, IL | Cross-sectional | 1998-2004 | Potential conscripts | 531,116 | ~17 | *Birth order, sibship size*  Questionnaire | Interview, medical records, and clinician diagnosis | **Clinician diagnosis of asthma (ever)**  *Birth order*  NS (≥ 2 vs 1)  *Sibship size*  ↓ (≥ 4 vs 1) |
| **Gupta 2016 [43]**, US | Cross-sectional | 2005-2011 | General population | 1,359 | ≤ 20 | *Sibship size*  Questionnaire | Questionnaire | **Clinician diagnosis of asthma (ever)**  ↓ (no. of siblings) |
| **Haby 2001 [45]**, AU | Cross-sectional | 1995 | General population | 974 | 3-5 | *Birth order*  Questionnaire | Questionnaire | **Clinician diagnosis of asthma and symptoms (cough or wheezing) and asthma medication use (in last year)**  ↓ (≥ 4 vs 1) |
| **Hedman 2015 [47]**, SE | Cohort | 1996-2007 | General population | 2,754 | ~19 | *Sibship size*  Questionnaire | Questionnaire | **Clinician diagnosis of asthma (at age 12-19 years)**  **All**  ↓ (no. of siblings)  **Atopic (positive SPT)**  NS (no. of siblings)  **Non-atopic**  ↓ (no. of siblings)  **Clinician diagnosis of asthma at age 12-19 years and current wheeze or asthma medication use (in last year)**  **All**  ↓ (no. of siblings)  **Atopic (positive SPT)**  NS (no. of siblings)  **Non-atopic**  ↓ (no. of siblings) |
| **Held 2000 [48]**, US | Cohort | 1988-1996 | General population | 131 | 4-7 | *Birth order*  Questionnaire | Interview and medical records | **Clinician diagnosis of asthma (current)**  NS (≥ 2 vs 1) |
| **Hessel 2001 [50]**, CA | Nested case-control | N/A | **Cases**  Clinician diagnosis of asthma  **Controls**  General population | 1,035 | 5-19 | *Sibship size*  Questionnaire | Questionnaire | **Clinician diagnosis of asthma and asthma attack or asthma medication use in last year**  ↓ (≥ 5 vs 1) |
| **Hossain 2020 [54]**, BD | Case-control | 2018 | **Cases**  Pediatric ward patient, clinician diagnosis of asthma  **Controls**  Pediatric ward patient, no asthma | 350 | 5-15 | *Birth order*  Questionnaire | Clinician diagnosis | **Clinician diagnosis of asthma (current)**  ↓ (3-5 vs 1-2) |
| **Huang 2018 [55]**, TW | Cohort | 2005-2013 | Children with clinician diagnosis of early-onset atopic dermatitis | 1,549 | ~8 | *Birth order*  Interview | Interview | **Clinician diagnosis of asthma (ever)**  ↓ (≥ 2 vs 1) |
| **Hughes 2013 [56]**, AU | Case-control | 2003-2006 | **Cases**  First clinical diagnosis of central nervous system demyelination  **Controls**  General population | 818 | **Cases**  18-59  **Controls**  18-61 | *Birth order, sibship size*  Interview | Interview | **Clinician diagnosis of asthma (ever)**  **Control** **group**  *Birth order*  NS (2-3 vs 1)  ↓ (≥ 2 vs 1)  *Sibship size*  ↓ (no. of siblings) |
| **Infante-Rivard 2009 [57]**, CA | Cohort | 1988-1995 | **Cases**  Clinician diagnosis of asthma  **Controls**  General population | 914 | 9-10 | *Sibship size*  Questionnaire | Clinician diagnosis | **Clinician diagnosis of asthma at age 3-4 years**  ↓ (≥ 2 vs 1)  **Clinician diagnosis of asthma at age 3-4 years and persistent symptoms or asthma medication use during 6-year follow-up**  ↓ (2 vs 1)  NS (≥ 3 vs 1)  **Clinician diagnosis of asthma at age 3-4 years and no symptoms or asthma medication use during 6-year follow-up**  NS (≥ 2 vs 1) |
| **Kansen 2020 [61]**, NL | Cross-sectional | 2011-2019 | Investigated for allergy/respiratory symptoms at secondary/tertiary care centre | 5,517 | 1-18 | *Sibship size*  Questionnaire | Questionnaire | **Asthma (ever) and ≥ 1 episode of wheezing (in last year)**  NS (≥ 2 vs 1) |
| **Kaplan 1989 [62]**, GB | Cohort | 1958-1965 | General population | 15,398 | ~7 | *Birth order*  Interview, questionnaire | Interview | **Asthma (ever)**  ↑ (≥ 3 vs 1-2) |
| **Karunanayake 2020 [64]**, CA | Cross-sectional | 2012 | Aboriginal population | 24,803 | 12-19 | *Sibship size*  Questionnaire | Questionnaire | **Current asthma diagnosed by a healthcare professional**  ↑ (2-3 vs 1)  NS (≥ 4 vs 1) |
| **Kiechl-Kohlendorfer 2007 [66]**, AT | Cohort | 1994-2005 | General population | 33,808 | 6-10 | *Sibship size*  Questionnaire | Pediatric pneumonologist review of medical records | **Hospitalization due to atopic asthma (during age 6-10 years)**  ↓ (3-4 vs 1-2)  NS (≥ 5 vs 1) |
| **Kikkawa 2018 [67]**, JP | Cohort | 2001-2014 | General population | 32,065 | ~12 | *Birth order*  Questionnaire | Questionnaire | **≥ 1 clinician visit due to asthma (in last year at age 0.5-3.5 years)**  ↑ (≥ 2 vs 1)  **≥ 1 clinician visit due to asthma (in last year at age 3.5-8 years)**  NS (≥ 2 vs 1)  **≥ 1 clinician visit due to asthma (in last year at age 8-11 years)**  NS (2 vs 1)  ↓ (≥ 3 vs 1)  **≥ 1 clinician visit due to asthma (in last year at age 11-12 years)**  NS (≥ 2 vs 1) |
| **Kilpeläinen 2000 [68]**, FI | Cross-sectional | 1995-1996 | General population | 10,667 | 18-24 | *Birth order*  Questionnaire | Questionnaire | **Clinician diagnosis of asthma (ever)**  NS (≥ 2 vs 1) |
| **Kim 2012 [69]**, KR | Cross-sectional | 2010 | Rural population | 947 | 9.6 ± N/A (primary school age) | *Sibship size*  Questionnaire | Questionnaire | **Clinician diagnosis of asthma (ever)**  NS (≥ 2 vs 1) |
| **Kingsley 1949 [70]**, US | Cohort | N/A | General population | 101 | ~5 | *Sibship size*  Interview | Clinician diagnosis | **Clinician diagnosis of asthma (current)**  ↓ (≥ 2 vs 1) |
| **Kinra 2006 [71]**, GB | Cross-sectional | 1948–1968 | General population | 14,140 | 16-30 | *Birth order, sibship size*  Questionnaire | Questionnaire | **Asthma (ever)**  *Birth order*  NS (2 vs 1)  ↓ (≥ 3 vs 1)  *Sibship size*  NS (≥ 2 vs 1) |
| **Krzych-Fałta 2018 [73]**, PL | Cross-sectional | 2006-2008 | General population | 18,617 | 6-7, 13-14, 20-44 | *Birth order*  Questionnaire | Questionnaire | **Criteria-based diagnosis of asthma (current)**  ↓ (2-3 vs 1)  NS (≥ 4 vs 1) |
| **Kutzora 2018 [76]**, DE | Cross-sectional | 2014-2015 | General population | 4,732 | 5.3 ± 0.5 | *Birth order*  Questionnaire | Questionnaire | **Clinician diagnosis of asthma (ever)**  ↑ (≥ 2 vs 1) |
| **Larsson 2008 [78]**, SE | Cohort | 2000-2005 | General population | 4,779 | 6-8 | *Sibship size*  Questionnaire | Questionnaire | **Clinician diagnosis of asthma (at age 3-8 years)**  NS (≥ 2 vs 1) |
| **Lee 2012 [81]**, KR | Cross-sectional | 2008 | General population | 1,749 | 9-12 | *Birth order*  Questionnaire | Questionnaire | **Clinician diagnosis of asthma (ever)**  ↓ (≥ 2 vs 1)  **Clinician diagnosis of asthma (ever) and wheezing in last year**  NS (≥ 2 vs 1) |
| **Lin 2019 [87]**, TW | Cohort | 2006-2015 | General population | 628,878 | ~6 | *Sibship size*  Register data | Medical records | **≥ 3 ambulatory visits or ≥ 1 hospital admission with clinician diagnosis of asthma and ≥ 1 ICS prescription (ever)**  ↓ (≥ 2 vs 1) |
| **McCandless 1970 [91]**, AU | Cross-sectional | 1965-1970 | Emotionally triggered asthma | 45 | 4-16 | *Birth order*  Questionnaire | Medical records | **Emotionally triggered asthma (current)**  ↑ (N/A) |
| **McKeever 2001 [92]**, GB | Cohort | N/A | General population | 29,238 | ≤ 11 | *Birth order*  Register data | Medical records | **Clinician diagnosis of asthma (ever)**  ↑ (≥ 2 vs 1)  **Clinician diagnosis of asthma (at age < 2 years)**  ↑ (≥ 2 vs 1)  **Clinician diagnosis of asthma (at age ≥ 2 years)**  NS (2 vs 1)  ↓ (≥ 3 vs 1) |
| **McLeod 2020 [93]**, CA | Cross-sectional | 2009-2017 | General population | ~2,263 | 3-11 | *Sibship size*  Questionnaire | Questionnaire | **Asthma (current)**  ↓ (≥ 2 vs 1) |
| **Midodzi 2007 [95]**, CA | Cohort | 1994-2003 | General population | 13,524 | ≤ 11 | *Birth order*  Questionnaire | Questionnaire | **2-year incidence of asthma in subjects aged ≤ 11 years**  NS (≥ 2 vs 1)  **Incidence of asthma during age 2-5 years**  NS (2 vs 1)  ↓ (≥ 3 vs 1) |
| **Morata-Alba 2019 [101]**, ES | Cohort | 2006-2016 | **Preterm group**  Born in week 32-35  **Full-term group**  Born in week ≥ 37 | 232 | 7-8 | *Birth order*  Questionnaire | Questionnaire | **Cough, wheezing, and/or dyspnea with good response to bronchodilators, and ≥ 1 episode of wheezing in last year (at end of follow-up)**  **Preterm group**  ↑ (≥ 2 vs 1)  **Full-term group**  NS (≥ 2 vs 1) |
| **Nafstad 2000 [102]**, NO | Cohort | 1992-1997 | General population | 2,531 | ~4 | *Birth order*  Questionnaire | Questionnaire | **Clinician diagnosis of asthma and asthma symptoms in last year (at end of follow-up)**  NS (≥ 2 vs 1)  **Clinician diagnosis of asthma and asthma symptoms in last year (at end of follow-up) and bronchial obstruction at age 0-2 years**  NS (≥ 2 vs 1)  **Clinician diagnosis of asthma and asthma symptoms in last year (at end of follow-up) and no bronchial obstruction at age 0-2 years**  ↓ (≥ 2 vs 1) |
| **Nafstad 2005 [103]**, NO | Cohort | 1992-2002 | General population | 2,540 | ~10 | *Birth order*  Questionnaire | Questionnaire | **Clinician diagnosis (ever)**  NS (≥ 2 vs 1)  **Clinician diagnosis of asthma (ever) and asthma symptoms in last year (at end of follow up)**  NS (≥ 2 vs 1) |
| **Nowak 1996 [106]**, DE | Cross-sectional | 1990-1992 | General population | 6,428 | 20-44 | *Sibship size*  Questionnaire | Medical examination | **Bronchial hyperresponsiveness (current)**  NS (≥ 2 vs 1) |
| **Ohfuji 2009 [107]**, JP | Cross-sectional | 2004-2005 | General population | 22,750 | 6-15 | *Birth order, sibship size*  Questionnaire | Questionnaire | **Asthma (ever) and wheezing in last year**  *Birth order*  NS (≥ 2 vs 1)  *Sibship size*  NS (2-3 vs 1)  ↓ (≥ 4 vs 1) |
| **Pekkanen 1999 [112]**, FI | Cross-sectional | N/A | General population | 8,387 | 13-14 | *Birth order, sibship size*  Questionnaire | Questionnaire | **Clinician diagnosis of asthma (ever)**  *Birth order*  NS (2 vs 1)  ↓ (≥ 3 vs 1)  *Sibship size*  NS (≥ 2 vs 1)  **Clinician diagnosis of asthma (ever) and ≥ 1 episode of wheezing in last year**  NS (≥ 2 vs 1) |
| **Pekkanen 2001 [113]**, FI | Cohort | 1966-1997 | General population | 5,192 | ~31 | *Birth order*  Questionnaire | Questionnaire | **Clinician diagnosis of asthma (ever)**  NS (≥ 2 vs 1) |
| **Ponsonby 1998 [116]**, AU | Cross-sectional | 1995 | General population | 6,158 | ~7 | *Birth order, sibship size*  Questionnaire | Questionnaire | **Asthma (ever)**  *Birth order*  ↓ (≥ 3 vs 2, for youngest child)  *Sibship size*  ↓ (2-5 vs 1)  NS (≥ 6 vs 1)  **Asthma (ever) and ≥ 1 episode of wheezing in last year**  *Sibship size*  ↓ (2-5 vs 1)  NS (≥ 6 vs 1) |
| **Ponsonby 1999 [117]**, AU | Cohort | 1988-1996 | Increased risk of SIDS, or part of multiple birth | 863 | ~7 | *Birth order, sibship size*  Questionnaire | Questionnaire | **Asthma (ever)**  *Birth order*  NS (≥ 2 vs 1)  *Sibship size*  ↓ (increase in sibship size during follow-up, per sibling)  **Asthma (ever) and ≥ 1 episode of wheezing in last year**  *Sibship size*  ↓ (≥ 2 vs 1) |
| **Räsänen 1997 [119]**, FI | Cohort | 1991-1993 | Twins | 2,680 | ~16 | *Birth order*  Questionnaire | Questionnaire | **Clinician diagnosis of asthma (ever)**  NS (≥ 2 vs 1) |
| **Räsänen 2000 [120]**, FI | Cohort | 1991-1995 | Twins | 4,578 | ~16 | *Birth order*  Questionnaire | Questionnaire | **Clinician diagnosis of asthma (ever)**  ↓ (≥ 3 vs 1-2) |
| **Rehalia 2020 [121]**, IN | Cross-sectional | N/A | Clinician diagnosis of asthma | 130 | 3-17 | *Birth order*  Interview | Clinician diagnosis | **Clinician diagnosis of asthma (current)**  Most common in second-born |
| **Rönmark 2009 [124]**, SE | Cohort | 1996, 2006 | General population | 3,848 | 7-8 | *Sibship size*  Questionnaire | Questionnaire | **Clinician diagnosis of asthma and positive SPT**  ↓ (≥ 2 vs 1)  **Clinician diagnosis and negative SPT**  NS (≥ 2 vs 1) |
| **Rönmark 2016 [125]**, SE | Cross-sectional | 2008 | General population | 18,087 | 16-75 | *Sibship size*  Questionnaire | Questionnaire | **Asthma (ever) and asthma medication use or recurrent wheezing or SOB in last year**  ↑ (≥ 2 vs 1) |
| **Salam 2004 [129]**, US | Nested case-control | N/A | Cases  Clinician diagnosis of asthma at age < 5 years  Controls  General population | 691 | 8-18 | *Sibship size*  Questionnaire | Questionnaire | **Clinician diagnosis of asthma at age < 5 years**  ↓ (≥ 4 vs 1)  **Clinician diagnosis of asthma at age < 5 years with early (< 3 years) transient wheezing**  NS (≥ 2 vs 1)  **Clinician diagnosis of asthma at age ≤ 3 years, with asthma symptoms or asthma medication use in last year**  NS (≥ 2 vs 1) |
| **Schmitz 2012 [132]**, DE | Cross-sectional | 2003-2006 | General population | 17,450 | ≤ 17 | *Birth order*  Questionnaire | Questionnaire | **Clinician diagnosis of asthma (ever)**  NS (≥ 2 vs 1) |
| **Sheldrake 1976a [134]**, GB | Cross-sectional | 1974 | General male population | ~4,740 | N/A (adults) | *Birth order*  Questionnaire | Questionnaire | **Asthma (in last 5 years)**  NS (≥ 2 vs 1) |
| **Sheldrake 1976b [135]**, GB | Cross-sectional | 1974 | General female population | ~3,282 | N/A (adults) | *Birth order*  Questionnaire | Questionnaire | **Asthma (in last 5 years)**  NS (≥ 2 vs 1) |
| **Shohat 2002 [137]**, IL | Cross-sectional | 1997 | General population | 10,057 | 13-14 | *Birth order*  Questionnaire | Questionnaire | **Asthma (current)**  ↓ (≥ 4 vs < 4) |
| **Slob 2020 [139]**, NL, SE | Cohort | 1989-2016, 2005-2019 | Twins | 43,281 | 3-10 | *Birth order*  Questionnaire | Questionnaire | **Asthma (ever)**  NS (≥ 2 vs 1) |
| **Smith 1960 [140]**, GB | Cross-sectional | 1953-1958 | Clinician diagnosis of asthma | 518 | N/A | *Sibship size*  N/A | Clinician diagnosis | **Clinician diagnosis of asthma (current)**  NS (N/A) |
| **Strachan 2015 [141]**, AR, BB, BE, BO, BR, CA, CI, CL, CM, CN, CO, EC, EE, ES, ET, FI, FJ, GA, GB, HK, HU, ID, IM, IN, IR, JP, KG, KR, KW, LT, MA, MK, MT, MX, MY, NG, NZ, OM, PA, PE, PH, PL, PT, SD, SG, SY, TH, TK, TW, US, UY, VE, WS, ZA | Cross-sectional | 2000-2003 | General population | 547,426 | 6-7, 13-14 | *Birth order, sibship size*  Questionnaire | Questionnaire | **Asthma (ever)**  **6-7-year-olds**  *Birth order*  NS (per older sibling)  *Sibship size*  NS (≥ 2 vs 1)  **13-14-year-olds**  *Birth order*  ↓ (per older sibling)  *Sibship size*  ↓ (≥ 4 vs 1) |
| **Stråvik 2020 [142]**, SE | Cohort | 2015-2019 | General population | 508 | ~1 | *Birth order*  Questionnaire, medical records | Clinician diagnosis | **Clinician diagnosis of asthma (current)**  NS (≥ 2 vs 1) |
| **Sunyer 1997 [144]**, ES | Cross-sectional | 1992-1993 | General population | 2,646 | 20-44 | *Birth order*  Questionnaire | Questionnaire and medical examination | **Asthma (ever) and asthma attack in last year**  ↓ (≥ 4 vs 1)  **Asthma (ever) and bronchial hyperresponsiveness (current)**  NS (≥ 2 vs 1)  **Asthma (ever; onset-age ≤ 15 years) and asthma attack in last year**  NS (≥ 2 vs 1)  **Asthma (ever; onset-age > 15 years) and asthma attack in last year**  NS (≥ 2 vs 1) |
| **Toppila-Salmi 2019 [147]**, FI | Case-control | N/A | **Cases**  Clinician diagnosis of asthma at age > 30 years  **Controls**  General population | 2,890 | 31-71 | *Birth order, sibship size*  Questionnaire | Register data and questionnaire | **Recent (within 1 year) clinician diagnosis of asthma at age > 30 years and current asthma medication**  NS (≥ 2 vs 1) |
| **Toppila-Salmi 2021 [148]**, FI | Case-control | 2020 | **Cases**  Clinician diagnosis of asthma at age ≥ 16 years with regular and severe impairment and ≥ 1 OCS course per year or regular OCS use or nocturnal symptoms/wheezing ≥ a few times per month  **Controls**  Clinician diagnosis of asthma at age ≥ 16 years | 1,350 | 31-93 | *Birth order, sibship size*  Questionnaire | Register data and questionnaire | **Recent (within 2 years) clinician diagnosis of asthma with onset-age ≥ 16 years and regular and severe impairment, and ≥ 1 OCS course per year or regular OCS use or nocturnal symptoms/wheezing ≥ a few times per month (current)**  *Birth order*  NS (≥ 2 vs 1)  *Sibship size*  ↑ (≥ 3 vs < 3)  **Recent (within 2 years) clinician diagnosis of asthma with onset-age ≥ 16 years and ≥ 2 OCS courses per year or regular OCS use (current)**  *Birth order*  NS (≥ 2 vs 1)  *Sibship size*  NS (≥ 3 vs < 3) |
| **Van Beijsterveldt 2008 [149]**, NL | Cohort | 1986-2006 | Twins | 12,009 | ~5 | *Birth order*  Questionnaire | Questionnaire | **Clinician diagnosis of asthma (ever)**  NS (≥ 2 vs 1) |
| **Venter 2021 [151]**, US | Cohort | 2009-2020 | General population | 1,261 | 4-8 | *Birth order*  Questionnaire | Medical records | **Clinician diagnosis of asthma (ever)**  ↓ (≥ 2 vs 1) |
| **Victorino 2009 [152]**, US | Cross-sectional | 2003-2004 | General population | 102,353 | ≤ 17 | *Sibship size*  Questionnaire | Questionnaire | **Asthma (current)**  ↓ (no. of siblings) |
| **Westergaard 2005 [153]**, DK | Cross-sectional | 1997-2000 | Pregnant women | 31,145 | 15-43 | *Birth order, sibship size*  Register data | Questionnaire | **Clinician diagnosis of asthma (ever), with allergic rhinitis** *Birth order*  ↓ (≥ 2 vs 1)  *Sibship size*  ↓ (≥ 5 vs 1)  **Clinician diagnosis of asthma (ever), no allergic rhinitis**  *Birth order*  NS (≥ 2 vs 1)  *Sibship size*  NS (≥ 2 vs 1) |
| **Wickens 1999 [154]**, NZ | Case-control | 1994-1995 | **Cases**  Self-reported asthma symptoms and medication use in last year  **Controls**  General population, with no history of wheezing | 474 | 7-9 | *Birth order, sibship size*  Questionnaire | Questionnaire | **Clinician diagnosis of asthma (ever) and asthma medication use in last year**  *Birth order*  NS (≥ 3 vs 1)  ↓ (≥ 3 vs 2)  *Sibship size*  ↓ (≥ 2 vs 1) |
| **Zadnick 2010 [157]**, US | Case-control | 1998-2001 | Twins | 5,992 | 21-47 | *Birth order, sibship size*  Questionnaire | Questionnaire | **Asthma (any onset-age)**  *Birth order*  ↓ (≥ 3 vs 1-2)  *Sibship size*  NS (≥ 4 vs 2-3)  **Asthma (onset-age < 8 years)**  **All**  *Sibship size*  NS (≥ 4 vs 2-3)  **Discordant twins**  *Birth order*  ↓ (≥ 3 vs 1-2)  **Concordant twins**  *Birth order*  NS (≥ 3 vs 1-2)  **Asthma (onset-age ≥ 8 years)**  *Birth order*  NS (≥ 3 vs 1-2)  *Sibship size*  NS (≥ 4 vs 2-3) |

**Column explanations. ^a^** The highest number of subjects in relevant analyses, or – when such numbers were not defined – the number of subjects included in the study. ^b^ Reported in years as either: (a) average ± standard deviation; (b) minimum age-maximum age; or (c) ~approximate age. ^c^ Black bold titles indicate specific outcomes, blue bold titles indicate specific subgroups of subjects, and italic titles indicate analyses of specific exposure. For each analysis, the results are described as (a) NS (non-significant 95% confidence interval); (b) ↑ (significantly increased risk of outcome); or (c) ↓ (significantly decreased risk of outcome). In parenthesis, the sibship size or birth order from which the results are significant is indicated. **Country codes.** AR: Argentina. AT: Austria. AU: Australia. BB: Barbados. BD: Bangladesh. BE: Belgium. BO: Bolivia (Plurinational State of). BR: Brazil. CA: Canada. CI: Côte d'Ivoire. CL: Chile. CM: Cameroon. CN: China. CO: Colombia. DE: Germany. DK: Denmark. EC: Ecuador. EE: Estonia. ES: Spain. ET: Ethiopia. FI: Finland. FJ: Fiji. GA: Gabon. GB: United Kingdom of Great Britain and Northern Ireland. GH: Ghana. HK: Hong Kong. HU: Hungary. ID: Indonesia. IL: Israel. IM: Isle of Man. IN: India. IR: Iran (Islamic Republic of). IT: Italy. JP: Japan. KG: Kyrgyzstan. KR: Korea, Republic of. KW: Kuwait. LT: Lithuania. MA: Morocco. MK: North Macedonia. MT: Malta. MX: Mexico. MY: Malaysia. NC: New Caledonia. NG: Nigeria. NL: Netherlands. NO: Norway. NZ: New Zealand. OM: Oman. PA: Panama. PE: Peru. PF: French Polynesia. PH: Philippines. PL: Poland. PT: Portugal. SD: Sudan. SE: Sweden. SG: Singapore. SY: Syrian Arab Republic. TH: Thailand. TK: Tokelau. TR: Turkey. TW: Taiwan, Province of China. US: United States of America. UY: Uruguay. VE: Venezuela (Bolivarian Republic of). WS: Samoa. ZA: South Africa. **Other abbreviations.** ICS: inhaled corticosteroids. N/A: not available. NS: non-significant. OCS: oral corticosteroids. SIDS: sudden infant death syndrome. SOB: (attack of) shortness of breath. SPT: skin prick test.

### Table S4B. Table of characteristics for wheezing

| **Study, country** | **Study design** | **Years of data collection** | **Source of study population** | **Subjects analyzed^a^** | **Age (in years)^b^** | **Exposure and assessment method** | **Outcome assessment method** | **Main findings^c^** |
| --- | --- | --- | --- | --- | --- | --- | --- | --- |
| **Adler 2005 [3]**, US | Cross-sectional | 2003 | Rural population | 4,152 | 10.1 ± 3.2 | *Sibship size*  Questionnaire | Questionnaire | **≥ 1 wheezing episode (unspecified time frame)**  NS (no. of siblings) |
| **Ayuk 2018 [8]**, CM, ET, GA, MA, NG, SD, ZA | Cross-sectional | 2001-2002 | General population | 28,391 | 13-14 | *Birth order*  Questionnaire | Questionnaire | **≥ 1 wheezing episode (in last year)**  ↓ (≥ 3 vs < 3) |
| **Baker 1998 [10]**, GB | Cohort | 1991-1993 | General population | 8,501 | ~0.67 | *Birth order*  Questionnaire | Questionnaire | **≥ 1 wheezing episode (in first 6 months of life)**  ↑ (≥ 2 vs 1)  **≥ 3 wheezing episodes (in first 6 months of life)**  NS (≥ 2 vs 1) |
| **Ball 2000 [11]**, US | Cohort | 1980-1997 | General population | 1,035 | ~13 | *Birth order*  Questionnaire | Questionnaire | **≥ 4 wheezing episodes (during age 1-2 years)**  ↑ (≥ 3 vs < 3, or attending day care)  **≥ 4 wheezing episodes (during age 5-6 years)**  NS (≥ 3 vs < 3, or attending day care)  **≥ 4 wheezing episodes (during age 12-13 years)**  ↓ (≥ 3 vs < 3, or attending day care) |
| **Bercedo-Sanz 2015 [14]**, ES | Cross-sectional | 2008-2011 | General population | 958 | 1-1.5 | *Sibship size*  Questionnaire | Questionnaire | **≥ 1 wheezing episode (in last year)**  ↑ (≥ 2 vs 1) |
| **Bodner 2000 [17]**, GB | Nested case-control | 1995 | **Cases**  Self-reported adult-onset wheezing  **Controls**  General population | 319 | 39-45 | *Birth order,*  *Sibship size*  Questionnaire | Questionnaire | **≥ 1 wheezing episode (onset-age ≥ 15 years)**  NS (≥ 2 vs 1)  **≥ 1 wheezing episode and cough or phlegm ≥ 3 months per year (onset-age ≥ 15 years)**  NS (≥ 2 vs 1) |
| **Burr 1997 [21]**, GB | Cohort | N/A | Children with high risk of allergy | 440 | ~7 | *Birth order*  Questionnaire | Questionnaire | **≥ 1 wheezing episode (in last year)**  **Non-atopics**  Significant association in unspecified direction (no. of older siblings)  **Atopics**  NS (no. of older siblings) |
| **Campo 2006 [22]**, US | Cohort | 2003-2004 | General population | 532 | ~1 | *Sibship size*  Questionnaire | Questionnaire | **≥ 1 wheezing episode (in first year of life)**  ↑ (≥ 3 vs 1)  **≥ 2 wheezing episodes (in first year of life)**  ↑ (≥ 3 vs 1)  **≥ 1 wheezing episode and atopy (positive SPT) vs 0-1 episodes and no atopy (in first year of life)**  NS (≥ 3 vs 1)  **≥ 2 wheezing episodes requiring medical intervention/clinician visit or resulting in ≥ 1 sleep disturbance vs 0-1 episodes (in first year of life)**  ↑ (≥ 3 vs 1) |
| **Cifuentes 2003 [24]**, CL | Cohort | 1999-2000 | Inpatient population admitted for bronchiolitis | 77 | 1-3 | *Sibship size*  Interview | Clinician diagnosis | **Wheezing episodes treated by clinician (in last year)**  **All**  NS (no. of siblings)  **RSV-negative**  ↑ (no. of siblings) |
| **Cooper 2014 [26]**, EC | Cross-sectional | 2005-2010 | General population | 6,821 | 5-16 | *Birth order*  Questionnaire | Questionnaire | **≥ 1 wheezing episode (in last year)**  ↓ (≥ 5 vs < 5) |
| **Davis 1981 [28]**, GB | Case-control | N/A | **Cases**  Wheezing in last 10 years  **Controls**  General population | ~776 | ≤ 15 | *Sibship size*  Interview/questionnaire | Medical records | **≥ 2 wheezing episodes in ≥ 2 years (in last 10 years)**  NS (≥ 3 vs 2) |
| **De Meer 2005 [29]**, NL | Cross-sectional | 1997-1998* | General population | 1,555 | 8-13 | *Birth order*  Questionnaire | Questionnaire | **≥ 1 wheezing episode (in last year)**  NS (≥ 2 vs 1) |
| **Elder 1996 [31]**, AU | Cohort | 1990-1992 | Preterm infants | 525 | ~1 | *Sibship size*  Interview | Questionnaire | **≥ 2 wheezing episodes treated with bronchodilator (in first year of life)**  ↑ (≥ 2 vs 1) |
| **Farooqi 1998 [32]**, GB | Cohort | 1975-1996 | General population | 1,934 | 12-21 | *Birth order*  Medical records | Medical records | **Recurrent wheezing (at age ≥ 3 years)**  NS (≥ 2 vs 1) |
| **Foliaki 2008 [33]**, FJ, NC, PF, TK, WS | Cross-sectional | 2002-2003 | General population | 17,683 | 6-14 | *Birth order*  Questionnaire | Questionnaire | **≥ 1 wheezing episode (in last year)**  ↓ (≥ 3 vs 1) |
| **Freitas 2012 [34]**, BR | Cross-sectional | 2007-2009 | General population | 400 | 5-8 | *Birth order*  Questionnaire | Questionnaire | **≥ 1 wheezing episode (in last year)**  ↑ (≥ 3 vs < 3) |
| **Gao 2005 [36]**, CA | Cohort | 1994-1999 | General population | 1,223 | ≤ 11 | *Birth order, sibship size*  Questionnaire | Questionnaire | **Prevalence of wheezing (unspecified time frame)**  ↓ (2 vs 1)  NS (≥ 3 vs 1)  **Incidence of wheezing (unspecified time frame)**  ↑ (3 vs 1-2) |
| **Gao 2021 [37]**, CN | Cohort | 2012-2019 | General population | 21,716 | ≤ 18 | *Birth order*  Medical records | Medical records | **≥ 1 wheezing episode or related symptoms/lung auscultation findings, e.g., coughing, sputum (current)**  ↑ (≥ 2 vs 1) |
| **Goldstein 2005 [39]**, US | Cohort | N/A | Low-income population | ~253 | ~3 | *Birth order*  Questionnaire | Questionnaire | **≥ 1 wheezing episode (in last 3 months at age 1 year)**  NS (≥ 2 vs 1)  **≥ 1 wheezing episode (in last 3 months at age 2 years)**  NS (≥ 2 vs 1)  **≥ 1 wheezing episode (in last 3 months at age 3 years)**  NS (≥ 2 vs 1) |
| **Gray 2019 [41]**, AU | Cohort | 2010-2014 | General population | 840 | ~1 | *Birth order*  Birth records | Questionnaire | **≥ 1 wheezing episode (in first year of life)**  NS (≥ 2 vs 1)  **≥ 2 wheezing episodes (in first year of life)**  NS (≥ 2 vs 1)  **≥ 2 wheezing episodes with SOB (in first year of life)**  NS (≥ 2 vs 1)  **≥ 1 wheezing episode and positive SPT (in first year of life)**  NS (≥ 2 vs 1) |
| **Greenough 2005 [42]**, AU, GB, IE, SG | Cohort | 1998-2003 | Preterm infants | 431 | ~1 | *Birth order*  Questionnaire | Questionnaire | **≥ 1 wheezing episode (in first year of life)**  ↑ (≥ 2 vs 1) |
| **Gustafsson 1996 [44]**, SE | Cohort | 1991-1993 | General population | 638 | ~1.5 | *Sibship size*  Questionnaire | Questionnaire | **≥ 1 wheezing episode (in first 18 months of life)**  ↑ (≥ 2 vs 1) |
| **Hallit 2018 [46]**, FR | Cohort | 2011-2012 | General population | 14,059 | ~1 | *Sibship size*  Questionnaire | Questionnaire | **Wheezing at 2 months but not 12 months vs never wheezing**  ↑ (2-3 vs 1)  NS (≥ 4 vs 1)  **Wheezing at 2 months and 12 months vs at 2 months but not 12 months**  ↑ (2 vs 1)  NS (≥ 3 vs 1)  **Wheezing at both 2 months and 12 months vs never wheezing**  ↑ (≥ 2 vs 1) |
| **Hedman 2015 [47]**, SE | Cohort | 1996-2007 | General population | 2,754 | ~19 | *Sibship size*  Questionnaire | Questionnaire | **≥ 1 wheezing episode (in last year)**  **All**  ↓ (no. of siblings) **Atopic (positive SPT)**  NS (no. of siblings)  **Non-atopic (negative SPT)**  ↓ (no. of siblings) |
| **Herr 2012 [49]**, FR | Cohort | 2003-2008 | General population | 1,879 | ~1.5 | *Sibship size*  Questionnaire | Questionnaire | **≥ 1 wheezing episode (in first 18 months of life)**  ↑ (≥ 2 vs 1)  **≥ 1 instance of hospital-based care (due to bronchiolitis, wheezy bronchitis, or asthma crisis) or wheezing requiring ICS treatment (in first 18 months of life)**  ↑ (≥ 2 vs 1) |
| **Higgins 2021 [51]**, GB | Cohort | 2010-2018 | General population | 412 | ~1 | *Birth order*  Questionnaire | Questionnaire | **≥ 1 wheezing episode (in first year of life)**  ↑ (2 vs 1)  NS (3 vs 1)  **≥ 1 wheezing episode associated with infection (in first year of life)**  ↑ (2 vs 1)  NS (3 vs 1)  **≥ 1 wheezing episode not associated with infection (in first year of life)**  NS (2 vs 1)  ↑ (3 vs 1) |
| **Hijazi 2000 [52]**, SA | Case-control | 1998 | **Cases**  Self-reported asthma  **Controls**  General population | 316 | ~12 | *Sibship size*  Questionnaire | Questionnaire | **≥ 1 wheezing episode (in last year)**  NS (N/A) |
| **Hosoki 2009 [53]**, JP | Cohort | 2006-2008 | Inpatients, admitted for RSV infection | 58 | N/A (preschool children) | *Sibship size*  Questionnaire | Questionnaire | **≥ 1 wheezing episode (during follow-up [1-2.5 years])**  NS (≥ 2 vs 1) |
| **Jarvis 1997 [58]**, GB | Cross-sectional | 1990-1991 | General population | 1,159 | 20-44 | *Birth order, sibship size*  Questionnaire | Questionnaire | **≥ 1 wheezing episode with SOB (in last year)**  *Birth order*  NS (≥ 2 vs 1)  *Sibship size*  NS (≥ 2 vs 1)  ↓ (per sibling)  **≥ 1 wheezing episode not associated with infection (in last year)**  *Birth order*  NS (≥ 2 vs 1)  *Sibship size*  NS (≥ 2 vs 1)  ↓ (per sibling) |
| **Jedrychowski 2009 [59]**, PL | Cohort | 2001-2006 | General population | 468 | ~2 | *Birth order*  Questionnaire | Questionnaire | **≥ 1 wheezing episode (in first year of life)**  NS (≥ 2 vs 1)  **≥ 1 wheezing episode (at age 13-24 months)**  ↑ (≥ 2 vs 1)  **Wheezing with onset-age ≤ 12 months and persisting in second year of life**  ↑ (≥ 2 vs 1) |
| **Kearney 1998 [65]**, IE | Cross-sectional | 1997 | Travelling and settled families, respectively | 173 | 6-12 | *Sibship size*  Questionnaire | Questionnaire | **≥ 1 wheezing episode (in last year)**  NS (N/A) |
| **Kramer 2009 [72]**, BY | Cohort | 1996-2005 | General population | 13,889 | ~6.5 | *Birth order*  Questionnaire | Questionnaire | **≥ 1 wheezing episode (in last year)**  NS (≥ 2 vs 1) |
| **Kusunoki 2012 [75]**, JP | Cross-sectional | 2006 | General population | 11,454 | 7-15 | *Birth order*  Questionnaire | Questionnaire | **≥ 1 wheezing episode (in first year of life)**  ↑ (2 vs 1)  NS (≥ 3 vs 1)  **≥ 2 wheezing episodes with SOB (in last 2 years)**  NS (≥ 2 vs 1) |
| **Kutzora 2018 [76]**, DE | Cross-sectional | 2014-2015 | General population | 4,732 | 5.3 ± 0.5 | *Birth order*  Questionnaire | Questionnaire | **≥ 1 wheezing episode associated with infection (ever)**  NS (≥ 2 vs 1)  **Wheezing with symptoms between distinct wheezing episodes or not associated with infection (ever)**  ↑ (≥ 2 vs 1)  **≥ 4 wheezing episodes (in last year)**  NS (≥ 2 vs 1) |
| **Larenas-Linnemann 2020 [77]**, MX | Cross-sectional | 2009-2011 | General population | 703 | ~1 | *Sibship size*  Questionnaire | Questionnaire | **≥ 1 wheezing episode and atopy (allergic skin disease or atopic first-degree relative; in first year of life)**  NS (≥ 2 vs 1)  **≥ 1 wheezing episode with no atopy (in first year of life)**  ↓ (≥ 2 vs 1) |
| **Latzin 2007 [79]**, CH | Cohort | 1999-2006 | General population | 195 | ~1 | *Birth order*  Questionnaire | Questionnaire | **Weeks with wheezing (in first year of life)**  NS (2 vs 1)  ↑ (≥ 3 vs 1) |
| **Lee 2004 [80]**, HK | Cross-sectional | 1994-1995, 2000-2001 | General population | 4,448 | 6-7 | *Sibship size*  Questionnaire | Questionnaire | **Woken by wheeze at night (in last year)**  NS (N/A) |
| **Lee 2012 [81]**, KR | Cross-sectional | 2008 | General population | 1,749 | 9-12 | *Birth order*  Questionnaire | Questionnaire | **≥ 1 wheezing episode (ever)**  NS (≥ 2 vs 1) |
| **Lemanske 2005 [82]**, US | Cohort | 1998-2003 | Children with high risk of allergic respiratory disease | 275 | ~3 | *Birth order*  N/A | Questionnaire | **≥ 1 wheezing episode (in last year)**  ↑ (≥ 2 vs 1) |
| **Lewis 1995 [83]**, GB | Cohort | 1970-1986 | General population | 15,712 | ~16 | *Birth order*  Questionnaire | Questionnaire | **≥ 1 wheezing episode (ever)**  **5-year-olds**  NS (≥ 2 vs 1)  **≥ 1 wheezing episode (in last year and at age ≤ 5 years)**  **16-year-olds**  NS (≥ 2 vs 1) |
| **Lewis 1998 [85]**, GB | Cohort | 1970-1986 | General population | 17,427 | ~16 | *Birth order*  Questionnaire | Questionnaire | **≥ 1 wheezing episode (ever)**  NS (≥ 2 vs 1)  **≥ 1 wheezing episode (in last year)**  NS (≥ 2 vs 1) |
| **Lima 2010 [86]**, BR | Cross-sectional | 2006 | General population | 1,013 | ~1 | *Sibship size*  Questionnaire | Questionnaire | **≥ 1 wheezing episode (in first year of life)**  ↑ (≥ 2 vs 1) |
| **Linneberg 2006 [88]**, DK | Cohort | 2000-2004 | General population | 34,793 | ~1.5 | *Birth order*  Interview | Interview | **≥ 1 wheezing episode (in first 18 months of life)**  ↑ (≥ 2 vs 1) |
| **Loss 2016 [89]**, AT, CH, DE, FI, FR | Cohort | 2002-2011 | Rural population | 983 | ~6 | *Birth order*  Questionnaire | Questionnaire | **≥ 1 wheezing episode (in first year of life)**  ↑ (≥ 2 vs 1) |
| **Martins 2015 [90]**, PT | Cohort | 2011-2013 | Inpatients, admitted for bronchiolitis | 79 | 1-1.5 | *Sibship size*  Interview | Interview and medical records | **≥ 4 wheezing episodes (in last year)**  NS (N/A) |
| **Mejias 2018 [94]**, DO | Cross-sectional | 2017 | General population | 600 | 3-11 | *Birth order*  Questionnaire | Questionnaire | **≥ 1 wheezing episode (in last year)**  ↓ (2 vs 1)  NS (≥ 3 vs 1) |
| **Midodzi 2007 [95]**, CA | Cohort | 1994-2003 | General population | 13,524 | ≤ 11 | *Birth order*  Questionnaire | Questionnaire | **≥ 1 wheezing episode (at age 2-5 years)**  NS (2 vs 1)  ↓ (≥ 3 vs 1) |
| **Miyake 2004 [96]**, JP | Cross-sectional | 2001 | General population | 5,539 | 12-15 | *Birth order*  Questionnaire | Interview and medical records | **≥ 1 wheezing episode (in last year)**  NS (≥ 2 vs 1) |
| **Miyake 2011 [97]**, JP | Cross-sectional | 2007-2008 | Pregnant women | 1,745 | N/A (adults) | *Birth order, sibship size*  Questionnaire | Questionnaire | **≥ 1 wheezing episode (in last year)**  NS (≥ 2 vs 1) |
| **Moncayo 2010 [99]**, EC | Cross-sectional | 2005-2007 | General population | 3,858 | 6-16 | *Birth order*  Questionnaire | Questionnaire | **≥ 1 wheezing episode (in last year)**  ↓ (≥ 4 vs 1-3)  **≥ 1 wheezing episode and positive SPT (in last year)**  NS (≥ 4 vs 1-3)  **≥ 1 wheezing episode and negative SPT (in last year)**  ↓ (≥ 4 vs 1-3) |
| **Moraes 2013 [100]**, BR | Cross-sectional | 2005-2007 | General population | 294 | ~1 | *Sibship size*  Questionnaire | Questionnaire | **≥ 1 wheezing episode (in first year of life)**  ↑ (≥ 2 vs 1) |
| **Nicolai 2017 [104]**, IT | Cohort | 2009-2017 | **Exposed**  Inpatients, admitted for virus-induced ARTI  **Unexposed**  Outpatients with no history of disease, at pediatric clinic | 374 | 3-6 | *Sibship size*  Questionnaire | Questionnaire | **≥ 2 pediatrician-diagnosed wheezing episodes per year (in last 3 years)**  NS (N/A) |
| **Nicolaou 2008 [105]**, GB | Cohort | 1995-2003 | General population | 922 | ~5 | *Birth order*  Questionnaire | Questionnaire | **≥ 1 wheezing episode (in last year)**  NS (≥ 2 vs 1) |
| **Ohfuji 2009 [107]**, JP | Cross-sectional | 2004-2005 | General population | 22,750 | 6-15 | *Birth order, sibship size*  Questionnaire | Questionnaire | **≥ 1 wheezing episode (in last year)**  NS (≥ 2 vs 1) |
| **Oliveira-Santos 2015 [108]**, BR | Cross-sectional | 2011-2012 | General population | 3,009 | 13-14 | *Birth order*  Questionnaire | Questionnaire | **≥ 1 wheezing episode (in last year)**  ↓ (≥ 2 vs 1) |
| **Oluwole 2013 [109]**, NG | Cross-sectional | N/A | General population | 1,736 | 13-14 | *Birth order, sibship size*  Questionnaire | Questionnaire | **≥ 1 wheezing episode (in last year)**  *Birth order*  ↑ (≥ 2 vs 1)  *Sibship size*  NS (2-5 vs 1)  ↑ (≥ 6 vs 1) |
| **Özmert 2009 [110]**, TR | Cross-sectional | 2004 | General population | 109 | 2-4 | *Birth order*  Questionnaire | Questionnaire | **≥ 1 wheezing episode (ever)**  NS (≥ 2 vs 1) |
| **Parthasarathi 2021 [111]**, IN | Cross-sectional | 2018 | General population | 7,804 | 9.6 ± 2.6 | *Sibship size*  Questionnaire | Questionnaire | **≥ 1 wheezing episode (ever)**  ↑ (≥ 2 vs 1) |
| **Pérez Tarazona 2010 [114]**, ES | Cohort | 2007-2009 | General population | 620 | ~0.5 | *Birth order*  Questionnaire | Medical records | **≥ 1 wheezing episode treated at primary care or hospital (in first 6 months of life)**  ↑ (≥ 2 vs 1) |
| **Perzanowski 2008 [115]**, US | Cross-sectional | N/A | Low-income population with high risk of asthma | 1,005 | ~4 | *Birth order*  Questionnaire | Questionnaire | **≥ 1 wheezing episode (in last year)**  ↑ (≥ 2 vs 1)  **≥ 4 wheezing episodes (in last year)**  ↑ (≥ 2 vs 1) |
| **Ponsonby 2003 [118]**, AU | Cohort | 1988-1989, 1997 | General population | 498 | ~8 | *Sibship size*  Questionnaire | Questionnaire | **≥ 1 wheezing episode (in last year)**  NS (per sibling) |
| **Rusconi 1999 [126]**, IT | Cross-sectional | 1994-1995 | General population | 16,333 | 6-7 | *Sibship size*  Questionnaire | Questionnaire | **≥ 1 wheezing episode during age 0-2 years, no wheezing in last year**  ↑ (≥ 2 vs 1)  **≥ 1 wheezing episode during age 0-2 years and in last year**  NS (≥ 2 vs 1)  **No wheezing during age 0-2 years, ≥ 1 episode in last year**  ↓ (≥ 2 vs 1) |
| **Rusconi 2005 [127]**, IT | Cross-sectional | 2002 | General population | 20,016 | 6-7 | *Sibship size*  Questionnaire | Questionnaire | **≥ 1 wheezing episode during age 0-2 years, no wheezing in last year**  ↑ (≥ 2 vs 1)  **≥ 1 wheezing episode during age 0-2 years and in last year**  NS (≥ 2 vs 1)  **No wheezing during age 0-2 years, ≥ 1 episode in last year**  ↓ (≥ 2 vs 1) |
| **Rutter 2020 [128]**, AR, BB, BE, BO, BR, CA, CI, CL, CN, CO, EC, EE, ES, GA, GB, HK, HU, ID, IM, IN, IR, KG, LT, MA, MK, MX, MY, NG, NZ, OM, PA, PE, PH, PL, PT, SD, SY, TH, TW, UY, VE, ZA | Cross-sectional | 2000-2003 | General population | 341,299 | 6-7, 13-14 | *Sibship size*  Questionnaire | Questionnaire | **≥ 1 wheezing episode (in last year)**  NS (≥ 3 vs 1-2) |
| **Saya 2012 [130]**, IN | Cross-sectional | 2012 | Rural population | 275 | 12-15 | *Birth order*  Questionnaire | Questionnaire | **≥ 1 wheezing episode (in last year)**  NS (≥ 2 vs 1) |
| **Saya 2014 [131]**, IN | Cross-sectional | 2012 | Urban population | 263 | 12-16 | *Birth order*  Questionnaire | Questionnaire | **≥ 1 wheezing episode (in last year)**  NS (≥ 2 vs 1) |
| **Sears 1996 [133]**, NZ | Cohort | 1972-1990 | General population | 1,037 | ~18 | *Birth order*  Questionnaire | Questionnaire | **Recurrent wheezing (in last 2-3 years)**  NS (≥ 2 vs 1) |
| **Sherriff 2001 [136]**, GB | Cohort | 1991-1996 | General population | 8,594 | ~3.5 | *Birth order*  N/A | Questionnaire | **≥ 1 wheezing episode at age 0-6 months**  ↑ (≥ 2 vs 1)  **≥ 1 wheezing episode at age 0-6 months and at 30-42 months**  NS (2 vs 1)  ↑ (≥ 3 vs 1)  **No wheezing at age 0-6 months, ≥ 1 wheezing episode at age 30-42 months**  NS (≥ 2 vs 1) |
| **Shohat 2002 [137]**, IL | Cross-sectional | 1997 | General population | 10,057 | 13-14 | *Birth order*  Questionnaire | Questionnaire | **≥ 1 wheezing episode (in last year)**  NS (≥ 4 vs < 4) |
| **Simões 2019 [138]**, BR | Cross-sectional | 2014-2015 | Prematurely born individuals | 445 | 1.5-4.5 | *Sibship size*  Questionnaire | Questionnaire | **≥ 3 wheezing crisis episodes (in last year or first year of life)**  NS (2 vs 1)  ↑ (≥ 3 vs 1) |
| **Strachan 2015 [141]**, AR, BB, BE, BO, BR, CA, CI, CL, CM, CN, CO, EC, EE, ES, ET, FI, FJ, GA, GB, HK, HU, ID, IM, IN, IR, JP, KG, KR, KW, LT, MA, MK, MT, MX, MY, NG, NZ, OM, PA, PE, PH, PL, PT, SD, SG, SY, TH, TK, TW, US, UY, VE, WS, ZA | Cross-sectional | 2000-2003 | General population | 547,426 | 6-7, 13-14 | *Birth order, sibship size*  Questionnaire | Questionnaire | **≥ 1 wheezing episode (ever)**  **6-7-year-olds**  *Birth order*  ↓ (per older sibling)  *Sibship size*  ↓ (≥ 3 vs 1)  **13-14-year-olds**  *Birth order*  ↓ (per older sibling)  *Sibship size*  ↓ (≥ 2 vs 1)  **≥ 1 wheezing episode (in last year)**  **6-7-year-olds**  NS (≥ 2 vs 1)  **13-14-year-olds**  NS (≥ 2 vs 1) |
| **Sugiura 2021 [143]**, JP | Cross-sectional | 2016-2018 | General population | 19,104 | ~0.3 | *Sibship size*  Questionnaire | Questionnaire | **≥ 1 wheezing episode (in first 4 months of life)**  ↑ (≥ 2 vs 1)  **≥ 1 wheezing episode with hospital/clinic visit (in first 4 months of life)**  ↑ (≥ 2 vs 1)  **≥ 1 wheezing episode with hospitalization (in first 4 months of life)**  ↑ (≥ 2 vs 1) |
| **Svanes 2002 [145]**, AU, BE, CH, DE, EE, ES, FR, GB, IE, IS, IT, NL, NO, NZ, SE, US | Cross-sectional | 1990-1995 | General population | 18,530 | 20-44 | *Sibship size*  Questionnaire | Questionnaire | **≥ 1 wheezing episode (in last year)**  NS (≥ 2 vs 1)  **≥ 1 wheezing episode with SOB (in last year)**  NS (≥ 2 vs 1)  **≥ 1 wheezing episode not associated with infection (in last year)**  NS (≥ 2 vs 1) |
| **Teijeiro 2017 [146]**, AR | Cross-sectional | 2013-2014 | General population | 1,031 | 1-1.5 | *Sibship size*  Questionnaire | Questionnaire | **3-5 wheezing episodes vs 1-2 wheezing episode (in last year)**  ↑ (≥ 2 vs 1) |
| **Venero-Fernández 2013 [150]**, CU | Cross-sectional | 2010-2011 | General population | 1,956 | ~1 | *Birth order, sibship size*  Questionnaire | Questionnaire | **≥ 1 wheezing episode (in last year)**  *Birth order*  NS (≥ 2 vs 1)  *Sibship size*  ↑ (≥ 2 vs 1) |
| **Wolff 2012 [155]**, MG | Cross-sectional | 2009 | General population | 1,236 | 7-14 | *Birth order, sibship size*  Questionnaire | Questionnaire | **≥ 1 wheezing episode (ever)**  *Birth order*  ↓ (no. of older siblings)  *Sibship size*  NS (no. of siblings) |
| **Venter 2021 [151]**, US | Cohort | 2009-2020 | General population | 1,261 | 4-8 | *Birth order*  Questionnaire | Medical records | **≥ 1 wheezing episode (ever)**  NS (≥ 2 vs 1) |
| **Zekveld 2006 [158]**, GR | Cross-sectional | 2001 | Rural population | 797 | 9-14 | *Birth order*  Questionnaire | Questionnaire | **≥ 1 wheezing episode (ever)**  NS↑ (≥ 2 vs 1)  **≥ 1 wheezing episode (in last year)**  NS↑ (≥ 2 vs 1)  **≥ 1 wheezing episode and positive SPT (in last year)**  NS↑ (2 vs 1)  NS↓ (≥ 3 vs 1) |

**Column explanations. ^a^** The highest number of subjects in relevant analyses, or – when such numbers were not defined – the number of subjects included in the study. ^b^ Reported in years as either: (a) average ± standard deviation; (b) minimum age-maximum age; or (c) ~approximate age. ^c^ Black bold titles indicate specific outcomes, blue bold titles indicate specific subgroups of subjects, and italic titles indicate analyses of specific exposure. For each analysis, the results are described as (a) NS (non-significant 95% confidence interval); (b) ↑ (significantly increased risk of outcome); or (c) ↓ (significantly decreased risk of outcome). In parenthesis, the sibship size or birth order from which the results are significant is indicated. **Country codes.** AR: Argentina. AT: Austria. AU: Australia. BB: Barbados. BE: Belgium. BO: Bolivia (Plurinational State of). BR: Brazil. BY: Belarus. CA: Canada. CH: Switzerland. CI: Côte d'Ivoire. CL: Chile. CM: Cameroon. CN: China. CO: Colombia. CU: Cuba. DE: Germany. DK: Denmark. DO: Dominican Republic. EC: Ecuador. EE: Estonia. ES: Spain. ET: Ethiopia. FI: Finland. FJ: Fiji. FR: France. GA: Gabon. GB: United Kingdom of Great Britain and Northern Ireland. GR: Greece. HK: Hong Kong. HU: Hungary. ID: Indonesia. IE: Ireland. IL: Israel. IM: Isle of Man. IN: India. IR: Iran (Islamic Republic of). IS: Iceland. IT: Italy. JP: Japan. KG: Kyrgyzstan. KR: Korea, Republic of. KW: Kuwait. LT: Lithuania. MA: Morocco. MG: Madagascar. MK: North Macedonia. MT: Malta. MX: Mexico. MY: Malaysia. NC: New Caledonia. NG: Nigeria. NL: Netherlands. NO: Norway. NZ: New Zealand. OM: Oman. PA: Panama. PE: Peru. PF: French Polynesia. PH: Philippines. PL: Poland. PT: Portugal. SA: Saudi Arabia. SD: Sudan. SE: Sweden. SG: Singapore. SY: Syrian Arab Republic. TH: Thailand. TK: Tokelau. TR: Turkey. TW: Taiwan, Province of China. US: United States of America. UY: Uruguay. VE: Venezuela (Bolivarian Republic of). WS: Samoa. ZA: South Africa. **Other abbreviations.** ARTI: acute respiratory tract infection. ICS: inhaled corticosteroids. N/A: not available. NS: non-significant. OCS: oral corticosteroids. RSV: respiratory syncytial virus. SOB: (attack of) shortness of breath. SPT: skin prick test.

### Table S4C. Table of characteristics for other asthma-related outcomes

| **Study, country** | **Study design** | **Years of data collection** | **Source of study population** | **Subjects analyzed^a^** | **Age (in years)^b^** | **Exposure and assessment method** | **Outcome assessment method** | **Main findings^c^** |
| --- | --- | --- | --- | --- | --- | --- | --- | --- |
| **Brooks 2004 [20]**, JM | Cohort | 1986-1998 | General population | 1,040 | 11-12 | *Birth order*  Medical records | Questionnaire | **Asthma or wheezing (ever)**  NS (no. of older siblings) |
| **Clough 1999 [25]**, GB | Cohort | 1993-1996 | In- and outpatients with newly debuted atopic wheeze and family history of asthma/eczema | 107 | 1-4 | *Birth order*  Interview | Interview | **Asthma medication use (at 12 month follow-up)**  NS (≥ 2 vs 1) |
| **Dahlén 2019 [27]**, SE | Cohort | 2006-2014 | General population | 50,546 | 7-8 | *Birth order, sibship size*  Register data | Register data | **Asthma medication dispensing (in first year of life)**  ↑ (≥ 2 vs 1)  **Asthma medication dispensing (at age 1-6 years)**  ↓ (≥ 2 vs 1)  **Refilled prescription of asthma medication within 18 months**  ↓ (≥ 2 vs 1) |
| **Grabenhenrich 2014 [40]**, DE | Cohort | 1990-2010 | General population | 941 | ~20 | *Birth order*  Questionnaire | Questionnaire | **≥ 2 asthma symptoms (wheezing, SOB, or nocturnal dry cough; in last year)**  NS (≥ 2 vs 1)  **≥ 2 of the following: ≥ 1 asthma symptom in last year, clinician diagnosis ever, and asthma medication use (in last year)**  NS (≥ 2 vs 1)  **Positive sIgE and ≥ 2 of the following: ≥ 1 symptom in last year, clinician diagnosis ever, and asthma medication use (in last year)**  NS (≥ 2 vs 1) |
| **Jarvis 1997 [58]**, GB | Cross-sectional | 1990-1991 | General population | 1,159 | 20-44 | *Birth order, sibship size*  Questionnaire | Questionnaire | **≥ 1 asthma attack (in last year)**  *Birth order*  NS (≥ 2 vs 1)  *Sibship size*  NS (≥ 2 vs 1)  ↓ (per sibling) |
| **Just 2010 [60]**, FR | Cohort | N/A | Children with recurrent wheezing | 219 | ~6 | *Sibship size*  Questionnaire | Questionnaire | **Persistent wheezing or nocturnal cough not associated with infection (in last year)**  ↓ (≥ 4 vs < 4) |
| **Kaplan 1992 [63]**, GB | Cohort | 1969 | General population | 14,188 | ~11 | *Birth order*  N/A | Report from school | **Absence from school for over one week due to asthma or wheezing (in last year)**  NS (≥ 2 vs 1) |
| **Kuschnir 2007 [74]**, BR | Cross-sectional | 2002 | General population | 3,033 | 13-14 | *Birth order*  Questionnaire | Questionnaire | **Aggregated score of asthma-related symptoms (in last year)**  ↓ (≥ 2 vs 1) |
| **Latzin 2007 [79]**, CH | Cohort | 1999-2006 | General population | 195 | ~1 | *Birth order*  Questionnaire | Questionnaire | **Weeks with dyspnea, wheeze, or cough (in first year of life)**  ↑ (≥ 2 vs 1) |
| **Lewis 1996 [84]**, GB | Cohort | 1974, 1986 | General population | 20,528 | ~16 | *Birth order*  Questionnaire | Questionnaire | **Asthma or wheezy bronchitis (in last year)**  NS (≥ 2 vs 1) |
| **Miyake 2011 [97]**, JP | Cross-sectional | 2007-2008 | Pregnant women | 1,745 | N/A (adults) | *Birth order, sibship size*  Questionnaire | Questionnaire | **≥ 1 asthma attack or asthma medication usage (in last year)**  NS (≥ 2 vs 1) |
| **Mommers 2004 [98]**, DE, NL | Nested case-control | 1995, 1997 | **Cases**  Self-reported respiratory symptoms  **Controls**  General population | 775 | 7-8 | *Birth order*  Questionnaire | Questionnaire | **Wheezing and SOB in last year, or daily coughing during autumn/winter and coughing ≥ 3 months (in last year)**  NS (≥ 2 vs 1) |
| **Perzanowski 2008 [115]**, US | Cross-sectional | N/A | Low-income population with high risk of asthma | 1,005 | ~4 | *Birth order*  Questionnaire | Questionnaire | **Asthma medication use (in last 3 months)**  NS (2 vs 1)  ↑ (≥ 3 vs 1) |
| **Rona 1997 [122]**, GB | Cross-sectional | 1990-1991 | General population, with oversampling from socioeconomic disadvantaged areas | 11,924 | 5-11 | *Birth order*  Questionnaire | Questionnaire | **Asthma attack or wheezing (in last year)**  ↓ (≥ 2 vs 1) |
| **Rona 1999 [123]**, GB | Cross-sectional | 1977, 1986, 1994 | General population, with oversampling from socioeconomic disadvantaged areas | 26,959 | 5-11 | *Sibship size*  Questionnaire | Questionnaire | **Asthma attack or wheezing (in last year)**  **All surveys**  ↓ (≥ 3 vs 1)  **1977, 1986 surveys**  ↓ (≥ 4 vs 1)  **1994 survey**  ↓ (≥ 4 vs 1) |
| **Yuan 2003 [156]**, DK | Cohort | 1996-1998 | General population | 9,705 | ~1 | *Birth order*  Register data | Medical records | **Asthma medication prescription (in first year of life)**  ↑ (2-3 vs 1)  NS (≥ 4 vs 1) |

**Column explanations. ^a^** The highest number of subjects in relevant analyses, or – when such numbers were not defined – the number of subjects included in the study. ^b^ Reported in years as either: (a) average ± standard deviation; (b) minimum age-maximum age; or (c) ~approximate age. ^c^ Black bold titles indicate specific outcomes, blue bold titles indicate specific subgroups of subjects, and italic titles indicate analyses of specific exposure. For each analysis, the results are described as (a) NS (non-significant 95% confidence interval); (b) ↑ (significantly increased risk of outcome); or (c) ↓ (significantly decreased risk of outcome). In parenthesis, the sibship size or birth order from which the results are significant is indicated. **Country codes.** BR: Brazil. CH: Switzerland. DE: Germany. DK: Denmark. FR: France. GB: United Kingdom of Great Britain and Northern Ireland. JM: Jamaica. JP: Japan. NL: Netherlands. SE: Sweden. US: United States of America. **Other abbreviations.** N/A: not available. NS: non-significant. sIgE: allergen-specific immunoglobulin E. SOB: (attack of) shortness of breath.

## Table S5. Quality appraisal

| **Study** | **Selection bias** | **Study design** | **Confounders** | **Blinding** | **Data collection methods** | **Withdrawals and drop-outs** | **Overall rating** |
| --- | --- | --- | --- | --- | --- | --- | --- |
| **Aaron 1967 [1]** | Moderate | Moderate | Weak | Moderate | Strong | Moderate | **Moderate** |
| **Addo-Yobo 2001 [2]** | Moderate | Moderate | Strong | Moderate | Strong | Moderate | **Strong** |
| **Adler 2005 [3]** | Weak | Moderate | Moderate | Moderate | Moderate | Moderate | **Moderate** |
| **Akcay 2014 [4]** | Strong | Moderate | Strong | Moderate | Moderate | Moderate | **Strong** |
| **Almqvist 2016 [5]** | Strong | Strong | Moderate | Strong | Strong | Moderate | **Strong** |
| **Attena 1999 [6]** | Moderate | Moderate | Weak | Moderate | Strong | Moderate | **Moderate** |
| **Ayatollahi 2006 [7]** | Weak | Moderate | Weak | Moderate | Strong | Moderate | **Weak** |
| **Ayuk 2018 [8]** | Strong | Moderate | Strong | Moderate | Moderate | Moderate | **Strong** |
| **Bager 2003 [9]** | Strong | Strong | Weak | Moderate | Strong | Moderate | **Moderate** |
| **Baker 1998 [10]** | Moderate | Strong | Weak | Moderate | Moderate | Weak | **Weak** |
| **Ball 2000 [11]** | Moderate | Strong | Moderate | Moderate | Strong | Strong | **Strong** |
| **Batllés-Garrido 2010 [12]** | Weak | Moderate | Strong | Moderate | Strong | Moderate | **Moderate** |
| **Bedolla-Barajas 2018 [13]** | Moderate | Moderate | Moderate | Moderate | Moderate | Moderate | **Strong** |
| **Bercedo-Sanz 2015 [14]** | Weak | Moderate | Moderate | Moderate | Moderate | Moderate | **Moderate** |
| **Bernsen 2003 [15]** | Moderate | Strong | Moderate | Moderate | Strong | Moderate | **Strong** |
| **Bodner 1998 [16]** | Moderate | Moderate | Weak | Moderate | Strong | Moderate | **Moderate** |
| **Bodner 2000 [17]** | Moderate | Strong | Weak | Moderate | Strong | Moderate | **Moderate** |
| **Boneberger 2011 [18]** | Moderate | Moderate | Strong | Moderate | Moderate | Moderate | **Strong** |
| **Bråbäck 1998 [19]** | Strong | Strong | Weak | Strong | Strong | Moderate | **Moderate** |
| **Brooks 2004 [20]** | Moderate | Strong | Strong | Moderate | Moderate | Moderate | **Strong** |
| **Burr 1997 [21]** | Moderate | Strong | Moderate | Moderate | Strong | Strong | **Strong** |
| **Campo 2006 [22]** | Moderate | Strong | Weak | Moderate | Moderate | Weak | **Weak** |
| **Chunmei 2014 [23]** | Moderate | Moderate | Weak | Moderate | Weak | Moderate | **Weak** |
| **Cifuentes 2003 [24]** | Moderate | Strong | Strong | Weak | Strong | Strong | **Moderate** |
| **Clough 1999 [25]** | Moderate | Strong | Weak | Weak | Strong | Strong | **Weak** |
| **Cooper 2014 [26]** | Moderate | Moderate | Moderate | Moderate | Strong | Moderate | **Strong** |
| **Dahlén 2019 [27]** | Moderate | Strong | Weak | Moderate | Strong | Strong | **Moderate** |
| **Davis 1981 [28]** | Moderate | Moderate | Weak | Moderate | Strong | Moderate | **Moderate** |
| **De Meer 2005 [29]** | Weak | Moderate | Moderate | Moderate | Strong | Moderate | **Moderate** |
| **Dik 2004 [30]** | Strong | Strong | Weak | Strong | Strong | Moderate | **Moderate** |
| **Elder 1996 [31]** | Moderate | Strong | Moderate | Moderate | Weak | Moderate | **Moderate** |
| **Farooqi 1998 [32]** | Moderate | Strong | Weak | Strong | Strong | Moderate | **Moderate** |
| **Foliaki 2008 [33]** | Weak | Moderate | Moderate | Moderate | Strong | Weak | **Weak** |
| **Freitas 2012 [34]** | Moderate | Weak | Moderate | Moderate | Strong | Moderate | **Moderate** |
| **Furuhata 2020 [35]** | Strong | Strong | Strong | Moderate | Strong | Weak | **Moderate** |
| **Gao 2005 [36]** | Weak | Strong | Weak | Moderate | Strong | Moderate | **Weak** |
| **Gao 2021 [37]** | Moderate | Strong | Moderate | Moderate | Moderate | Moderate | **Strong** |
| **Goldberg 2007 [38]** | Strong | Strong | Weak | Strong | Strong | Moderate | **Moderate** |
| **Goldstein 2005 [39]** | Weak | Strong | Weak | Moderate | Strong | Weak | **Weak** |
| **Grabenhenrich 2014 [40]** | Strong | Strong | Strong | Moderate | Strong | Moderate | **Strong** |
| **Gray 2019 [41]** | Weak | Strong | Strong | Moderate | Moderate | Moderate | **Moderate** |
| **Greenough 2005 [42]** | Strong | Strong | Weak | Moderate | Moderate | Moderate | **Moderate** |
| **Gupta 2016 [43]** | Weak | Moderate | Moderate | Moderate | Strong | Moderate | **Moderate** |
| **Gustafsson 1996 [44]** | Moderate | Strong | Moderate | Moderate | Moderate | Moderate | **Strong** |
| **Haby 2001 [45]** | Moderate | Moderate | Moderate | Moderate | Strong | Moderate | **Strong** |
| **Hallit 2018 [46]** | Strong | Strong | Strong | Moderate | Moderate | Moderate | **Strong** |
| **Hedman 2015 [47]** | Strong | Strong | Moderate | Moderate | Strong | Weak | **Moderate** |
| **Held 2000 [48]** | Weak | Strong | Weak | Moderate | Strong | Strong | **Weak** |
| **Herr 2012 [49]** | Moderate | Strong | Moderate | Moderate | Strong | Weak | **Moderate** |
| **Hessel 2001 [50]** | Moderate | Strong | Weak | Moderate | Moderate | Weak | **Weak** |
| **Higgins 2021 [51]** | Moderate | Strong | Moderate | Moderate | Moderate | Weak | **Moderate** |
| **Hijazi 2000 [52]** | Weak | Moderate | Moderate | Moderate | Strong | Moderate | **Moderate** |
| **Hosoki 2009 [53]** | Moderate | Strong | Moderate | Moderate | Weak | Weak | **Weak** |
| **Hossain 2020 [54]** | Weak | Moderate | Weak | Moderate | Strong | Moderate | **Weak** |
| **Huang 2018 [55]** | Strong | Strong | Strong | Moderate | Strong | Strong | **Strong** |
| **Hughes 2013 [56]** | Weak | Moderate | Moderate | Moderate | Strong | Moderate | **Moderate** |
| **Infante-Rivard 2009 [57]** | Weak | Strong | Moderate | Moderate | Strong | Moderate | **Moderate** |
| **Jarvis 1997 [58]** | Moderate | Moderate | Moderate | Moderate | Moderate | Weak | **Moderate** |
| **Jedrychowski 2009 [59]** | Moderate | Strong | Moderate | Moderate | Strong | Weak | **Moderate** |
| **Just 2010 [60]** | Moderate | Strong | Strong | Moderate | Strong | Weak | **Moderate** |
| **Kansen 2020 [61]** | Weak | Moderate | Strong | Moderate | Moderate | Moderate | **Moderate** |
| **Kaplan 1989 [62]** | Strong | Strong | Weak | Moderate | Strong | Strong | **Moderate** |
| **Kaplan 1992 [63]** | Moderate | Strong | Weak | Moderate | Strong | Weak | **Weak** |
| **Karunanayake 2020 [64]** | Weak | Moderate | Moderate | Moderate | Moderate | Moderate | **Moderate** |
| **Kearney 1998 [65]** | Strong | Moderate | Weak | Moderate | Strong | Moderate | **Moderate** |
| **Kiechl-Kohlendorfer 2007 [66]** | Moderate | Strong | Moderate | Moderate | Strong | Weak | **Moderate** |
| **Kikkawa 2018 [67]** | Strong | Strong | Strong | Moderate | Moderate | Moderate | **Strong** |
| **Kilpeläinen 2000 [68]** | Moderate | Moderate | Strong | Moderate | Strong | Moderate | **Strong** |
| **Kim 2012 [69]** | Moderate | Moderate | Weak | Moderate | Strong | Moderate | **Moderate** |
| **Kingsley 1949 [70]** | Weak | Strong | Weak | Moderate | Strong | Moderate | **Weak** |
| **Kinra 2006 [71]** | Moderate | Moderate | Moderate | Weak | Moderate | Moderate | **Moderate** |
| **Kramer 2009 [72]** | Moderate | Strong | Strong | Moderate | Strong | Weak | **Moderate** |
| **Krzych-Fałta 2018 [73]** | Strong | Moderate | Weak | Moderate | Moderate | Moderate | **Moderate** |
| **Kuschnir 2007 [74]** | Moderate | Moderate | Strong | Moderate | Moderate | Moderate | **Strong** |
| **Kusunoki 2012 [75]** | Moderate | Moderate | Strong | Moderate | Moderate | Moderate | **Strong** |
| **Kutzora 2018 [76]** | Weak | Moderate | Moderate | Moderate | Moderate | Moderate | **Moderate** |
| **Larenas-Linnemann 2020 [77]** | Moderate | Moderate | Moderate | Weak | Strong | Moderate | **Moderate** |
| **Larsson 2008 [78]** | Moderate | Strong | Strong | Moderate | Moderate | Moderate | **Strong** |
| **Latzin 2007 [79]** | Moderate | Strong | Strong | Moderate | Moderate | Weak | **Moderate** |
| **Lee 2004 [80]** | Moderate | Strong | Moderate | Moderate | Strong | Moderate | **Strong** |
| **Lee 2012 [81]** | Moderate | Moderate | Moderate | Moderate | Strong | Moderate | **Strong** |
| **Lemanske 2005 [82]** | Moderate | Strong | Strong | Moderate | Weak | Strong | **Moderate** |
| **Lewis 1995 [83]** | Strong | Strong | Strong | Moderate | Weak | Weak | **Weak** |
| **Lewis 1996 [84]** | Strong | Strong | Strong | Moderate | Moderate | Weak | **Moderate** |
| **Lewis 1998 [85]** | Weak | Strong | Strong | Moderate | Moderate | Weak | **Weak** |
| **Lima 2010 [86]** | Moderate | Moderate | Moderate | Weak | Strong | Moderate | **Moderate** |
| **Lin 2019 [87]** | Strong | Moderate | Strong | Strong | Strong | Moderate | **Strong** |
| **Linneberg 2006 [88]** | Moderate | Strong | Moderate | Weak | Weak | Strong | **Weak** |
| **Loss 2016 [89]** | Moderate | Strong | Moderate | Moderate | Moderate | Weak | **Moderate** |
| **Martins 2015 [90]** | Moderate | Strong | Moderate | Moderate | Strong | Moderate | **Strong** |
| **McCandless 1970 [91]** | Moderate | Moderate | Weak | Moderate | Moderate | Moderate | **Moderate** |
| **McKeever 2001 [92]** | Strong | Strong | Weak | Moderate | Strong | Strong | **Moderate** |
| **McLeod 2020 [93]** | Moderate | Moderate | Moderate | Moderate | Moderate | Moderate | **Strong** |
| **Mejias 2018 [94]** | Moderate | Moderate | Moderate | Moderate | Moderate | Moderate | **Strong** |
| **Midodzi 2007 [95]** | Strong | Strong | Strong | Moderate | Moderate | Strong | **Strong** |
| **Miyake 2004 [96]** | Moderate | Moderate | Strong | Moderate | Moderate | Moderate | **Strong** |
| **Miyake 2011 [97]** | Moderate | Moderate | Strong | Moderate | Moderate | Moderate | **Strong** |
| **Mommers 2004 [98]** | Moderate | Strong | Strong | Moderate | Moderate | Moderate | **Strong** |
| **Moncayo 2010 [99]** | Strong | Moderate | Moderate | Moderate | Moderate | Moderate | **Strong** |
| **Moraes 2013 [100]** | Strong | Moderate | Weak | Moderate | Moderate | Moderate | **Moderate** |
| **Morata-Alba 2019 [101]** | Moderate | Strong | Weak | Moderate | Strong | Moderate | **Moderate** |
| **Nafstad 2000 [102]** | Strong | Strong | Strong | Moderate | Moderate | Strong | **Strong** |
| **Nafstad 2005 [103]** | Strong | Strong | Strong | Moderate | Moderate | Strong | **Strong** |
| **Nicolai 2017 [104]** | Moderate | Strong | Moderate | Moderate | Weak | Weak | **Weak** |
| **Nicolaou 2008 [105]** | Moderate | Strong | Weak | Moderate | Strong | Strong | **Moderate** |
| **Nowak 1996 [106]** | Moderate | Moderate | Weak | Moderate | Strong | Moderate | **Moderate** |
| **Ohfuji 2009 [107]** | Moderate | Moderate | Moderate | Moderate | Moderate | Moderate | **Strong** |
| **Oliveira-Santos 2015 [108]** | Moderate | Moderate | Moderate | Moderate | Moderate | Moderate | **Strong** |
| **Oluwole 2013 [109]** | Weak | Moderate | Weak | Moderate | Moderate | Moderate | **Weak** |
| **Özmert 2009 [110]** | Weak | Moderate | Moderate | Moderate | Strong | Moderate | **Moderate** |
| **Parthasarathi 2021 [111]** | Moderate | Moderate | Strong | Moderate | Moderate | Moderate | **Strong** |
| **Pekkanen 1999 [112]** | Moderate | Moderate | Weak | Moderate | Strong | Moderate | **Moderate** |
| **Pekkanen 2001 [113]** | Moderate | Strong | Strong | Moderate | Strong | Weak | **Moderate** |
| **Pérez Tarazona 2010 [114]** | Moderate | Strong | Moderate | Moderate | Strong | Strong | **Strong** |
| **Perzanowski 2008 [115]** | Weak | Moderate | Weak | Moderate | Strong | Moderate | **Weak** |
| **Ponsonby 1998 [116]** | Strong | Moderate | Weak | Moderate | Moderate | Moderate | **Moderate** |
| **Ponsonby 1999 [117]** | Moderate | Strong | Weak | Moderate | Moderate | Moderate | **Moderate** |
| **Ponsonby 2003 [118]** | Moderate | Strong | Moderate | Moderate | Strong | Strong | **Strong** |
| **Räsänen 1997 [119]** | Strong | Moderate | Weak | Moderate | Strong | Moderate | **Moderate** |
| **Räsänen 2000 [120]** | Strong | Moderate | Moderate | Moderate | Strong | Moderate | **Strong** |
| **Rehalia 2020 [121]** | Weak | Moderate | Weak | Moderate | Strong | Moderate | **Weak** |
| **Rona 1997 [122]** | Moderate | Moderate | Weak | Moderate | Moderate | Moderate | **Moderate** |
| **Rona 1999 [123]** | Strong | Moderate | Weak | Moderate | Moderate | Moderate | **Moderate** |
| **Rönmark 2009 [124]** | Moderate | Strong | Moderate | Moderate | Strong | Strong | **Strong** |
| **Rönmark 2016 [125]** | Strong | Moderate | Moderate | Moderate | Moderate | Moderate | **Strong** |
| **Rusconi 1999 [126]** | Strong | Moderate | Strong | Moderate | Moderate | Moderate | **Strong** |
| **Rusconi 2005 [127]** | Weak | Moderate | Strong | Moderate | Strong | Moderate | **Moderate** |
| **Rutter 2020 [128]** | Strong | Moderate | Strong | Moderate | Moderate | Moderate | **Strong** |
| **Salam 2004 [129]** | Moderate | Strong | Weak | Moderate | Strong | Moderate | **Moderate** |
| **Saya 2012 [130]** | Moderate | Moderate | Moderate | Moderate | Strong | Moderate | **Strong** |
| **Saya 2014 [131]** | Moderate | Moderate | Weak | Moderate | Moderate | Moderate | **Moderate** |
| **Schmitz 2012 [132]** | Strong | Moderate | Moderate | Moderate | Strong | Moderate | **Strong** |
| **Sears 1996 [133]** | Moderate | Strong | Weak | Moderate | Strong | Strong | **Moderate** |
| **Sheldrake 1976a [134]** | Moderate | Moderate | Weak | Moderate | Weak | Moderate | **Weak** |
| **Sheldrake 1976b [135]** | Moderate | Moderate | Weak | Moderate | Moderate | Moderate | **Moderate** |
| **Sherriff 2001 [136]** | Moderate | Strong | Strong | Moderate | Moderate | Weak | **Moderate** |
| **Shohat 2002 [137]** | Strong | Moderate | Moderate | Moderate | Moderate | Moderate | **Strong** |
| **Simões 2019 [138]** | Moderate | Moderate | Strong | Moderate | Strong | Moderate | **Strong** |
| **Slob 2020 [139]** | Weak | Strong | Weak | Moderate | Strong | Weak | **Weak** |
| **Smith 1960 [140]** | Weak | Strong | Moderate | Moderate | Moderate | Weak | **Weak** |
| **Strachan 2015 [141]** | Strong | Moderate | Weak | Moderate | Moderate | Moderate | **Moderate** |
| **Stråvik 2020 [142]** | Moderate | Strong | Weak | Moderate | Strong | Strong | **Moderate** |
| **Sugiura 2021 [143]** | Moderate | Moderate | Moderate | Moderate | Strong | Moderate | **Strong** |
| **Sunyer 1997 [144]** | Moderate | Moderate | Moderate | Moderate | Strong | Weak | **Moderate** |
| **Svanes 2002 [145]** | Strong | Moderate | Weak | Moderate | Moderate | Moderate | **Moderate** |
| **Teijeiro 2017 [146]** | Moderate | Moderate | Strong | Moderate | Moderate | Moderate | **Strong** |
| **Toppila-Salmi 2019 [147]** | Moderate | Moderate | Weak | Strong | Strong | Moderate | **Moderate** |
| **Toppila-Salmi 2021 [148]** | Strong | Moderate | Strong | Moderate | Strong | Moderate | **Strong** |
| **Van Beijsterveldt 2008 [149]** | Moderate | Strong | Weak | Moderate | Strong | Strong | **Moderate** |
| **Venero-Fernández 2013 [150]** | Moderate | Moderate | Moderate | Moderate | Moderate | Moderate | **Strong** |
| **Venter 2021 [151]** | Moderate | Strong | Strong | Moderate | Strong | Moderate | **Strong** |
| **Victorino 2009 [152]** | Strong | Moderate | Moderate | Moderate | Strong | Moderate | **Strong** |
| **Westergaard 2005 [153]** | Moderate | Moderate | Weak | Moderate | Strong | Moderate | **Moderate** |
| **Wickens 1999 [154]** | Moderate | Moderate | Weak | Moderate | Strong | Moderate | **Moderate** |
| **Wolff 2012 [155]** | Moderate | Moderate | Weak | Moderate | Moderate | Moderate | **Moderate** |
| **Yuan 2003 [156]** | Strong | Strong | Weak | Strong | Strong | Moderate | **Moderate** |
| **Zadnick 2010 [157]** | Strong | Moderate | Moderate | Moderate | Moderate | Moderate | **Strong** |
| **Zekveld 2006 [158]** | Moderate | Moderate | Weak | Moderate | Strong | Moderate | **Moderate** |

## Table S6. Publication bias

| **A. Any wheezing – birth order ≥ 2 vs 1** | | | | | |
| --- | --- | --- | --- | --- | --- |
| **Kendall's tau (Begg and Mazumdar’s test)** | ***p* (Begg and Mazumdar’s test)** | **z-value (Egger's test)** | ***p* (Egger's test)** | **Missing (trim-and-fill)** | **Side (trim-and-fill)** |
| 0.109497883 | 0.228381395 | 0.668749515 | 0.5 | 0 | Left |
| **B. Any wheezing – sibship size ≥ 2 vs 1** | | | | | |
| **Kendall's tau (Begg and Mazumdar’s test)** | ***p* (Begg and Mazumdar’s test)** | **z-value (Egger's test)** | ***p* (Egger's test)** | **Missing (trim-and-fill)** | **Side (trim-and-fill)** |
| 0.172906 | 0.155761 | 2.871291 | 0.004 | 0 | Left |
| **C. Current asthma – birth order ≥ 2 vs 1** | | | | | |
| **Kendall's tau (Begg and Mazumdar’s test)** | ***p* (Begg and Mazumdar’s test)** | **z-value (Egger's test)** | ***p* (Egger's test)** | **Missing (trim-and-fill)** | **Side (trim-and-fill)** |
| -0.08103 | 0.332685 | -3.09046 | 0.002 | 21 | Right |
| **D. Current asthma – sibship size ≥ 2 vs 1** | | | | | |
| **Kendall's tau (Begg and Mazumdar’s test)** | ***p* (Begg and Mazumdar’s test)** | **z-value (Egger's test)** | ***p* (Egger's test)** | **Missing (trim-and-fill)** | **Side (trim-and-fill)** |
| 0.086771 | 0.453278 | -0.49102 | 0.62 | 5 | Right |
| **E. Ever asthma – birth order ≥ 2 vs 1** | | | | | |
| **Kendall's tau (Begg and Mazumdar’s test)** | ***p* (Begg and Mazumdar’s test)** | **z-value (Egger's test)** | ***p* (Egger's test)** | **Missing (trim-and-fill)** | **Side (trim-and-fill)** |
| 0.162907 | 0.074512 | 0.441436 | 0.66 | 1 | left |

For each exposure-outcome pair with ≥ 10 studies (A-E), results are presented from Begg and Mazumdar’s (rank correlation) test (column 1-2), Egger’s (regression) test (column 3-4), and from the trim-and-fill function (column 5-6). Missing (trim-and-fill): the estimated number of missing studies in plots that are deemed asymmetric by the function (k0 from the *trimfill()* function). Side (trim-and-fill): indicates which side on which the missing values are located (side from the *trimfill()* function).

## Table S7. Sensitivity analysis (subgroups)

| **A. Recurrent wheezing – Sibship size** | | | | |
| --- | --- | --- | --- | --- |
| **Selection** | **No. of studies** | **Risk ratio (95% CI)** | ***I²*** | **τ²** |
| All studies | 3 | 1.43 (0.71-2.9) | 72.69 | 0.08 |
| Moderate and strong quality | 2 | 1.55 (0.04-62.04) | 84 | 0.2 |
| **B. Current asthma – Birth order** | | | | |
| **Selection** | **No. of studies** | **Risk ratio (95% CI)** | ***I²*** | **τ²** |
| All studies | 23 | 0.94 (0.87-1.01) | 71.4 | 0.02 |
| Moderate and strong quality | 21 | 0.93 (0.86-1.01) | 73.88 | 0.02 |
| Clinically confirmed outcome | 11 | 0.95 (0.84-1.07) | 63.32 | 0.01 |
| **C. Current asthma – Sibship size** | | | | |
| **Selection** | **No. of studies** | **Risk ratio (95% CI)** | ***I²*** | **τ²** |
| All studies | 13 | 0.97 (0.82-1.14) | 89.27 | 0.06 |
| Moderate and strong quality | 12 | 0.97 (0.82-1.16) | 89.83 | 0.06 |
| Clinically confirmed outcome | 3 | 0.83 (0.48-1.43) | 23.03 | 0.01 |
| **D. Ever asthma - Birth order** | | | | |
| **Selection** | **No. of studies** | **Risk ratio (95% CI)** | ***I²*** | **τ²** |
| All studies | 19 | 0.94 (0.88-1.01) | 71.84 | 0.01 |
| Moderate and strong quality | 17 | 0.93 (0.85-1.03) | 72.08 | 0.02 |
| Clinically confirmed outcome | 5 | 0.92 (0.71-1.21) | 86.13 | 0.02 |
| **E. Ever asthma - Sibship size** | | | | |
| **Selection** | **No. of studies** | **Risk ratio (95% CI)** | ***I²*** | **τ²** |
| All studies | 7 | 0.95 (0.84-1.08) | 66.35 | 0 |
| Moderate and strong quality | 7 | 0.95 (0.84-1.08) | 66.35 | 0 |
| Clinically confirmed outcome | 2 | 0.82 (0.01-54.62) | 25.02 | 0.42 |

For each exposure-outcome pair where there were ≥ 2 studies to perform at least one sensitivity analysis, results are presented on the number of studies, risk ratio with 95% confidence interval, as well as *I^2^* and τ². Moderate and strong quality: overall rating of study with the Effective Public Health Practice Project (EPHPP) tool. Clinically confirmed outcome: outcome assessed by medical records or clinical examination.

## Table S8. Sensitivity analysis (rho)

| **A. Any wheezing - Birth order** | | | | | | | |
| --- | --- | --- | --- | --- | --- | --- | --- |
|  |  | **Rho = 0** | **Rho = 0.2** | **Rho = 0.4** | **Rho = 0.6** | **Rho = 0.8** | **Rho = 1** |
| X.Intercept. | Coefficient | 0.1479 | 0.1479 | 0.1479 | 0.1479 | 0.1479 | 0.1479 |
|  | Std. Error | 0.0507 | 0.0507 | 0.0507 | 0.0507 | 0.0507 | 0.0507 |
| Tau.sq | Estimate | 0.0342 | 0.0342 | 0.0342 | 0.0342 | 0.0342 | 0.0342 |
| **B. Any wheezing - Sibship size** | | | | | | | |
|  |  | **Rho = 0** | **Rho = 0.2** | **Rho = 0.4** | **Rho = 0.6** | **Rho = 0.8** | **Rho = 1** |
| X.Intercept. | Coefficient | 0.09792 | 0.09796 | 0.09799 | 0.0980 | 0.09807 | 0.09810 |
|  | Std. Error | 0.03567 | 0.03568 | 0.03569 | 0.0357 | 0.03571 | 0.03572 |
| Tau.sq | Estimate | 0.00805 | 0.00807 | 0.00808 | 0.0081 | 0.00811 | 0.00813 |
| **C. Recurrent wheezing - Birth order** | | | | | | | |
|  |  | **Rho = 0** | **Rho = 0.2** | **Rho = 0.4** | **Rho = 0.6** | **Rho = 0.8** | **Rho = 1** |
| X.Intercept. | Coefficient | 0.47 | 0.47 | 0.471 | 0.471 | 0.471 | 0.471 |
|  | Std. Error | 0.197 | 0.197 | 0.197 | 0.197 | 0.197 | 0.197 |
| Tau.sq | Estimate | 0.13 | 0.13 | 0.131 | 0.132 | 0.132 | 0.133 |
| **D. Recurrent wheezing - Sibship size** | | | | | | | |
|  |  | **Rho = 0** | **Rho = 0.2** | **Rho = 0.4** | **Rho = 0.6** | **Rho = 0.8** | **Rho = 1** |
| X.Intercept. | Coefficient | 0.3591 | 0.3596 | 0.36 | 0.3605 | 0.361 | 0.3613 |
|  | Std. Error | 0.1599 | 0.1602 | 0.1605 | 0.1608 | 0.161 | 0.1613 |
| Tau.sq | Estimate | 0.0696 | 0.0712 | 0.0728 | 0.0744 | 0.076 | 0.0776 |
| **E. Current asthma – Birth order** | | | | | | | |
|  |  | **Rho = 0** | **Rho = 0.2** | **Rho = 0.4** | **Rho = 0.6** | **Rho = 0.8** | **Rho = 1** |
| X.Intercept. | Coefficient | -0.0666 | -0.0666 | -0.0666 | -0.0666 | -0.0666 | -0.0667 |
|  | Std. Error | 0.0360 | 0.0360 | 0.0360 | 0.0361 | 0.0361 | 0.0361 |
| Tau.sq | Estimate | 0.0170 | 0.0170 | 0.0170 | 0.0170 | 0.0170 | 0.0170 |
| **F. Current asthma – Sibship size** | | | | | | | |
|  |  | **Rho = 0** | **Rho = 0.2** | **Rho = 0.4** | **Rho = 0.6** | **Rho = 0.8** | **Rho = 1** |
| X.Intercept. | Coefficient | -0.0343 | -0.0343 | -0.0343 | -0.0343 | -0.0343 | -0.0343 |
|  | Std. Error | 0.0739 | 0.0739 | 0.0739 | 0.0739 | 0.0739 | 0.0739 |
| Tau.sq | Estimate | 0.0587 | 0.0587 | 0.0588 | 0.0588 | 0.0589 | 0.0589 |
| **G. Ever asthma – Birth order** | | | | | | | |
|  |  | **Rho = 0** | **Rho = 0.2** | **Rho = 0.4** | **Rho = 0.6** | **Rho = 0.8** | **Rho = 1** |
| X.Intercept. | Coefficient | -0.06132 | -0.06133 | -0.06133 | -0.06134 | -0.06135 | -0.06135 |
|  | Std. Error | 0.03251 | 0.03251 | 0.03251 | 0.03251 | 0.03252 | 0.03252 |
| Tau.sq | Estimate | 0.00782 | 0.00783 | 0.00784 | 0.00784 | 0.00785 | 0.00785 |
| **H. Ever asthma – Sibship size** | | | | | | | |
|  |  | **Rho = 0** | **Rho = 0.2** | **Rho = 0.4** | **Rho = 0.6** | **Rho = 0.8** | **Rho = 1** |
| X.Intercept. | Coefficient | -0.04746 | -0.04747 | -0.04749 | -0.0475 | -0.04751 | -0.04752 |
|  | Std. Error | 0.03414 | 0.03422 | 0.03430 | 0.03438 | 0.03446 | 0.03454 |
| Tau.sq | Estimate | 0.00261 | 0.00263 | 0.00265 | 0.00267 | 0.00269 | 0.00271 |

For every exposure-outcome pair, presented are results from sensitivity analysis of the meta-analysis, based on the rho parameter value. With increments of 0.2 from 0 to 1, the meta-analysis was re-run and the coefficient, standard error (Std. Error), as well as tau-squared (Tau.sq) were calculated.

## Excluded studies

### Table S9A. Excluded studies (initial search)

| **No abstract or full-text (reports not retrieved)** | 1. Survey on the etiology of infantile asthma in Spain. An Esp Pediatr. 1982;17(5):411-434. 2. The development of lung function in Sydney children: effects of respiratory illness and smoking. A ten year study. Eur J Respir Dis Suppl. 1984;132:1-137. 3. Risk factors for preschool asthma. Medicine Today. 2001;2(9):14-15. 4. Family size, not birth order, affects asthma prevalence. Patient Care. 2007;41(18):1-1. 5. Aberle N, Kljaic Bukvic B, Blekic M, et al. Risk factors for childhood asthma in Croatia. Allergy: European Journal of Allergy and Clinical Immunology. 2010;65:314. 29th Congress of the European Academy of Allergy and Clinical Immunology, EAACI. London United Kingdom. 6. Akanbi MO, Ozoh OB, Akanbi FO, Ukoli CO. Self-reported asthma symptoms among medical undergraduates in North Central Nigeria. American Journal of Respiratory and Critical Care Medicine. 2013;187American Thoracic Society International Conference, ATS 2013. Philadelphia, PA United States. 7. Alba F, Flecha E, Alba J, Hernández R, Busquets E. Characteristics of infantile asthma in patients seen in primary care. Aten Primaria. 1996;18(2):83-86. 8. Alba Moreno F, Alsina Donadeu J. Clinical epidemiological study of lower respiratory tract illness with wheezing in children under 2 years of age and its risk factors. An Esp Pediatr. 1999;50(4):379-383. 9. Apfelbacher CJ, Diepgen TL, Schmitt J. Epidemiology of eczema - A representative German cross-sectional study. Experimental Dermatology. 2010;19(2):218. 37th Annual Meeting of the Arbeitsgemeinschaft Dermatologische Forschung, ADF. Luebeck Germany. 10. Asani S, Vlaski E, Jakupi T, Nikolovski L, Tomic V. Household size and allergic diseases in childhood. Acta Paediatrica. 2008;97:176-176. 11. Asch AJ, Rabin DL, Hurwitz A, Medalie JH. Bronchial asthma in an Israel community. A community survey using primary care resources. Isr J Med Sci. 1973;9(8):1014-1021. 12. Asmussen L, Arduino KE, Olson LM, Grant EN, Weiss KB. Family characteristics matter: Differences in the children's health survey for asthma (CHSA) from PAC PORT II. Journal of Allergy and Clinical Immunology. 2001;107(2):S246-S246. 13. Bak E, Van Ginkel CD, Kollen BJ, Van Der Heide S, Flokstra-De Blok BMJ, Dubois AE. Birth order and food allergy: First born children are at greater risk for sensitisation to foods than subsequently born children. Allergy: European Journal of Allergy and Clinical Immunology. 2014;69:381. 33rd Congress of the European Academy of Allergy and Clinical Immunology. Copenhagen Denmark. 14. Ball TM, Remes S, Holberg CJ, Martinez FD, Wright AL. The effect of siblings and day care attendance on the development of allergic sensitization in childhood. Allergy. 2002;57:29-30. 15. Bateman BJ. The behaviour of three year olds in relation to allergy and exposure to artificial additives. University of Southampton (United Kingdom); 2004. https://search.proquest.com/docview/305036611 16. Bendel J, Palti H, Winter S, Ornoy A. Prevalence of disabilities in a national sample of 3-year-old Israeli children. Isr J Med Sci. 1989;25(5):264-270. 17. Bennis A, el Fassy Fihry MT, Fikri-Benbrahim N, Sayah-Moussaoui Z, Samir-Rafi A, Biaz A. The prevalence of adolescent asthma in Rabat. A survey conducted in secondary schools. Rev Mal Respir. 1992;9(2):163-169. 18. Bhat BR, Friedman S, Adimoolam S, Schneider AT, Chiaramonte LT. Study of social, educational, environmental and cultural aspects of childhood asthma in clinic and private patients in the city of New York. Ann Allergy. 1978;41(2):89-92. 19. Bishara HJ, Zidani S, Sliman M. Prevalence of asthma and related symptoms among Galilee Bedouin school children. Chest. 1996;110(4) 20. Bittera I, Kadocsa E, Roman R, Turi S. The changing prevalence of allergic rhinitis among school-children in Szeged in the past 20 years (1987-2007). Allergy: European Journal of Allergy and Clinical Immunology. 2009;64:212. 28th Congress of the European Academy of Allergy and Clinical Immunology Abstract Book. Warszawa Poland. 21. Bjarnadóttir E, Gíslason D, Gíslason T. Atopy and allergic disorders among Icelandic medical students. Laeknabladid. 2001;87(7):621-624. 22. Blumberg MZ, McComb N. Incidence of food allergy and birth order. Annals of Allergy, Asthma and Immunology. 2013;111(5):A100. 2013 Annual Meeting of the American College of Allergy, Asthma and Immunology. Baltimore, MD United States. 23. Botezan MC. The “sibling effect” in children at high risk for atopic disorders. Michigan State University; 2000. https://search.proquest.com/docview/230832849 24. Braun-Fahrlander C, Wuthrich B, Gassner M, et al. Prevalence and risk factors of allergic sensitization in Swiss school children. Allergologie. 1999;22(1):54-64. Pravalenz und risikofaktoren einer allergischen sensibilisierung bei schulkindern in der Schweiz. 25. Bremner SA. Exploring early-life risk factors for childhood hay fever in two primary care databases. University of London, St. George's Hospital Medical School (United Kingdom); 2007. https://search.proquest.com/docview/301660727. 26. Buser K, Werner S, Volk P. Illness and social status--the special case of neurodermatitis. Gesundheitswesen. 1998;60(5):311-316. 27. Cangiano G, Pierangeli A, Scagnolari C, et al. Risk factors for recurrent wheezing following bronchiolitis: 3 years of follow-up. American Journal of Respiratory and Critical Care Medicine. 2012;185American Thoracic Society International Conference, ATS 2012. San Francisco, CA United States. 28. Cantwell RJ. A prospective study of Maori infant health and the problem of nutritional anaemia. N Z Med J. 1973;78(495):61-65. 29. Cardenas R, Garcia C, Garcia R, et al. Epidemiological study in child population of the central area of Spain. Allergy: European Journal of Allergy and Clinical Immunology. 2014;69:537-538. 33rd Congress of the European Academy of Allergy and Clinical Immunology. Copenhagen Denmark. 30. Castro-Rodriguez JA. Predictors of asthma in young children in lowand middle income countries. Pediatric Pulmonology. 2014;49:S3-S4. 13th International Congress on Pediatric Pulmonology. Bruges Belgium. 31. Caudri D, Savenije O, Smit HA, et al. The relation between perinatal factors and phenotypes of wheeze in the first 8 years of life. American Journal of Respiratory and Critical Care Medicine. 2010;181(1)American Thoracic Society International Conference, ATS 2010. New Orleans, LA United States. 32. Ceuppens J. Western lifestyle, local defenses and the rising incidence of allergic rhinitis. Acta Otorhinolaryngol Belg. 2000;54(3):391-395. 33. Christie GL, McDougall CM, Helms PJ. Is the increase in asthma prevalence occurring in children without a family history of atopy? Scott Med J. 1998;43(6):180-182. 34. Clough JB, Warner JA, Warner JO. Prediction of persistent wheeze in infants. Thorax. 1997;52 35. Cortès X, Soriano JB, Sunyer J, et al. Factors associated with the development of atopy in young adults. Med Clin (Barc). 2000;114(5):165-168. 36. Coulie P. Reasons for the increase in the prevalence of allergies. Allergy and Clinical Immunology International. 2002;14(6):288-289. 37. Cramer C, Link E, Horster M, et al. Limits of the hygiene-hypothesis: Elder siblings enhance the effect of filaggrin mutations on childhood eczema - Results from the LISAplus and GINIplus study. Allergy: European Journal of Allergy and Clinical Immunology. 2010;65:47-48. 29th Congress of the European Academy of Allergy and Clinical Immunology, EAACI. London United Kingdom. 38. Cramer C, Link E, Koletzko S, et al. The hygiene hypothesis does not apply to atopic eczema in childhood. Chem Immunol Allergy. 2012;96:15-23. 39. Cvejoska-Colakovska V, Vlashki E, Kimovska M, Seckova L, Micevska V, Lawson J. Associations between indicators of socioeconomic status and childhood asthma. European Respiratory Journal. 2018;52European Respiratory Society International Congress, ERS 2018. France. 40. Delmas MC, Guignon N, Leynaert B, et al. Prevalence and control of asthma in young children in France. Rev Mal Respir. 2012;29(5):688-696. 41. Draaisma E, Garcia-Marcos L, Mallol J, Sole D, Brand P. An international comparison of eczema prevalence in infants, and the association with wheezing. European Respiratory Journal. 2014;44European Respiratory Society Annual Congress 2014. Munich Germany. 42. Duse M, Forastiere G, Porta D, Melengu T, Di Coste A, Indinnimeo L. Allergic diseases and correlated risk factors in preschool children. Annals of Allergy, Asthma and Immunology. 2014;113(5):A102-A103. 2014 Annual Meeting of the American College of Allergy, Asthma and Immunology. Atlanta, GA United States. 43. Fagbule D, Ekanem EE. Some environmental risk factors for childhood asthma: A case-control study. Annals of Tropical Paediatrics. 1994;14(1):15-19. 44. Froehlinger VJ. A STUDY OF SELECTED VARIABLES FOR THE POSITIVE IDENTIFICATION OF HANDICAPPED UNDER FOUR YEARS OF AGE (SCREENING, HIGH-RISK, AT-RISK). The Johns Hopkins University; 1984. https://search.proquest.com/docview/303301265 45. Garcia-Marcos L. Genes, environment and asthma. Anales de Pediatria Monografias. 2004;2(1):9-29. Genes, medio ambiente y asma. 46. Garcia-Marcos L, Mallol J, Sole D, Brand P, Sanchez-Solis M, Perez-Fernandez V. Factors associated to an earlier wheezing episode during the first year of life in Europe and Latin America: The EISL study. European Respiratory Journal. 2011;38European Respiratory Society Annual Congress 2011. Amsterdam Netherlands. 47. Genuneit J, Strachan D, Buchele G, et al. The effects of number of siblings, day-care attendance, and farming on childhood asthma and allergies-The GABRIEL advanced surveys. European Respiratory Journal. 2011;38European Respiratory Society Annual Congress 2011. Amsterdam Netherlands. 48. Goldstein S. The hygiene hypothesis. Asthma Magazine. 2002;7(5):20-22. 49. Gómez-Orozco L, Ordóñez de la Mora BR, Galindo Alcázar E, Martínez Ojeda A. Atopic dermatitis. Alergia. 1966;14(2):52-69. 50. Goyal JP. Prevalence and risk factors for bronchial asthma in school-going children of semi-urban area of india. Pediatric Pulmonology. 2016;51:S64. 15th InternationalCongress of Pediatric Pulmonology, CIPP 2016. Italy. 51. Guida F, Clarisse B, Momas I. Risk factors of wheezing onset during infancy in the Paris birth cohort: Benefits of the cox model. Allergy: European Journal of Allergy and Clinical Immunology. 2009;64:202. 28th Congress of the European Academy of Allergy and Clinical Immunology Abstract Book. Warszawa Poland. 52. Hedman L, Andersson M, Bjerg A, Sundberg S, Ronmark E. Incidence of asthma and wheeze among teenagers is associated with environmental risk factors. European Respiratory Journal. 2012;40European Respiratory Society Annual Congress 2012. Vienna Austria. 53. Hovland V, Riiser A, Mowinckel P, Carlsen KH, Lodrup Carlsen KC. Asthma phenotype models in childhood: Risk factors and explanatory capacity. American Journal of Respiratory and Critical Care Medicine. 2012;185American Thoracic Society International Conference, ATS 2012. San Francisco, CA United States. 54. Huet GJ, Zaat JC, Drion EF. Asthma & place among siblings. Maandschr Kindergeneeskd. 1957;25(8):265-271. 55. Hyeon-Jong Y, Yong-Hee H, You-Hoon J, Bok-Yang P. Risk Factors for Persistent Wheezing in Infants with Recurrent Wheezing. Pediatric Allergy and Respiratory Disease. 2007:17-26. 56. Johannisson A, Ponten A, Svensson A. Hand eczema in early working life: A longitudinal cohort study. Contact Dermatitis. 2012;66:32. 11th Congress of the European Society of Contact Dermatitis, ESCD 2012. Malmo Sweden. 57. K R, I R, W K. Psychological study of bronchial asthma in male children. New Egypt J Med1990. p. 1497-1502. 58. Kim KH, Kwon IH, Kim SW, et al. Epidemiologic study on the prevalence, disease severity and risk factors of atopic dermatitis in 19-year-old Korean males. Allergo Journal. 2010;19(5):320. New Trends in Allergy VII and 6th Georg Rajka Symposium on Atopic Dermatitis. Munich Germany. 59. Kliś K, Żurawiecka M, Suder A, et al. Influence of Socioeconomic Factors on Self-Reported Prevalence of Allergic Diseases Among Female University Students. Adv Exp Med Biol. 2017;1020:17-24. 60. Koplin J, Osborne N, Gurrin L, Tang M, Dharmage S, Allen K. Environmental risk factors for oral food challenge-confirmed egg allergy in a population-based study of an infant cohort. Allergy: European Journal of Allergy and Clinical Immunology. 2011;66:27-28. 30th Congress of the European Academy of Allergy and Clinical Immunology. Istanbul Turkey. 61. Koplin JJ. The epidemiology of egg allergy and other IgE-mediated food allergies in infants. University of Melbourne; 2010. http://hdl.handle.net/11343/35962. 62. Koplin JJ, Allen KJ, Molloy J, Ponsonby AL, Vuillermin PJ. Geographical differences in infant food allergy prevalence are partly explained by regional variation in the prevalence of risk factors across two regions of Victoria. Allergy: European Journal of Allergy and Clinical Immunology. 2014;69:382-383. 33rd Congress of the European Academy of Allergy and Clinical Immunology. Copenhagen Denmark. 63. Lebowitz MD, Cassell EJ, McCarroll J. Health and the urban environment. 13. The incidence and burden of minor illness in a healthy population: familial spread. Am Rev Respir Dis. 1972;106(6):842-848. 64. Lee H, Jeon YH. Environmental factors, health behaviors, and psychosocial aspects of allergic diseases in Korean adolescents. Allergy: European Journal of Allergy and Clinical Immunology. 2018;73:169-170. 37th Annual Congress of the European Academy of Allergy and Clinical Immunology, EAACI 2018. Germany. 65. Lewis S. A study of the aetiology of wheezing illness and allergic disease in children using data from the 1958 and 1970 british birth cohorts. The University of Nottingham (United Kingdom); 1997. https://search.proquest.com/docview/301546529. 66. M.S K. Some family aspects of rural bronchial asthma. Tanta Med J1984. p. 487-501. 67. Mahmood Dhahir AM, Hussein Jassim M. Ear, nose and throat [E.N.T] diseases in children: patterns and risk factors. Iraqi Postgrad Med J2008. p. 106-111. 68. Martin V, Virkud Y, Seay H, Keet C, Shreffler W, Yuan Q. A prospective assessment of food protein-induced allergic proctocolitis from the gmap healthy infant cohort. Journal of Pediatric Gastroenterology and Nutrition. 2018;67:S269-S270. North American Society for Pediatric Gastroenterology, Hepatology and Nutrition Annual Meeting, NASPGHAN 2018. United States. 69. Martins M, Reis R, Tomaz E, Inacio F. Environmental determinants in recurrent wheezing. Allergy: European Journal of Allergy and Clinical Immunology. 2014;69:367. 33rd Congress of the European Academy of Allergy and Clinical Immunology. Copenhagen Denmark. 70. McKeever TM. A birth cohort study of the aetiology of allergic disease in children using the west midlands general practice research database. Nottingham Trent University (United Kingdom); 2003. https://search.proquest.com/docview/1785780886. 71. Morales Lechuga L, Arellano Ocampo F, Rosales Peimbert E. Evaluation of various pre-, neo- and postnatal factors in the asthmatic child. Alergia. 1973;21(2):47-57. 72. Motika CA, Sharma S, Lester L, Ober C. Sex-specific rise in asthma prevalence in A U.S. Farming population. American Journal of Respiratory and Critical Care Medicine. 2011;183(1)American Thoracic Society International Conference, ATS 2011. Denver, CO United States. 73. Nielsen AM, Rasmussen S, Christoffersen MN. Morbidity of Danish infants during their first months of life. Incidence and risk factors. Ugeskr Laeger. 2002;164(48):5644-5648. 74. Olesen AB, Ellingsen AR, Larsen FS, Larsen PO, Veien NK, Thestrup-Pedersen K. Atopic dermatitis may be linked to whether a child is first- or second-born and/or the age of the mother. Acta Derm Venereol. 1996;76(6):457-460. 75. Onnis L, Di Gennaro A, Cespa G, Dentale RC. Per una revisione del concetto di cronicità: una ricerca sull'influenza della risposta del servizio. Medicina Psicosomatica. 1995;40(3):191-214. 76. Ou LS, Lee MS. Environmental risk factors of atopic disorders in taiwanese schoolchildren. Clinical and Experimental Allergy. 2012;42(12):1838-1839. 2012 Annual Meeting of the British Society for Allergy and Clinical Immunology. Nottingham United Kingdom. 77. Palti H, Bendel J, Ornoy A. Prevalence of disabilities in a national sample of 7-year-old Israeli children. Isr J Med Sci. 1992;28(7):435-441. 78. Papathoma E, Trigka M, Fouzas S, Dimitriou G. Mode of delivery and wheezing disorders during the first 3 years of life. Allergy: European Journal of Allergy and Clinical Immunology. 2014;69:577. 33rd Congress of the European Academy of Allergy and Clinical Immunology. Copenhagen Denmark. 79. Peçanha DL, Lacharité C, de Queiroz Pérez-Ramos AM. Une méthode systémique d'évaluation du fonctionnement familial: Études portant sur des familles d'enfants asthmatiques. Revue Québécoise de Psychologie. 2003;24(2):89-113. 80. Perzanowski M, Miller R, Gauvey-Kern K, Diaz D, Perera F, Goldstein I. Inverse association between birth order and specific IgE among 5-year-old children living in an inner-city community. Allergy: European Journal of Allergy and Clinical Immunology. 2009;64:205. 28th Congress of the European Academy of Allergy and Clinical Immunology Abstract Book. Warszawa Poland. 81. Perzanowski MS, Miller RL, Gauvey-Kern K, Diaz D, Perera FP, Goldstein IF. Inverse association between birth order, seroatopy and atopic wheeze among 7-year old children living in an inner-city community. American Journal of Respiratory and Critical Care Medicine. 2010;181(1)American Thoracic Society International Conference, ATS 2010. New Orleans, LA United States. 82. Popescu A, Yildirim O, Uslu Coskun B. Allergic rhinitis and upper airway infections in Romanian and Turkish children: A comparative retrospective study. Allergy: European Journal of Allergy and Clinical Immunology. 2012;67:433. 31st Congress of the European Academy of Allergy and Clinical Immunology. Geneva Switzerland. 83. Rancière F. Phénotypes respiratoires et allergiques chez l'enfant jusqu'à l'âge de 4 ans en relation avec son environnement de vie : étude de la cohorte de naissance PARIS. Paris 5; 2013. http://www.theses.fr/2013PA05S006 84. Reading R, Surridge H, Adamson R. Infant immunization and family size. J Public Health (Oxf). 2004;26(4):369-371. 85. Rechardt E. An investigation in the psychosomatic aspects of prurigo Besnier. Helsinki U., Oxford; 1970:143-143 Pages. 86. Reichert-Penetrat S. Atopic dermatitis and environment. Nouvelles Dermatologiques. 2002;21:49-51. Dermative atopique et environnement. 87. Ristevska T, Vlashki E, Kimovska-Hristova M, Cvejoska Cholakovska V, Micevska V, Sechkova L. Hygiene hypothesis in childhood asthma. Allergy: European Journal of Allergy and Clinical Immunology. 2017;72:424-425. 36th Annual Congress of the European Academy of Allergy and Clinical Immunology, EAACI 2017. Finland. 88. Saha GB, Sengupta P. Psychosomatic illness &amp; certain social aspects. Samiksa. 1984;38(2):70-75. 89. Sahebi L, Sadeghi Shabestary M. The prevalence of Asthma, allergic rhinitis, and eczema among middle school students in Tabriz (northwestern Iran). Turkish Journal of Medical Sciences. 2011;41(5):927-938. 90. Saianda A, Lobo S, Aguilar S, et al. Risk factors for wheezing and allergy in preschool children (PSC) after admission for acute bronchiolitis. European Respiratory Journal. 2011;38European Respiratory Society Annual Congress 2011. Amsterdam Netherlands. 91. Sbihi H, Dai D, Boutin RCT, et al. Multiscale factors and early asthma incidence in the CHILD Study: Using Elastic Net regularization to integrate neighborhood level, individual level and gut microbiota sequencing data. Allergy, Asthma and Clinical Immunology. 2019;159th AllerGen's Research Conference. Canada. 92. Schäfer T. What makes a child allergic? Analysis of risk factors for allergic sensitization in preschool children from East and West Germany. Allergy and Asthma Proceedings. 1999;20(1):23-27. 93. Sears MR, Subbarao P, Kozyrskyj AL, et al. Exploring origins of asthma and allergy: Early results from the Canadian Healthy Infant Longitudinal Development (CHILD) birth cohort study. Allergy: European Journal of Allergy and Clinical Immunology. 2014;69:585. 33rd Congress of the European Academy of Allergy and Clinical Immunology. Copenhagen Denmark. 94. Shaheen O, El-Soudani M. Family structure of the Egyptian asthmatics. Egypt J Psychiatry. 1978;1(1):79-98. 95. Simon MR, Havstad SL, Wegienka GR, Ownby DR, Johnson CC. Risk factors associated with transient wheezing in young children. Allergy Asthma Proc. 2008;29(2):161-165. 96. Spalter E, Kolski R, Mane-Garzon F. Genetic study of 50 asthmatic children. Archivos de Pediatria del Uruguay. 1979;50(3):139-155. 97. Tanjung C. Prognostic Factors for Atopic Dermatitis in Spontaneously Born Babies from Low Socioeconomic Background. World Allergy Organization Journal. 2016;9:182. XXIV World Allergy Congress 2015. South Korea. 98. Tay JS, Ngiam TE, Yip WC. Birth order of children with bronchial asthma. J Singapore Paediatr Soc. 1982;24(3):152-155. 99. Toaza PEP, Cortez PFP. RISK FACTORS ASSOCIATED WITH ALLERGIC RHINITIS IN CHILDREN FROM 3 TO 5 YEARS OLD. Revista Universidad Y Sociedad. 2019;11(4):135-140. 100. Tomita C, Tanaka Y, Ishii N, et al. Atopic dermatitis and related factors observed at infant physical examination at health centers. Nihon Koshu Eisei Zasshi. 1997;44(5):384-390. 101. Van Cauwenberge P, Vermeiren J. Epidemiology of allergic rhinitis. Allergy and Clinical Immunology International. 2002;14(2):86-87. 102. van's Gravesande KS, Karmaus W, Moseler M, Kohr J. Influence of maternal age and number of siblings on allergic sensitization in children. Monatsschrift Kinderheilkunde. 1998;146(5):471-475. 103. Venter C, Patil V, Grundy J, Glasbey G, Arshad SH, Dean T. Risk factors for sensitisation to food and aero-allergens over the first decade of life. Allergy: European Journal of Allergy and Clinical Immunology. 2013;68:145. European Academy of Allergy and Clinical Immunology and World Allergy Organization World Allergy and Asthma Congress 2013. Milan Italy. 104. Williams HC. Atopic dermatitis: new information from epidemiological studies. Br J Hosp Med. 1994;52(8):409-412. 105. Williams HC, Strachan D, Hay RH. ECZEMA AND FAMILY-SIZE. Clinical Research. 1992;40(2):A464-A464. 106. Williams HE, McNicol KN. The spectrum of asthma in children. Pediatr Clin North Am. 1975;22(1):43-52. 107. Withers NJ, Low JL, Holgate ST, Clough JB. Factors effecting symptom frequency in adolescents with wheeze. Thorax. 1996;51 108. Wittig HJ, McLaughlin ET, Leifer KL, Belloit JD. Risk factors for the development of allergic disease: analysis of 2,190 patient records. Ann Allergy. 1978;41(2):84-88. 109. Won CS, Han MY, Ahn JC. Environmental risk factors for pediatric allergic rhinitis: Seongnam atopy project 2017. Allergy: European Journal of Allergy and Clinical Immunology. 2019;74:278-279. European Academy of Allergy and Clinical Immunology Congress. Portugal. 110. Wronka I, Kliś K, Jarzebak K. Association of Allergic Rhinitis in Female University Students with Socio-economic Factors and Markers of Estrogens Levels. Adv Exp Med Biol. 2016;884:53-59. 111. Yao T, Huang Y, Chiu C, et al. The relationship between preterm birth and childhood allergic rhinitis in Taiwan. Allergy: European Journal of Allergy and Clinical Immunology. 2018;73:679. 37th Annual Congress of the European Academy of Allergy and Clinical Immunology, EAACI 2018. Germany. 112. Yodfat Y, Fidel J, Cohen C, Eliakim M. Chronic bronchitis and bronchial asthma in a rural community in Israel: relation to socioenvironmental factors. Isr J Med Sci. 1979;15(7):573-578. 113. Yoon J, Choi YJ, Yang SI, et al. Prevalence and risk factors of allergic rhinitis in preschool children. Allergy: European Journal of Allergy and Clinical Immunology. 2015;70:302-303. 34th Congress of the European Academy of Allergy and Clinical Immunology. Barcelona Spain. 114. Zavoronkova J, Santare D, Isajevs S, et al. H. pylori Infection and allergy in a high prevalence population. Helicobacter. 2016;21:164-165. 29th International Workshop on Helicobacter and Microbiota in Inflammation and Cancer. Germany. 115. 梁語殷. Early life determinants of childhood wheezing disorders : evidence from Hong Kong’s “children of 1997” birth cohort. University of Hong Kong; 2017. https://hdl.handle.net/10722/252495 116. Nakamura Y, Oki I, Tanihara S, et al. Relationship between breast milk and atopic dermatitis in children. Nihon Koshu Eisei Zasshi. 1999;46(4):298-303. |
| --- | --- |
| **Not relevant** | 1. Bradley M, Kockum I, Söderhäll C, et al. Characterization by phenotype of families with atopic dermatitis. Acta Derm Venereol. 2000;80(2):106-110. 2. Braun H. Sozialverteilung einiger Psychosomatosen im Kindes- und Jugendalter. Praxis der Kinderpsychologie und Kinderpsychiatrie. 1985;34(7):269-276. 3. Hua T, Silverberg JI. Atopic dermatitis in US adults: Epidemiology, association with marital status, and atopy. Ann Allergy Asthma Immunol. 2018;121(5):622-624. 4. Al-Hammadi, S., Al-Maskari, F., & Bernsen, R. (2009). Prevalence of food allergy among schoolchdildren in Al-Ain city, United Arab Emirates. Annals of Allergy, Asthma and Immunology, 103(5), A118. http://ovidsp.ovid.com/ovidweb.cgi?T=JS&PAGE=reference&D=emed11&NEWS=N&AN=70255144 (2009 Annual Scientific Meeting of the American College of Allergy, Asthma and Immunology, ACAAI. Miami, FL United States. (var.pagings).) 5. Arnedo-Pena, A., Romeu-Gracia, M. A., Bellido-Blasco, J. B., Meseguer-Ferrer, N., Silvestre-Silvestre, E., Conde, F., Fernández-González, S., Dubon, M. A., Ortuño-Forcada, M., Fabregat-Puerto, J., Fenollosa-Amposta, C., Segura-Navas, L., Pac-Sa, M. R., Museros-Recatala, L., Vizcaino, A., & Tosca-Segura, R. (2017). Incidence of allergic rhinitis in a cohort of young adults from 13-15 years old to 23-25 years old in Castellon (Spain). Allergol Immunopathol (Madr), 45(3), 251-257. https://www.sciencedirect.com/science/article/abs/pii/S0301054616301239?via%3Dihub 6. Benn, C. S., Melbye, M., Wohlfahrt, J., Björkstén, B., & Aaby, P. (2004). Cohort study of sibling effect, infectious diseases, and risk of atopic dermatitis during first 18 months of life. Bmj, 328(7450), 1223. https://www.ncbi.nlm.nih.gov/pmc/articles/PMC416593/pdf/bmj32801223.pdf 7. Bernsen, R. (2005). Childhood asthma and allergy: the role of vaccinations and other early life events Erasmus University Medical Center]. 8. Bertuccio, P., Turati, F., Galeone, C., Naldi, L., Chatenoud, L., La Vecchia, C., Bach, J. F., Agostinis, F., Carminati, S., Neri, I., Patrizi, A., Starace, M., Berti, S., Gola, M. F., Gola, M., Martelli, A., Origgi, D., Serradori, L., Burroni, A. G., . . . Peroni, D. (2020). Markers of microbial exposure lower the incidence of atopic dermatitis. Allergy: European Journal of Allergy and Clinical Immunology, 75(1), 104-115. 9. Biagini, J. M., LeMasters, G. K., Ryan, P. H., Levin, L., Reponen, T., Bernstein, D. I., Villareal, M., Hershey, G. K. K., Burkle, J., & Lockey, J. (2006). Environmental risk factors of rhinitis in early infancy. Pediatric Allergy and Immunology, 17(4), 278-284. 10. Bunyavanich, S., Soto-Quiros, M. E., Avila, L., Laskey, D., Senter, J. M., & Celedón, J. C. (2010). Risk factors for allergic rhinitis in Costa Rican children with asthma. Allergy, 65(2), 256-263. https://www.ncbi.nlm.nih.gov/pmc/articles/PMC2807901/pdf/nihms135467.pdf 11. Butland, B. K., Strachan, D. P., Lewis, S., Bynner, J., Butler, N., & Britton, J. (1997). Investigation into the increase in hay fever and eczema at age 16 observed between the 1958 and 1970 British birth cohorts. Bmj, 315(7110), 717-721. https://www.ncbi.nlm.nih.gov/pmc/articles/PMC2127494/pdf/9314757.pdf 12. Celik, G., Sin, B., Keskin, S., Ediger, D., Bavbek, S., Mungan, D., Ozer, F., Demirel, Y. S., Gürbüz, F., & Misirligil, Z. (2002). Risk factors determining allergic airway diseases in Turkish subjects. J Asthma, 39(5), 383-390. https://www.tandfonline.com/doi/full/10.1081/jas-120004031 13. Cullinan, P., Harris, J. M., Newman Taylor, A. J., Jones, M., Taylor, P., Dave, J. R., Mills, P., Moffat, S. A., White, C. W., Figg, J. K., Moon, A. M., & Barnes, M. C. (2003). Can early infection explain the sibling effect in adult atopy? European Respiratory Journal, 22(6), 956-961. https://erj.ersjournals.com/content/erj/22/6/956.full.pdf 14. Draaisma, E., Garcia-Marcos, L., Mallol, J., Solé, D., Pérez-Fernández, V., & Brand, P. L. (2015). A multinational study to compare prevalence of atopic dermatitis in the first year of life. Pediatr Allergy Immunol, 26(4), 359-366. https://onlinelibrary.wiley.com/doi/abs/10.1111/pai.12388. 15. El Kettani, S., Lotfi, A. B., & Aichane, A. (2009). Prevalence of allergic rhinitis in a rural area of Settat, Morocco. East Mediterr Health J, 15(1), 167-177. 16. Forastiere, F., Agabiti, N., Corbo, G. M., Dell'Orco, V., Porta, D., Pistelli, R., Levenstein, S., & Perucci, C. A. (1997). Socioeconomic status, number of siblings, and respiratory infections in early life as determinants of atopy in children. Epidemiology, 8(5), 566-570. 17. Genuneit, J., Strachan, D. P., Büchele, G., Weber, J., Loss, G., Sozanska, B., Boznanski, A., Horak, E., Heederik, D., Braun-Fahrländer, C., & von Mutius, E. (2013). The combined effects of family size and farm exposure on childhood hay fever and atopy. Pediatr Allergy Immunol, 24(3), 293-298. 18. Gibbs, S., Surridge, H., Adamson, R., Cohen, B., Bentham, G., & Reading, R. (2004). Atopic dermatitis and the hygiene hypothesis: a case-control study. Int J Epidemiol, 33(1), 199-207. 19. Ha, E. K., Park, J. H., Lee, S. J., Yon, D. K., Kim, J. H., Jee, H. M., Lee, K. S., Sung, M., Kim, M. A., Shin, Y. H., & Han, M. Y. (2020). Shared and unique individual risk factors and clinical biomarkers in children with allergic rhinitis and obstructive sleep apnea syndrome. Clin Respir J, 14(3), 250-259. https://onlinelibrary.wiley.com/doi/abs/10.1111/crj.13124. 20. Haileamlak, A., Dagoye, D., Williams, H., Venn, A. J., Hubbard, R., Britton, J., & Lewis, S. A. (2005). Early life risk factors for atopic dermatitis in Ethiopian children. J Allergy Clin Immunol, 115(2), 370-376. https://www.jacionline.org/article/S0091-6749(04)02680-6/pdf. 21. Harris, J. M., Cullinan, P., Williams, H. C., Mills, P., Moffat, S., White, C., & Newman Taylor, A. J. (2001). Environmental associations with eczema in early life. Br J Dermatol, 144(4), 795-802. https://onlinelibrary.wiley.com/doi/abs/10.1046/j.1365-2133.2001.04135.x. 22. Harrop, J., Chinn, S., Verlato, G., Olivieri, M., Norbäck, D., Wjst, M., Janson, C., Zock, J. P., Leynaert, B., Gislason, D., Ponzio, M., Villani, S., Carosso, A., Svanes, C., Heinrich, J., & Jarvis, D. (2007). Eczema, atopy and allergen exposure in adults: a population-based study. Clin Exp Allergy, 37(4), 526-535. https://onlinelibrary.wiley.com/doi/abs/10.1111/j.1365-2222.2007.02679.x. 23. Hatakka, K., Piirainen, L., Pohjavuori, S., Poussa, T., Savilahti, E., & Korpela, R. (2009). Allergy in day care children: prevalence and environmental risk factors. Acta Paediatr, 98(5), 817-822. https://onlinelibrary.wiley.com/doi/10.1111/j.1651-2227.2008.01198.x. 24. Herr, M., Just, J., Nikasinovic, L., Foucault, C., Le Marec, A. M., Giordanella, J. P., & Momas, I. (2012). Risk factors and characteristics of respiratory and allergic phenotypes in early childhood. J Allergy Clin Immunol, 130(2), 389-396.e384. https://www.jacionline.org/article/S0091-6749(12)00961-X/pdf. 25. Ho, C. L., Chang, L. I., & Wu, W. F. (2019). The prevalence and risk factors of atopic dermatitis in 6-8 year-old first graders in Taipei. Pediatr Neonatol, 60(2), 166-171. https://www.pediatr-neonatol.com/article/S1875-9572(17)30616-2/pdf. 26. Hrubá, D., Kukla, L., Tyrlík, M., & Matějova, H. (2009). "The Hygiene hypothesis" and allergic diseases among children aged 3 years. Elspac study results. Hygiena, 54(4), 112-116. https://hygiena.szu.cz/pdfs/hyg/2009/04/02.pdf. 27. Hsu, S. P., Lin, K. N., Tan, C. T., Lee, F. P., & Huang, H. M. (2009). Prenatal risk factors and occurrence of allergic rhinitis among elementary school children in an urban city. Int J Pediatr Otorhinolaryngol, 73(6), 807-810. 28. Hye-Young, K. I. M., Eun-Young, J., Jae-Hoon, S. I. M., Ji-Hyun, K. I. M., Younghee, C., Su-Hwa, P., Eun-Mi, H., Youngshin, H. A. N., Kangmo, A. H. N., & Sang-Il, L. E. E. (2009). Effects of Family History on the Occurrence of Atopic Dermatitis in Infants. Pediatric Allergy and Respiratory Disease, 106-114. 29. Jisun, Y., Yean-Jung, C., Eun, L. E. E., Hyun-Ju, C. H. O., Song, I. Y., Young-Ho, K. I. M., Young-Ho, J., Ju-Hee, S. E. O., Ji-Won, K., Hyo-Bin, K. I. M., So-Yeon, L. E. E., Bong-Seong, K. I. M., Jung-Yeon, S., Eun-Jin, K. I. M., Joo-Shil, L. E. E., & Soo-Jong, H. (2017). Allergic Rhinitis in Preschool Children and the Clinical Utility of FeNO. Allergy, Asthma &amp; Immunology Research, 314-321. http://dx.doi.org/10.4168/aair.2017.9.4.314. 30. Ju-Hee, S. E. O., Hyung-Young, K. I. M., Young-Ho, J., Ji-Won, K., Byoung-Ju, K. I. M., Hyo-Bin, K. I. M., Woo-Kyung, K. I. M., So-Yeon, L. E. E., Gwang-Cheon, J., Dae-Jin, S., Jung-Yeon, S., Eun-Jin, K. I. M., Joo-Shil, L. E. E., & Soo-Jong, H. (2013). The association between sibling and allergic rhinitis in adolescents. Allergy, Asthma &amp; Respiratory Disease, 67-72. http://dx.doi.org/10.4168/aard.2013.1.1.67. 31. Karlander, R. (2013). Allergi - en påminnelse om vårt ursprung : en kvantitativ enkätundersökning över förekomsten avallergier bland studenterna på Gymnastik & idrottshögskolan i Stockholm Swedish School of Sport and Health Sciences. 32. Karmaus, W., Arshad, H., & Mattes, J. (2001). Does the sibling effect have its origin in utero? Investigating birth order, cord blood immunoglobulin E concentration, and allergic sensitization at age 4 years. Am J Epidemiol, 154(10), 909-915. 33. Kerkhof, M., Koopman, L. P., van Strien, R. T., Wijga, A., Smit, H. A., Aalberse, R. C., Neijens, H. J., Brunekreef, B., Postma, D. S., & Gerritsen, J. (2003). Risk factors for atopic dermatitis in infants at high risk of allergy: the PIAMA study. Clin Exp Allergy, 33(10), 1336-1341. https://onlinelibrary.wiley.com/doi/abs/10.1046/j.1365-2222.2003.01751.x?sid=nlm%3Apubmed 34. Kerkhof, M., Wijga, A., Smit, H. A., de Jongste, J. C., Aalberse, R. C., Brunekreef, B., Gerritsen, J., & Postma, D. S. (2005). The effect of prenatal exposure on total IgE at birth and sensitization at twelve months and four years of age: The PIAMA study. Pediatr Allergy Immunol, 16(1), 10-18. https://onlinelibrary.wiley.com/doi/10.1111/j.1399-3038.2005.00217.x. 35. Koplin, J. J., Dharmage, S. C., Ponsonby, A. L., Tang, M. L., Lowe, A. J., Gurrin, L. C., Osborne, N. J., Martin, P. E., Robinson, M. N., Wake, M., Hill, D. J., & Allen, K. J. (2012). Environmental and demographic risk factors for egg allergy in a population-based study of infants. Allergy, 67(11), 1415-1422. https://onlinelibrary.wiley.com/doi/10.1111/all.12015. 36. Kuiper, S., Muris, J. W., Dompeling, E., van Schayck, C. P., Schönberger, H. J., Wesseling, G., & Knottnerus, J. A. (2006). Association between first-degree familial predisposition of asthma and atopy (total IgE) in newborns. Clin Exp Allergy, 36(5), 594-601. https://onlinelibrary.wiley.com/doi/10.1111/j.1365-2222.2006.02467.x. 37. Kurosaka, F., Terada, T., Tanaka, A., Nakatani, Y., Yamada, K., Nishikawa, J., Oka, K., Takahashi, H., Mogami, A., Yamada, T., Nakano, T., Shima, M., & Nishio, H. (2011). Risk factors for wheezing, eczema and rhinoconjunctivitis in the previous 12 months among six-year-old children in Himeji City, Japan: food allergy, older siblings, day-care attendance and parental allergy history. Allergol Int, 60(3), 317-330. 38. Kwon, I. H., Won, C. H., Lee, D. H., Kim, S. W., Park, G. H., Seo, S. J., Park, C. W., Kim, J. W., & Kim, K. H. (2018). The Prevalence and Risk Factors of Atopic Dermatitis and Clinical Characteristics according to Disease Onset in 19-Year-Old Korean Male Subjects. Ann Dermatol, 30(1), 20-28. https://www.ncbi.nlm.nih.gov/pmc/articles/PMC5762472/pdf/ad-30-20.pdf. 39. Latifi-Pupovci, H., Lokaj-Berisha, V., & Lumezi, B. (2017). Relationship of Cord Blood Immunoglobulin E and Maternal Immunoglobulin E with Birth Order and Maternal History of Allergy in Albanian Mother/Neonate Pairs. Open Access Maced J Med Sci, 5(6), 751-756. https://www.ncbi.nlm.nih.gov/pmc/articles/PMC5661712/pdf/OAMJMS-5-751.pdf. 40. Lewis, S. A., & Britton, J. R. (1998). Measles infection, measles vaccination and the effect of birth order in the aetiology of hay fever. Clin Exp Allergy, 28(12), 1493-1500. https://doi.org/10.1046/j.1365-2222.1998.00436.x. 41. Linneberg, A., Nielsen, N. H., Madsen, F., Frølund, L., Dirksen, A., & Jørgensen, T. (2001). Factors related to allergic sensitization to aeroallergens in a cross-sectional study in adults: The Copenhagen Allergy Study. Clin Exp Allergy, 31(9), 1409-1417. https://doi.org/10.1046/j.1365-2222.2001.01178.x. 42. Linstow, M. L. V., Porsbjerg, C., Ulrik, C. S., Nepper‐Christensen, S., & Backer, V. (2002). Prevalence and predictors of atopy among young Danish adults. Clinical & Experimental Allergy, 32(4), 520-525. https://onlinelibrary.wiley.com/doi/abs/10.1046/j.0954-7894.2002.01326.x. 43. Loo, X. L. E., Shek, L. P., Goh, A., Teoh, O. H., Chan, Y. H., Soh, S. E., Saw, S. M., Kwek, K., Gluckman, P. D., Godfrey, K. M., Chong, Y. S., Lee, B. W., & Van Bever, H. (2014). Atopic dermatitis in early life: Arguments for at least two phenotypes? Results of the GUSTO study. Allergy: European Journal of Allergy and Clinical Immunology, 69, 235. 44. Lyons, S. A., Knulst, A. C., Burney, P. G. J., Fernández-Rivas, M., Ballmer-Weber, B. K., Barreales, L., Bieli, C., Clausen, M., Dubakiene, R., Fernández-Perez, C., Jedrzejczak-Czechowicz, M., Kowalski, M. L., Kummeling, I., Mustakov, T. B., van Os-Medendorp, H., Papadopoulos, N. G., Popov, T. A., Potts, J., Xepapadaki, P., . . . Le, T. M. (2020). Predictors of Food Sensitization in Children and Adults Across Europe. J Allergy Clin Immunol Pract. 45. Marshall, A. H., Owen, V., & Jones, N. S. (2002). More siblings, less hay fever: more evidence. Clin Otolaryngol Allied Sci, 27(5), 352-358. https://doi.org/10.1046/j.1365-2273.2002.00594.x. 46. Matheson, M. C., Dharmage, S. C., Abramson, M. J., Walters, E. H., Sunyer, J., de Marco, R., Leynaert, B., Heinrich, J., Jarvis, D., Norbäck, D., Raherison, C., Wjst, M., & Svanes, C. (2011). Early-life risk factors and incidence of rhinitis: results from the European Community Respiratory Health Study--an international population-based cohort study. J Allergy Clin Immunol, 128(4), 816-823.e815. https://www.jacionline.org/article/S0091-6749(11)00865-7/pdf. 47. Matheson, M. C., Walters, E. H., Simpson, J. A., Wharton, C. L., Ponsonby, A. L., Johns, D. P., Jenkins, M. A., Giles, G. G., Hopper, J. L., Abramson, M. J., & Dharmage, S. C. (2009). Relevance of the hygiene hypothesis to early vs. late onset allergic rhinitis. Clin Exp Allergy, 39(3), 370-378. https://onlinelibrary.wiley.com/doi/10.1111/j.1365-2222.2008.03175.x. 48. Matricardi, P. M., Franzinelli, F., Franco, A., Caprio, G., Murru, F., Cioffi, D., Ferrigno, L., Palermo, A., Ciccarelli, N., & Rosmini, F. (1998). Sibship size, birth order, and atopy in 11,371 Italian young men. J Allergy Clin Immunol, 101(4), 439-444. 49. Mattes, J., Karmaus, W., Moseler, M., Frischer, T., & Kuehr, J. (1998). Accumulation of atopic disorders within families: a sibling effect only in the offspring of atopic fathers. Clin Exp Allergy, 28(12), 1480-1486. https://doi.org/10.1046/j.1365-2222.1998.00420.x. 50. Metsälä, J., Lundqvist, A., Kaila, M., Gissler, M., Klaukka, T., & Virtanen, S. M. (2010). Maternal and perinatal characteristics and the risk of cow's milk allergy in infants up to 2 years of age: a case-control study nested in the Finnish population. Am J Epidemiol, 171(12), 1310-1316. 51. Musgrove, K., & Morgan, J. K. (1976). Infantile eczema: A long-term follow-up study. Br J Dermatol, 95(4), 365-372. https://doi.org/10.1111/j.1365-2133.1976.tb00837.x. 52. Nakamura, Y., Oki, I., Tanihara, S., Ojima, T., Ito, Y., Yamazaki, O., Iwama, M., Tabata, Y., Katsuyama, K., Sasai, Y., Nakagawa, M., Matsushita, A., Hosaka, K., Sato, J., Hidaka, Y., Uda, H., Nakamata, K., & Yanagawa, H. (2000). Relationship between breast milk feeding and atopic dermatitis in children. Journal of Epidemiology, 10(2), 74-78. https://www.jstage.jst.go.jp/article/jea1991/10/2/10_2_74/_article. 53. Newson, R. B., van Ree, R., Forsberg, B., Janson, C., Lötvall, J., Dahlén, S. E., Toskala, E. M., Baelum, J., Brożek, G. M., Kasper, L., Kowalski, M. L., Howarth, P. H., Fokkens, W. J., Bachert, C., Keil, T., Krämer, U., Bislimovska, J., Gjomarkaj, M., Loureiro, C., . . . Jarvis, D. (2014). Geographical variation in the prevalence of sensitization to common aeroallergens in adults: the GA(2) LEN survey. Allergy, 69(5), 643-651. https://onlinelibrary.wiley.com/doi/10.1111/all.12397. 54. Nishijima, H., Suzuki, S., Kondo, K., Yamasoba, T., & Yanagimoto, S. (2018). Environmental factors associated with allergic rhinitis symptoms in Japanese university students: A cross-sectional study. Auris Nasus Larynx, 45(5), 1006-1013. https://www.aurisnasuslarynx.com/article/S0385-8146(17)30643-0/fulltext. 55. Nystad, W., Skrondal, A., Njå, F., Hetlevik, O., Carlsen, K. H., & Magnus, P. (1998). Recurrent respiratory tract infections during the first 3 years of life and atopy at school age. Allergy, 53(12), 1189-1194. https://doi.org/10.1111/j.1398-9995.1998.tb03840.x. 56. Ogbuanu, I. U., Karmaus, W. J., Zhang, H., Sabo-Attwood, T., Ewart, S., Roberts, G., & Arshad, S. H. (2010). Birth order modifies the effect of IL13 gene polymorphisms on serum IgE at age 10 and skin prick test at ages 4, 10 and 18: a prospective birth cohort study. Allergy Asthma Clin Immunol, 6(1), 6. https://www.ncbi.nlm.nih.gov/pmc/articles/PMC2874524/pdf/1710-1492-6-6.pdf. 57. Olesen, A. B., Ellingsen, A. R., Olesen, H., Juul, S., & Thestrup-Pedersen, K. (1997). Atopic dermatitis and birth factors: historical follow up by record linkage. Bmj, 314(7086), 1003-1008. https://www.ncbi.nlm.nih.gov/pmc/articles/PMC2126413/pdf/9112844.pdf. 58. Palacios-Lopez, C. G., Orozco-Covarrubias, L., Tamayo-Sánchez, L., Duran-McKinster, C., & Ruiz-Maldonado, R. (2001). Atopic dermatitis: increased prevalence and the influence of birth, siblings and maternal factors. Acta Derm Venereol, 81(2), 145-146. 59. Perkin, M. R., Bader, T., Rudnicka, A. R., Strachan, D. P., & Owen, C. G. (2015). Inter-Relationship between Rhinitis and Conjunctivitis in Allergic Rhinoconjunctivitis and Associated Risk Factors in Rural UK Children. PLoS One, 10(11), e0143651. https://www.ncbi.nlm.nih.gov/pmc/articles/PMC4658044/pdf/pone.0143651.pdf. 60. Peters, R. L., Allen, K. J., Dharmage, S. C., Lodge, C. J., Koplin, J. J., Ponsonby, A. L., Wake, M., Lowe, A. J., Tang, M. L. K., Matheson, M. C., & Gurrin, L. C. (2015). Differential factors associated with challenge-proven food allergy phenotypes in a population cohort of infants: a latent class analysis. Clin Exp Allergy, 45(5), 953-963. https://onlinelibrary.wiley.com/doi/abs/10.1111/cea.12478. 61. Purvis, D. J., Thompson, J. M., Clark, P. M., Robinson, E., Black, P. N., Wild, C. J., & Mitchell, E. A. (2005). Risk factors for atopic dermatitis in New Zealand children at 3.5 years of age. Br J Dermatol, 152(4), 742-749. https://onlinelibrary.wiley.com/doi/abs/10.1111/j.1365-2133.2005.06540.x. 62. Raukas-Kivioja, A., Raukas, E. S., Meren, M., Loit, H. M., Rönmark, E., & Lundbäck, B. (2007). Allergic sensitization to common airborne allergens among adults in Estonia. Int Arch Allergy Immunol, 142(3), 247-254. https://www-karger-com.ezproxy.ub.gu.se/Article/Pdf/97027. 63. Rönmark, E., Warm, K., Bjerg, A., Backman, H., Hedman, L., & Lundbäck, B. (2017). High incidence and persistence of airborne allergen sensitization up to age 19 years. Allergy, 72(5), 723-730. https://onlinelibrary.wiley.com/doi/10.1111/all.13053. 64. Sacchetti, M., Regine, V., Mantelli, F., Chini, L., Moschese, V., Bonini, M., Pugliese, L., Suligoi, B., & Lambiase, A. (2019). Allergy screening in a schoolchildren-based population. Pediatr Allergy Immunol, 30(3), 289-295. https://onlinelibrary.wiley.com/doi/abs/10.1111/pai.13024. 65. Sardecka, I., Los-Rycharska, E., Ludwig, H., Gawryjolek, J., & Krogulska, A. (2018). Early risk factors for cow's milk allergy in children in the first year of life. Allergy and Asthma Proceedings, 39(6), e44-e54. https://www.ingentaconnect.com/content/ocean/aap/2018/00000039/00000006/art00001. 66. Sasaki, M., Yoshida, K., Adachi, Y., Furukawa, M., Itazawa, T., Odajima, H., Saito, H., Hide, M., & Akasawa, A. (2016). Environmental factors associated with childhood eczema: Findings from a national web-based survey. Allergol Int, 65(4), 420-424. 67. Sastra, S., Irsa, L., Loebis, M. S., & Evalina, R. (2016). Number of siblings and allergic rhinitis in children. Paediatrica Indonesiana, 56(1), 1-7. https://paediatricaindonesiana.org/index.php/paediatrica-indonesiana/article/view/71. 68. Schäfer, S., Liu, A., Campbell, D., & Nanan, R. (2020). Analysis of maternal and perinatal determinants of allergic sensitization in childhood. Allergy Asthma Clin Immunol, 16, 71. https://www.ncbi.nlm.nih.gov/pmc/articles/PMC7477859/pdf/13223_2020_Article_467.pdf. 69. Smt, A., & H, G. (2004). [Prevalence of atopic diseases [Allergic rhinitis, Urticaria, Eczema] and its correlations in primary school children, Shiraz, Iran]. In J. Gorgan Univ. Med. Sci. (Vol. 6, pp. 29-34). 70. Sozańska, B., Pearce, N., Błaszczyk, M., Boznański, A., & Cullinan, P. (2015). Changes in atopy prevalence and sibship effect in rural population at all ages. Allergy, 70(6), 661-666. https://onlinelibrary-wiley-com.ezproxy.ub.gu.se/doi/full/10.1111/all.12623 71. Stemeseder, T., Klinglmayr, E., Moser, S., Lang, R., Himly, M., Oostingh, G. J., Zumbach, J., Bathke, A. C., Hawranek, T., & Gadermaier, G. (2017). Influence of Intrinsic and Lifestyle Factors on the Development of IgE Sensitization. Int Arch Allergy Immunol, 173(2), 99-104. https://www.karger.com/Article/Pdf/475499 72. Strachan, D. P. (1989). Hay fever, hygiene, and household size. Bmj, 299(6710), 1259-1260. https://www.ncbi.nlm.nih.gov/pmc/articles/PMC1838109/pdf/bmj00259-0027.pdf 73. Strachan, D. P., Harkins, L. S., & Golding, J. (1997). Sibship size and self-reported inhalant allergy among adult women. ALSPAC Study Team. Clin Exp Allergy, 27(2), 151-155. https://doi.org/10.1111/j.1365-2222.1997.tb00686.x. 74. Strachan, D. P., Harkins, L. S., Johnston, I. D., & Anderson, H. R. (1997). Childhood antecedents of allergic sensitization in young British adults. J Allergy Clin Immunol, 99(1), 6-12. 75. Strachan, D. P., Taylor, E. M., & Carpenter, R. G. (1996). Family structure, neonatal infection, and hay fever in adolescence. Arch Dis Child, 74(5), 422-426. https://www.ncbi.nlm.nih.gov/pmc/articles/PMC1511536/pdf/archdisch00613-0062.pdf. 76. Svanes, C., Jarvis, D., Chinn, S., & Burney, P. (1999). Childhood environment and adult atopy: results from the European Community Respiratory Health Survey. J Allergy Clin Immunol, 103(3), 415-420. 77. Taylor-Robinson, D. C., Williams, H., Pearce, A., Law, C., & Hope, S. (2016). Do early-life exposures explain why more advantaged children get eczema? Findings from the U.K. Millennium Cohort Study. Br J Dermatol, 174(3), 569-578. https://www.ncbi.nlm.nih.gov/pmc/articles/PMC4949701/pdf/BJD-174-569.pdf. 78. Torfi, Y., Bitarafan, N., & Rajabi, M. (2015). Impact of socioeconomic and environmental factors on atopic eczema and allergic rhinitis: a cross sectional study. Excli j, 14, 1040-1048. https://www.ncbi.nlm.nih.gov/pmc/articles/PMC4800785/pdf/EXCLI-14-1040.pdf. 79. Turner, S. W., Palmer, L. J., Gibson, N. A., Rye, P. J., Goldblatt, J., Landau, L. I., & Le Souëf, P. N. (2005). The effect of age on the relationship between birth order and immunoglobulin E sensitization. Clin Exp Allergy, 35(5), 630-634. https://onlinelibrary.wiley.com/doi/10.1111/j.1365-2222.2005.02229.x. 80. Vasconcelos, A. C. L. F., Rosa, G. M. A., Massa, P. O., & Pinto, J. H. P. (2011). Prevalência de fatores associados a doenças alérgicas em crianças e adolescentes com relação à hipótese da higiene. Rev. bras. alergia imunopatol, 34(2), 49-54. https://pesquisa.bvsalud.org/gim/resource/en/lil-597376. 81. von Mutius, E., Martinez, F. D., Fritzsch, C., Nicolai, T., Reitmeir, P., & Thiemann, H. H. (1994). Skin test reactivity and number of siblings. Bmj, 308(6930), 692-695. https://www.ncbi.nlm.nih.gov/pmc/articles/PMC2539417/pdf/bmj00431-0028.pdf. 82. Wang, X., Liu, W., Hu, Y., Zou, Z., Shen, L., & Huang, C. (2016). Home environment, lifestyles behaviors, and rhinitis in childhood. Int J Hyg Environ Health, 219(2), 220-231. https://linkinghub.elsevier.com/retrieve/pii/S1438463915001571. 83. Xu, B., Järvelin, M. R., & Pekkanen, J. (1999). Prenatal factors and occurrence of rhinitis and eczema among offspring. Allergy, 54(8), 829-836. https://doi.org/10.1034/j.1398-9995.1999.00117.x. 84. Yamazaki, S., Shima, M., Nakadate, T., Ohara, T., Omori, T., Ono, M., Sato, T., & Nitta, H. (2015). Patterns of sensitization to inhalant allergens in Japanese lower-grade schoolchildren and related factors. International Archives of Allergy and Immunology, 167(4), 253-263. 85. Ziyab, A. H. (2019). Prevalence of food allergy among schoolchildren in Kuwait and its association with the coexistence and severity of asthma, rhinitis, and eczema: A cross-sectional study. World Allergy Organ J, 12(4), 100024. https://www.ncbi.nlm.nih.gov/pmc/articles/PMC6441753/pdf/main.pdf. 86. Ziyab, A. H., & Ali, Y. M. (2019). Rhinoconjunctivitis among Adolescents in Kuwait and Associated Risk Factors: A Cross-Sectional Study. Biomed Res Int, 2019, 3981064. https://www.ncbi.nlm.nih.gov/pmc/articles/PMC6878814/pdf/BMRI2019-3981064.pdf. 87. Zutavern, A., Hirsch, T., Leupold, W., Weiland, S., Keil, U., & von Mutius, E. (2005). Atopic dermatitis, extrinsic atopic dermatitis and the hygiene hypothesis: results from a cross-sectional study. Clin Exp Allergy, 35(10), 1301-1308. <https://onlinelibrary.wiley.com/doi/pdfdirect/10.1111/j.1365-2222.2005.02350.x>. |
| **Incorrect study design** | 1. Bernsen RM, al-Ramadi BK. Association between birth order and immunological parameters? Placenta. 2011;32(11):922. 2. Bernsen RM, van der Wouden JC. Does the sibling effect have its origin in utero? Investigating birth order, cord blood immunoglobulin E concentration, and allergic sensitization at age 4 years. American journal of epidemiology. 2002;156(9) 3. Bernsen RM, van der Wouden JC. Association between sibship size and allergic diseases in the Glasgow Alumni Study. Thorax. 2006;61(7):642. 4. Brims F, Chauhan AJ. Air quality, tobacco smoke, urban crowding and day care: modern menaces and their effects on health. Pediatr Infect Dis J. 2005;24(11):S152-156, discussion S156-157. 5. Burgner D, Carter K, Webster R, Kuijpers TW. Kawasaki disease, childhood allergy and the hygiene hypothesis. Pediatr Allergy Immunol. 2011;22(7):751. 6. Christiansen SC. Day care, siblings, and asthma--please, sneeze on my child. N Engl J Med. 2000;343(8):574-575. 7. Cole Johnson C, Ownby DR, Zoratti EM, Hensley Alford S, Williams LK, Joseph CLM. Environmental Epidemiology of Pediatric Asthma and Allergy. Epidemiol Rev. 2002;24(2):154-175. 8. Crane J, Pearce N, Shaw R, Fitzharris P. Asthma and having siblings. BMJ. 1994;309(6949):272. 9. Crane J, Wickens K. Antibiotics and asthma: A tricky tributary of the hygiene hypothesis. The Lancet Respiratory Medicine. 2014;2(8):595-597. 10. Crevel RWR. Hygiene and the immune system. Journal of Infection. 2001;43(1):65-69. 11. Cullinan P. Childhood allergies, birth order and family size. Thorax. 2006;61(1):3-5. 12. Cullinan P, Newman Taylor A. Asthma: environmental and occupational factors. Br Med Bull. 2003;68(1):227-242. 13. Des Roches A, Paradis L, Paradis J. Siblings, day-care attendance, and the risk of asthma and wheezing. The New England journal of medicine. 2000;343(26) 14. Diepgen TL. Atopic dermatitis: the role of environmental and social factors, the European experience. J Am Acad Dermatol. 2001;45(1):S44-48. 15. Dilley MA, Perry TT. Birth order effect on childhood food allergy. Pediatrics. 2012;130:S7-S8. 16. Dompeling E, Jobsis R, van Schayck O. Siblings, day-care attendance, and the risk of asthma and wheezing. The New England journal of medicine. 2000;343(26) 17. Dompeling EC, Jöbsis Q, Schayck CPv. Siblings, day-care attendance, and the risk on asthma and wheezing. 2000 18. Douhaud L, Bedel S, Saurel-Cubizolles MJ, et al. Cohort Profile: The EDEN mother-child cohort on the prenatal and early postnatal determinants of child health and development. International Journal of Epidemiology. 2016;45(2):353-363. 19. Doull IJ. Does pregnancy prevent atopy? Clin Exp Allergy. 2001;31(9):1335-1337. 20. Douwes J, Pearce N. Asthma and the westernization 'package'. International Journal of Epidemiology. 2002;31(6):1098-1102. 21. Ege M, Rompa S. The Hygiene Hypothesis of Allergy and Asthma. Encyclopedia of Immunobiology. 2016:328-335. 22. Ege MJ. Asthma and prenatal inflammation. American Journal of Respiratory and Critical Care Medicine. 2017;195(5):546-548. 23. Forbes L. Asthma and atopy: Endocrine or metabolic conditions? Thorax. 2005;60(10):793-794. 24. Franas A. The "hygiene hypothesis" of allergic diseases. International Review of Allergology and Clinical Immunology. 2005;11(2):41-45. 25. Fuchs O, Latzin P, Kuehni CE, Frey U. Cohort profile: The Bern Infant Lung Development cohort. International Journal of Epidemiology. 2012;41(2):366-376. 26. Fuleihan RL. The hygiene hypothesis and atopic disease. Curr Opin Pediatr. 2002;14(6):676-677. 27. Gillespie J, Siebers R, Crane J. The hygiene hypothesis: The good, the bad, and the evil - A low-down on dirt. New Zealand Journal of Medical Laboratory Science. 2003;57(3):111-115. 28. Gottschick C, Raupach-Rosin H, Langer S, et al. Cohort Profile: The LoewenKIDS Study - life-course perspective on infections, the microbiome and the development of the immune system in early childhood. Int J Epidemiol. 2019;48(4):1042-1043h. 29. Graham-Brown RA. Atopic dermatitis: predictions, expectations, and outcomes. J Am Acad Dermatol. 2001;45(1):S61-63. 30. Hendrich JE. Does exposure of young children to older siblings or to children at day-care facilities protect against the development of asthma later in childhood? J Fam Pract. 2000;49(11):1050. 31. Katz Y. Age-dependent immunomodulation of asthma. Isr Med Assoc J. 2002;4(11):875-877. 32. Kawada T. Prevalence of asthma and atopic dermatitis in children with special emphasis on birth order. Pediatr Allergy Immunol. 2012;23(8):795; author reply 796. 33. Keller MB, Lowenstein SR. Epidemiology of Asthma. Semin Respir Crit Care Med. 2002;23(4):317-330. 34. Koplin JJ, Wake M, Dharmage SC, et al. Cohort Profile: The HealthNuts Study: Population prevalence and environmental/genetic predictors of food allergy. International Journal of Epidemiology. 2015;44(4):1161-1171. 35. Lee QU. Predictive value of family history of allergy and cord blood IgE level for allergic diseases up to adulthood. Pediatric Allergy and Immunology. 2015;26(4):383-383. 36. MacDonald A. Is breast best? Is early solid feeding harmful? J R Soc Promot Health. 2003;123(3):169-174. 37. Martinez FD. Role of viral infections in the inception of asthma and allergies during childhood: could they be protective? Thorax. 1994;49(12):1189-1191. 38. Matricardi PM, Bouygue GR, Tripodi S. Inner-city asthma and the hygiene hypothesis. Ann Allergy Asthma Immunol. 2002;89(6):69-74. 39. Maziak W. Does the sibling effect have its origin in utero? Investigating birth order, cord blood immunoglobulin E concentration, and allergic sensitization at age 4 years. American journal of epidemiology. 2002;156(9) 40. Maziak W. The hygiene hypothesis and the evolutionary perspective of health. Preventive Medicine. 2002;35(4):415-418. 41. McClelland V, Watson E, Safar M. Several factors were not considered in study of increase in hay fever and eczema [12]. British Medical Journal. 1998;316(7132):707. 42. McCormack K, Leo H. Differences between preschoolers with asthma and allergies in urban and rural environments. Pediatrics. 2018;142:S255-S256. 43. McKeever T, Hubbard R, Lewis S, Britton J. Birth order. Asthma Prevention. 2005:221-238. 44. McNally NJ, Phillips DR, Williams HC. The problem of atopic eczema: aetiological clues from the environment and lifestyles. Soc Sci Med. 1998;46(6):729-741. 45. Morgan TM. Siblings, day-care attendance, and the risk of asthma and wheezing. The New England journal of medicine. 2000;343(26) 46. Morikawa A. Think globally, act locally. Asia Pacific Allergy. 2013;3(2):77-78. 47. Olesen AB. Role of the early environment for expression of atopic dermatitis. Journal of the American Academy of Dermatology. 2001;45(1):S37-S40. 48. Park YM, Seo SJ. Evidence for interactions between filaggrin null mutations and environmental exposures in the aetiology of atopic dermatitis is currently lacking. British Journal of Dermatology. 2020;183(3):411. 49. Pearce N, Douwes J. The global epidemiology of asthma in children. Int J Tuberc Lung Dis. 2006;10(2):125-132. 50. Pearson H. Children of the 90s: Coming of age. Nature. 2012;484(7393):155-158. 51. Perkin MR. Football position and atopy--both subject to the birth order effect? Bmj. 2003;327(7429):1473-1474. 52. Ponsonby AL, Kemp A. Asthma and early childhood infectious disease. Critical time for protective effect of large family on asthma may not be during first year of life. Bmj. 2001;323(7305):164-165. 53. Ramsey AC, Deane PMG. Early-life risk factors and allergic rhinitis: Comparing European and US data. Journal of Allergy and Clinical Immunology. 2011;128(4):824-825. 54. Rangaraj S, Doull I. Hormones not hygeine? Birth order and atopy. Clin Exp Allergy. 2003;33(3):277-278. 55. Roberts G. Sibs, low-level allergic sensitization and adolescents. Clinical and Experimental Allergy. 2019;49(3):264-265. 56. Romanka-Gocka K, Placek W. The contemporary aspects of risk factors and pathogenesis of atopy. Przeglad Dermatologiczny. 2004;91(1):73-79. Wspolczesne poglady na czynniki ryzyka i patogeneze atopii. 57. Rona RJ. Asthma and poverty. Thorax. 2000;55(3):239-244. 58. Semic-Jusufagic A, Simpson A, Custovic A. Environmental exposures, genetic predisposition and allergic diseases: One size never fits all. Allergy: European Journal of Allergy and Clinical Immunology. 2006;61(4):397. 59. Singh S, Jindal S, Goyal JP. Risk Factors for Bronchial Asthma in School Going Children. Indian Journal of Pediatrics. 2017;84(11):873-874. 60. Strachan D. Socioeconomic factors and the development of allergy. Toxicol Lett. 1996;86(2):199-203. 61. Strachan DP. Allergy and family size: a riddle worth solving. Clin Exp Allergy. 1997;27(3):235-236. 62. Strachan DP. Family size, infection and atopy: the first decade of the "hygiene hypothesis". Thorax. 2000;55:S2-10. 63. van Noord PA. Does the sibling effect have its origin in utero? Investigating birth order, cord blood immunoglobulin E concentration, and allergic sensitization at age 4 years. Am J Epidemiol. 2002;156(9):882-883; author reply 883-884. 64. von Mutius E. The influence of birth order on the expression of atopy in families: a gene-environment interaction? Clin Exp Allergy. 1998;28(12):1454-1456. 65. Watts J. Eczema and the family. Community Nurse. 1997;3(8):35-37. 66. Wickens K, Crane J, Pearce N, Beasley R. The magnitude of the effect of smaller family sizes on the increase in the prevalence of asthma and hay fever in the United Kingdom and New Zealand. J Allergy Clin Immunol. 1999;104(3):554-558. 67. Wijga AH, Kerkhof M, Gehring U, et al. Cohort profile: The prevention and incidence of asthma and mite allergy (PIAMA) birth cohort. International Journal of Epidemiology. 2014;43(2):527-535. 68. Zavos C, Vini D, Kountouras J, Zavos N, Trivara E. Hygiene hypothesis and protection against asthma in infants: Spending time in the countryside encountering natural allergens may boost maternal immunity. Medical Hypotheses. 2007;68(4):914-915. 69. Heinrich J, Richter K, Frye C, et al. European Community Respiratory Health Survey in Adults (ECRHS). Pneumologie. 2002;56(5):297-303. |
| **Incorrect exposure** | 1. Blackwell DL, Tonthat L. Summary health statistics for U.S. children: National Health Interview Survey, 1998. Vital Health Stat 10. 2002;(208):1-46. 2. Blackwell DL, Tonthat L. Summary health statistics for U.S. children: National Health Interview Survey, 1999. Vital Health Stat 10. 2003;(210):1-50. 3. Blackwell DL, Vickerie JL, Wondimu EA. Summary health statistics for U.S. children: National Health Interview Survey, 2000. Vital Health Stat 10. 2003;(213):1-48. 4. Bloom B, Cohen RA. Summary health statistics for U.S. children: National Health Interview Survey, 2006. Vital Health Stat 10. 2007;(234):1-79. 5. Bloom B, Cohen RA, Freeman G. Summary health statistics for U.S. children: National Health Interview Survey, 2007. Vital Health Stat 10. 2009;(239):1-80. 6. Bloom B, Cohen RA, Freeman G. Summary health statistics for U.S. children: National Health Interview Survey, 2008. Vital Health Stat 10. 2009;(244):1-81. 7. Bloom B, Cohen RA, Freeman G. Summary health statistics for U.S. children: National Health Interview Survey, 2009. Vital Health Stat 10. 2010;(247):1-82. 8. Bloom B, Cohen RA, Freeman G. Summary health statistics for U.S. children: National Health Interview Survey, 2010. Vital Health Stat 10. 2011;(250):1-80. 9. Bloom B, Cohen RA, Freeman G. Summary health statistics for u.s. Children: national health interview survey, 2011. Vital Health Stat 10. 2012;(254):1-88. 10. Bloom B, Cohen RA, Vickerie JL, Wondimu EA. Summary health statistics for U.S. children: National Health Interview Survey, 2001. Vital Health Stat 10. 2003;(216):1-54. 11. Bloom B, Dey AN. Summary health statistics for U.S. children: National Health Interview Survey, 2004. Vital Health Stat 10. 2006;(227):1-85. 12. Bloom B, Dey AN, Freeman G. Summary health statistics for U.S. children: National Health Interview Survey, 2005. Vital Health Stat 10. 2006;(231):1-84. 13. Bloom B, Jones LI, Freeman G. Summary health statistics for U.S. children: National Health Interview Survey, 2012. Vital Health Stat 10. 2013;(258):1-81. 14. Bloom B, Tonthat L. Summary health statistics for U.S. children: National Health Interview Survey, 1997. Vital Health Stat 10. 2002;(203):1-46. 15. Bråbäck L, Hjern A, Rasmussen F. Trends in asthma, allergic rhinitis and eczema among Swedish conscripts from farming and non-farming environments. A nationwide study over three decades. Clin Exp Allergy. 2004;34(1):38-43. 16. Clark ML, Reynolds SJ, Hendrikson E, Peel JL. Asthma prevalence and risk factor assessment of an underserved and primarily Latino child population in Colorado. J Environ Health. 2014;76(6):8-16. 17. Dey AN, Bloom B. Summary health statistics for U.S. children: National Health Interview Survey, 2003. Vital Health Stat 10. 2005;(223):1-78. 18. Dey AN, Schiller JS, Tai DA. Summary health statistics for U.S. children: National Health Interview Survey, 2002. Vital Health Stat 10. 2004;(221):1-78. 19. Gabriele C, Silva LM, Arends LR, et al. Early respiratory morbidity in a multicultural birth cohort: the Generation R Study. Eur J Epidemiol. 2012;27(6):453-462. 20. Granell R, Sterne JA, Henderson J. Associations of different phenotypes of wheezing illness in early childhood with environmental variables implicated in the aetiology of asthma. PLoS One. 2012;7(10):e48359. 21. Han DH, Ahn JC, Mun SJ, Kim JW, Lee CH, Rhee CS. Novel risk factors for allergic rhinitis in Korean elementary school children. Allergy: European Journal of Allergy and Clinical Immunology. 2014;69:341. 33rd Congress of the European Academy of Allergy and Clinical Immunology. Copenhagen Denmark. 22. Hobane L. The influence of socio-economic status on the prevalence of food sensitisation and food allergy in children 12 to 36 months in urban Cape Town, South Africa. University of Cape Town; 2016. https://hdl.handle.net/11427/22948 23. Kaplan BA, Mascie-Taylor CGN. Predicting the duration of childhood asthma: Introduction. Journal of Asthma. 1992;29(1):39-48. 24. Ledogar RJ, Penchaszadeh A, Garden CC, Iglesias G. Asthma and Latino cultures: different prevalence reported among groups sharing the same environment. Am J Public Health. 2000;90(6):929-935. 25. Levin M, Hobane L, Basera W, Botha M, Gray C, Zar H. Socio-economic status influences the prevalence of food sensitisation and food allergy in urban Cape Town children. World Allergy Organization Journal. 2017;10:25. WAO International Scientific Conference (WISC 2016). Israel. 26. Lombardi C, Canonica G, Passalacqua G. Cross-sectional comparison of the characteristics of respiratory allergy in immigrants and Italian children. Allergy: European Journal of Allergy and Clinical Immunology. 2012;67:218. 31st Congress of the European Academy of Allergy and Clinical Immunology. Geneva Switzerland. 27. Lowe AJ, Lodge CJ, Allen KJ, et al. Cohort Profile: Melbourne Atopy Cohort study (MACS). International Journal of Epidemiology. 2017;46(1):25-26. 28. Martel M-J, Rey É, Malo J-L, et al. Determinants of the Incidence of Childhood Asthma: A Two-Stage Case-Control Study. American Journal of Epidemiology. 2009;169(2):195-205. 29. McKeever TM, Lewis SA, Smith C, Hubbard R. The importance of prenatal exposures on the development of allergic disease: a birth cohort study using the West Midlands General Practice Database. Am J Respir Crit Care Med. 2002;166(6):827-832. 30. Norbäck D, Lu C, Wang J, et al. Asthma and rhinitis among Chinese children - Indoor and outdoor air pollution and indicators of socioeconomic status (SES). Environ Int. 2018;115:1-8. 31. Palvo F, Toledo EC, Menin A, Jorge PPO, Godoy MF, Sole D. Risk factors of childhood asthma in Sao Jose do Rio Preto, Sao Paulo, Brazil. Journal of Tropical Pediatrics. 2008;54(4):253-257. 32. Parasuraman SR, Ghandour RM, Kogan MD. Epidemiological Profile of Health and Behaviors in Middle Childhood. Pediatrics. 2020;145(6) 33. Pearson RSB. Asthma in Barbados. Clinical & Experimental Allergy. 1973;3(3):289-297. 34. Salehi M, Bakhshaee M, Ashtiani SJ, Najafi M, Sehatbakhsh S, Hossainzadeh M. Parental smoking and allergic rhinitis in children. Int Forum Allergy Rhinol. 2014;4(5):357-360. 35. Strangert K. Respiratory illness in preschool children with different forms of day care. Pediatrics. 1976;57(2):191-196. 36. Wang D, Xiao W, Ma D, et al. A survey of asthma in Jinan. Respirology. 2013;18(2):313-322. 37. M F, M S, A.R V. [&lt;The&gt; prevalence of saffron pollen allergy in saffron workers of Khorasan [Iran] in 2002]. J Kerman Univ Med Sci2005. p. 7-13. 38. Chellini E, Talassi F, Corbo G, et al. Environmental, social and demographic characteristics of children and adolescents, resident in different Italian areas. Epidemiol Prev. 2005;29(2):14-23. 39. Doğan Ü, Ağca S. Investigation of possible risk factors in the development of seasonal allergic conjunctivitis. Int J Ophthalmol. 2018;11(9):1508-1513. 40. Kim JS, Ouyang F, Pongracic JA, et al. Dissociation between the prevalence of atopy and allergic disease in rural China among children and adults. J Allergy Clin Immunol. 2008;122(5):929-935.e924. 41. Lebowitz MD. The relationship of socio-environmental factors to the prevalence of obstructive lung diseases and other chronic conditions. J Chronic Dis. 1977;30(9):599-611. 42. Lombardi C, Canonica GW, Passalacqua G. The possible influence of the environment on respiratory allergy: a survey on immigrants to Italy. Ann Allergy Asthma Immunol. 2011;106(5):407-411. 43. Majkowska-Wojciechowska B, Laskowska B, Wojciechowski Z, Kowalski ML. Prevalence of allergy in school children in Lodz. Relation to home and school environment. Alergia Astma Immunologia. 2000;5(2):115-122. Wystepowanie alergii wsrod dzieci lodzkich szkol podstawowych: Zwiazek z warunkami srodowiska domowego i szkolnego. 44. Mojgan S, Mohsen J. [Prevalence of allergic rhinitis in 13-14 year old school children in Hamedan]. Iran J Pediatr2008. p. 41-46. 45. Rona RJ, du VFC. National study of health and growth: respiratory symptoms and height in primary schoolchildren. Int J Epidemiol. 1980;9(1):35-43. 46. Sang-Heon CHO, Yoon-Keun KIM, Yoon-Seok C, Sun-Sin KIM, Kyung-Up MIN, You-Young KIM. Asthma insights and reality in Korea. Korean Journal of Medicine. 2006:69-77. 47. Tantoco JC, Elliott Bontrager J, Zhao Q, DeLine J, Seroogy CM. The Amish have decreased asthma and allergic diseases compared with old order Mennonites. Ann Allergy Asthma Immunol. 2018;121(2):252-253.e251. 48. Weitzman M, Gortmaker S, Sobol A. Racial, social, and environmental risks for childhood asthma. Am J Dis Child. 1990;144(11):1189-1194. 49. Peat, J. K., Woolcock, A. J., Leeder, S. R., & Blackburn, C. R. B. (1980). ASTHMA AND BRONCHITIS IN SYDNEY SCHOOLCHILDREN: I. PREVALENCE DURING A SIX-YEAR STUDY. American Journal of Epidemiology, 111(6), 721-727. |
| **Conference abstract** | 1. Besednjak-Kocijancic L. Assessment of risk factors for development of allergic diseases inSlovenian twins. Allergy: European Journal of Allergy and Clinical Immunology. 2015;70:297-298. 34th Congress of the European Academy of Allergy and Clinical Immunology. Barcelona Spain. 2. Chung E, Park KS, Choi YJ, Park J, Hong SJ, Lee SY. The Risk Factors and Lung Function of Current Allergic Rhinitis Due to Dust Mite Sensitization. World Allergy Organization Journal. 2016;9:151-152. XXIV World Allergy Congress 2015. South Korea. 3. DaSilva DFG, Evans MD, Bufford JD, et al. The influence of daycare, older siblings and wheezing respiratory illnesses during infancy on the development of allergic sensitization. Journal of Allergy and Clinical Immunology. 2006;117(2):S318-S318. 4. Edelbroek N, Kamerbeek A, Tramper G. First year of life respiratory symptoms in premature born children. European Respiratory Journal. 2018;52European Respiratory Society International Congress, ERS 2018. France. 5. Irani C, Hallit S, Salameh P, Sahyoun G. Food Allergy in Lebanese schoolchildren: a pilot cross-sectional study. Journal of Allergy and Clinical Immunology. 2019;143(2):AB159. 2019 AAAAI Annual Meeting. United States. 6. Irani C, Mouzannar M. The hygiene hypothesis: Aeroallergen sensitization in allergic rhinitis patients living in rural and urban areas of Lebanon. Journal of Allergy and Clinical Immunology. 2013;131(2):AB36. 2013 Annual Meeting of the American Academy of Allergy, Asthma and Immunology, AAAAI 2013. San Antonio, TX United States. 7. Johnson CC, Havstad S, Peterson EL. Older siblings and early daycare are protective against allergen-specific IgE and atopy. Journal of Allergy and Clinical Immunology. 2002;109(1):S58-S58. 8. Jones S, Uvarov O, Salema D, Connors J, Brooks-Wilson A. Early birth order and increased risk of lymphoid cancers and allergies in lymphoid cancer families. ESMO Open. 2018;3:A252. 25th Biennial Congress of the European Association for Cancer Research. Netherlands. 9. Koplin JJ, Martin PE, Tang MLK, et al. Do factors known to alter infant microbial exposures alter the risk of food allergy and eczema in a population-based infant study? Journal of Allergy and Clinical Immunology. 2012;129(2):AB231. 2012 Annual Meeting of the American Academy of Allergy, Asthma and Immunology, AAAAI 2012. Orlando, FL United States. 10. Kusunoki T, Mukaida K, Morimoto T, et al. Birth order effect on the prevalence of childhood allergy: Comparisons among different allergic diseases. Journal of Allergy and Clinical Immunology. 2011;127(2):AB139. 2011 American Academy of Allergy, Asthma and Immunology, AAAAI Annual Meeting. San Francisco, CA United States. 11. Lau M, Dharmage S, Win A, et al. Exposure to siblings in early life modifies the association between CD14 polymorphisms and allergic sensitization in adult life. European Respiratory Journal. 2015;46European Respiratory Society Annual Congress 2015. Amsterdam Netherlands. 12. Lendvai-Emmert D, Emmert V, Fusz K, Premusz V, Boncz I, Toth GP. PIH53 EXAMINING OF PERINATAL CIRCUMSTANCES IN CHILDREN WITH COWaTMS MILK PROTEIN ALLERGY. Value in Health. 2019;22:S637. ISPOR Europe 2019. Denmark. 13. Meylan P, Gallay C, Mermoud S, Lang C, Johannsen A, Christen-Zaech S. Prospective birth cohort study on environmental factors and atopic dermatitis development. British Journal of Dermatology. 2018;179(1):e28. 10th George Rajka InternationalSymposium on Atopic Dermatitis. Netherlands. 14. Park KS, Jung HH, Kwon JW, Lee SY, Hong S. Correlation between demographic characteristics and indoor allergen sensitization among a farming community (FC), a local community (LC), and an urban region (UR) in South Korea. Journal of Allergy and Clinical Immunology. 2011;127(2):AB115. 2011 American Academy of Allergy, Asthma and Immunology, AAAAI Annual Meeting. San Francisco, CA United States. 15. Peloza DL, Evans MD, Gern JE, Lemanske RF, Jackson DJ. Asthma in siblings and risk for childhood asthma. Journal of Allergy and Clinical Immunology. 2018;141(2):AB9. 2018 American Academy of Allergy, Asthma and Immunology, AAAAI and World Allergy Organization, WAO Joint Congress. United States. 16. Rennie D, Lawson J, Karunanayake C, Dosman J, Pahwa P, Senthiselvan A. Predictors of early and late onset asthma in a rural population of children: The Saskatchewan rural health study. European Respiratory Journal. 2017;50European Respiratory Society International Congress, ERS 2017. Italy. 17. Sanjana JM, Mahesh PA, Jayaraj BS, Lokesh KS. Changing trends in the prevalence of asthma and allergic rhinitis in children in Mysore, South India. European Respiratory Journal. 2014;44European Respiratory Society Annual Congress 2014. Munich Germany. 18. Shaheen M, Teklehaimanot S. 3538 Asthma and Depression in Children: the Role of Family Structure. Journal of Clinical and Translational Science. 2019;3:119-119. 19. Shinohara M, Matsumoto K. Atopic dermatitis and Environmental Factors Associated with Skin Responses during Infancy. Journal of Allergy and Clinical Immunology. 2019;143(2):AB158. 2019 AAAAI Annual Meeting. United States. 20. Srisuwatchari W, Pacharn P, Jirapongsananuruk O, Visitsunthorn N, Vichyanond P. Epidemiology of IgE-mediated allergy to wheat among thai children, another epidemic of food allergy in Asia. Journal of Allergy and Clinical Immunology. 2017;139(2):AB144. Annual Meeting of the American Academy of Allergy, Asthma and Immunology, AAAAI 2017. United States. 21. Supriyatno B, Barnita E, Gunardi H. The prevalence and risk factors of asthma in 13-14-year-old children in East Jakarta. Paediatric Respiratory Reviews. 2013;14:S83. 12th International Congress on Pediatric Pulmonology. Spain. 22. Vlashki E, Stavric K, Kimovska M, Seckova L, Asani S, Jakupi T. Nationality and asthma: The role of familar atopy and lifestyle. European Respiratory Journal. 2015;46European Respiratory Society Annual Congress 2015. Amsterdam Netherlands. 23. Wolff PT, Arison L, Rahajamiakatra A, Raserijaona F, Niggemann B. High asthma prevalence among inner-city schoolchildren in Antananarivo (Madagascar). Journal of Allergy and Clinical Immunology. 2013;131(2):AB149. 2013 Annual Meeting of the American Academy of Allergy, Asthma and Immunology, AAAAI 2013. San Antonio, TX United States. 24. Ye M, Langan S, Abuabara K. 214 Patterns of atopic eczema disease activity from birth through mid-adulthood in two British birth cohorts. Journal of Investigative Dermatology. 2019;139(5):S37. Society for Investigative Dermatology (SID) 2019. United States. 25. Yoon J. Risk Factors of Allergic Rhinitis in Preschool Children and Clinical Utility of Feno. World Allergy Organization Journal. 2016;9:60. XXIV World Allergy Congress 2015. South Korea. 26. Brugge D, Corlin L, Newhide D, Brown E, Diaz SV. Determinates of asthma estimated from children at a museum of science. Epidemiology. 2012;23(5):S605. 24th Annual Conference of the International Society for Environmental Epidemiology, ISEE 2012. Columbia, SC United States. (var.pagings). 27. Lee S, Kwon J, Seo J, et al. Lower prevalence of allergic diseases in Korean rural children is associated with farming environment and rural lifestyle. Journal of Allergy and Clinical Immunology. 2011;127(2):AB116. 2011 American Academy of Allergy, Asthma and Immunology, AAAAI Annual Meeting. San Francisco, CA United States. (var.pagings). 28. Toppila-Salmi S, Chanoine S, Karjalainen J, Pekkanen J, Bousquet J, Siroux V. Allergic multi-morbidity is associated with an increased risk of asthma in adults: A populationbased case control study. European Respiratory Journal. 2018;52European Respiratory Society International Congress, ERS 2018. France. 29. Karmaus, W., Arshad, H., & Mattes, J. (2000). The sibling effect may have its origin in utero. an investigation into cord-blood ige concentration and allergic sensitization at age four. American Journal of Epidemiology, 151(11), S4-S4. |
| **Duplicate** | 1. Benn CS, Melbye M, Wohlfahrt J, Björkstén B, Aaby P. Cohort study of sibling effect and infectious diseases on the development of atopic dermatitis--secondary publication. Ugeskr Laeger. 2005;167(16):1754-1757. 2. Huet GJ. The relation of birth order to asthma. Ned Tijdschr Geneeskd. 1956;100(2):136-137. 3. Larenas-Linnemann DES, Romero-Tapia S, Virgen C, Garcia-Marcos L. Factors related to wheezing in toddlers in a primary health care center of a tropical city in Mexico. Allergy: European Journal of Allergy and Clinical Immunology. 2019;74:785-786. European Academy of Allergy and Clinical Immunology Congress. Portugal. 4. Lombardi C, Passalacqua G. The clinical characteristics of respiratory allergy in immigrants: An Italian cross-sectional survey. Allergy: European Journal of Allergy and Clinical Immunology. 2010;65:217. 29th Congress of the European Academy of Allergy and Clinical Immunology, EAACI. London United Kingdom. 5. Passalacqua G, Lombardi C, Canonica G. The influence of environment on respiratory allergy. A survey on immigrants to Italy. Journal of Allergy and Clinical Immunology. 2011;127(2):AB154. 2011 American Academy of Allergy, Asthma and Immunology, AAAAI Annual Meeting. San Francisco, CA United States. 6. Wolff PT, Arison L, Rahasamiakatra A, Raserisaona F, Niggemann B. Bronchial asthma in urban malagasy children: The vavanystudy. Journal of Allergy and Clinical Immunology. 2012;129(2):AB166. 2012 Annual Meeting of the American Academy of Allergy, Asthma and Immunology, AAAAI 2012. Orlando, FL United States. (var.pagings). 7. Fernandez S, Suarez Medina R, Abreu Suarez G, et al. Risk factors for wheezing in infants born in Cuba: The Havana Asthma study. American Journal of Respiratory and Critical Care Medicine. 2013;187American Thoracic Society International Conference, ATS 2013. Philadelphia, PA United States. 8. Bernsen, R. (2005). Childhood asthma and allergy: the role of vaccinations and other early life events Erasmus University Medical Center]. http://hdl.handle.net/1765/6749. 9. Jedrychowski, W., Perera, F. P., Maugeri, U., Mróz, E., Flak, E., Mrozek-Budzyn, D., Edwards, S., & Musiał, A. (2008). Length at birth and effect of prenatal and postnatal factors on early wheezing phenotypes. Kraków epidemiologic cohort study. Int J Occup Med Environ Health, 21(2), 111-119. 10. Midodzi, W. K., Rowe, B. H., Majaesic, C. M., Saunders, L. D., & Senthilselvan, A. (2010). Early life factors associated with incidence of physician-diagnosed asthma in preschool children: results from the Canadian Early Childhood Development cohort study. J Asthma, 47(1), 7-13. https://www.tandfonline.com/doi/full/10.3109/02770900903380996. 11. Baker, D., & Henderson, J. (1999). Differences between infants and adults in the social aetiology of wheeze. The ALSPAC Study Team. Avon Longitudinal Study of Pregnancy and Childhood. J Epidemiol Community Health, 53(10), 636-642. <https://jech.bmj.com/content/jech/53/10/636.full.pdf> |
| **Incorrect outcome** | 1. Kaczmarski M, Kurzatkowska B. The contribution of some environmental factors to the development of cow's milk and gluten intolerance in children. Rocz Akad Med Bialymst. 1988;33:151-165. 2. Koivikko A. Childhood asthma in Finland. A survey of 559 patients. Acta Allergol. 1974;29(1):30-72. 3. Leeder SR, Corkhill R, Irwig LM, Holland WW, Colley JR. Influence of family factors on the incidence of lower respiratory illness during the first year of life. Br J Prev Soc Med. 1976;30(4):203-212. 4. Melia RJ, Florey CD, Altman DG, Swan AV. Association between gas cooking and respiratory disease in children. Br Med J. 1977;2(6080):149-152. 5. Nasreen S. Trends in Asthma Prevalence in Canadians, Asthma Course Trajectories in Children, and the Effect of Maternal Gestational Diabetes Mellitus on the Risk of Asthma in the Offspring. University of Western Ontario; 2019. https://ir.lib.uwo.ca/etd/6685 6. Kuti BP, Omole KO, Kuti DK, et al. Exercise induced bronchospasm in Ilesa, Nigeria: A comparative study of rural and urban school children. American Journal of Respiratory and Critical Care Medicine. 2017;195American Thoracic Society International Conference, ATS 2017. United States. |
| **No data** | 1. Ikemi Y, Ago Y, Nakagawa S, Mori S, Takahashi N. Psychosomatic mechanism under social changes in Japan. J Psychosom Res. 1974;18(1):15-24. |

### Table S9B. Excluded studies (update search)

| **Not relevant** | 1. Machluf Y, Farkash R, Rotkopf R, Fink D, Chaiter Y. Asthma phenotypes and associated comorbidities in a large cohort of adolescents in Israel. Journal of Asthma. 2020;57(7):722-735. 2. Madsen MK, Schlünssen V, Svanes C, et al. The Effect of Farming Environment on Asthma; Time Dependent or Universal? 2021. 3. Matsumoto N, Yorifuji T, Nakamura K, Ikeda M, Tsukahara H, Doi H. Breastfeeding and risk of food allergy: A nationwide birth cohort in Japan. Allergology International. 2020;69(1):91-97. 4. Mont D, Nguyen CV, Tran A. The effect of sibship size on children’s outcomes: Evidence from Vietnam. Child Indicators Research. 2020;13(1):147-173. 5. Mubanga M, Lundholm C, D'Onofrio BM, Stratmann M, Hedman A, Almqvist C. Association of Early Life Exposure to Antibiotics With Risk of Atopic Dermatitis in Sweden. JAMA Netw Open. 2021;4(4):e215245. 6. Pape K, Liu X, Sejbæk CS, et al. Maternal life and work stressors during pregnancy and asthma in offspring. Int J Epidemiol. 2021;49(6):1847-1855. 7. Paquin M, Paradis L, Graham F, Begin P, Des Roches A. Peanut consumption habits and incidence of new peanut allergy in a cohort of younger siblings of peanut-allergic children. J Allergy Clin Immunol Pract. 2021;9(1):539-541.e1. 8. Rueter K, Jones AP, Siafarikas A, Lim E-M, Prescott SL, Palmer DJ. In “high-risk” infants with sufficient vitamin d status at birth, infant vitamin D supplementation had no effect on allergy outcomes: A randomized controlled trial. Nutrients. 2020;12(6):1747. 9. Ta LDH, Chan JCY, Yap GC, et al. A compromised developmental trajectory of the infant gut microbiome and metabolome in atopic eczema. Gut microbes. 2020;12(1):1801964. 10. Umanets TR, Buratynska AA, Tolkach SI, et al. Impact of risk factors in developing bronchial asthma combined with gastroesophageal reflux disease in children. Medychni Perspektyvy. 2020;25(4):121. 11. Bingol, A., Uygun, D. F. K., Akdemir, M., Erengin, H., Buyuktiryaki, B., Sackesen, C., Bingol, G., Orhan, F., Sekerel, B. E., Arikoglu, T., & Altintas, D. U. (2021). Clinical phenotypes of childhood food allergies based on immune mechanisms: A multicenter study. Allergy Asthma Proc, 42(3), e86-e95. 12. Deng, L., Liu, H., Wei, D., Lu, J., Wang, C., Shen, S., He, J., & Qiu, X. (2021). Incidence of Eczema in Early Infancy and the Prenatal Risk Factors - Guangzhou, Guangdong, China, 2018-2019. China CDC Wkly, 3(33), 693-696. 13. Ho, C. L., & Wu, W. F. (2021). Risk factor analysis of allergic rhinitis in 6-8 year-old children in Taipei. PLoS One, 16(4), e0249572. 14. Loo, E. X. L., Liew, T. M., Yap, G. C., Wong, L. S. Y., Shek, L. P., Goh, A., Van Bever, H. P. S., Teoh, O. H., Yap, F., Tan, K. H., Thomas, B., Ramamurthy, M. B., Goh, D. Y. T., Eriksson, J. G., Chong, Y. S., Godfrey, K. M., Lee, B. W., & Tham, E. H. (2021). Trajectories of early-onset rhinitis in the Singapore GUSTO mother-offspring cohort. Clin Exp Allergy, 51(3), 419-429. 15. Lu, H. Y., Chiu, C. W., Kao, P. H., Tsai, Z. T., Gau, C. C., Lee, W. F., Wu, C. Y., Lan, Y. T., Hung, C. C., Chang, F. Y., Huang, Y. W., Huang, H. Y., Chang-Chien, J., Tsai, H. J., & Yao, T. C. (2020). Association between maternal age at delivery and allergic rhinitis in schoolchildren: A population-based study. World Allergy Organization Journal, 13(6). https://doi.org/10.1016/j.waojou.2020.100127. 16. Lukkarinen, M., Puosi, E., Kataja, E. L., Korhonen, L. S., Lukkarinen, H., Karlsson, L., & Karlsson, H. (2021). Maternal psychological distress during gestation is associated with infant food allergy. Pediatric Allergy and Immunology, 32(4), 787-792. 17. Morillo-Argudo, D. A., Andrade Tenesaca, D. S., Rodas-Espinoza, C. R., Perkin, M. R., Gebreegziabher, T. L., Zuñiga, G. A., Andrade Muñoz, D. D., Ramírez, P. L., García García, A. A., & Ochoa-Avilés, A. M. (2020). Food allergy, airborne allergies, and allergic sensitisation among adolescents living in two disparate socioeconomic regions in Ecuador: A cross-sectional study. World Allergy Organ J, 13(11), 100478. 18. Rangkakulnuwat, P., & Lao-Araya, M. (2021). The prevalence and temporal trends of food allergy among preschool children in Northern Thailand between 2010 and 2019. World Allergy Organization Journal, 14(10), 100593. 19. Sigurdardottir, S. T., Jonasson, K., Clausen, M., Lilja Bjornsdottir, K., Sigurdardottir, S. E., Roberts, G., Grimshaw, K., Papadopoulos, N. G., Xepapadaki, P., Fiandor, A., Quirce, S., Sprikkelman, A. B., Hulshof, L., Kowalski, M. L., Kurowski, M., Dubakiene, R., Rudzeviciene, O., Bellach, J., Yürek, S., . . . Keil, T. (2021). Prevalence and early-life risk factors of school-age allergic multimorbidity: The EuroPrevall-iFAAM birth cohort. Allergy, 76(9), 2855-2865. 20. Suaini, N. H. A., Loo, E. X. L., Peters, R. L., Yap, G. C., Allen, K. J., Van Bever, H., Martino, D. J., Goh, A. E. N., Dharmage, S. C., & Colega, M. T. (2021). Children of Asian ethnicity in Australia have higher risk of food allergy and early‐onset eczema than those in Singapore. Allergy. 21. Tong, X., Tong, H., Gao, L., Deng, Y., Xiang, R., Cen, R., Zhao, Y., Wang, P., Li, G., Shen, J., Xu, B., He, B., Kong, Y., Tao, Z., & Xu, Y. (2021). A Multicenter Study of Prevalence and Risk Factors for Allergic Rhinitis in Primary School Children in 5 Cities of Hubei Province, China. Int Arch Allergy Immunol, 1-11. |
| --- | --- |
| **Incorrect exposure** | 1. Bilocca D. Effects of household indoor air quality and environment on respiratory health of Maltese families. 2020. 2. Brooks D, Cardillo C. A Cross-Sectional Study of Household Size and Family Size and Their Association with Childhood Asthma and Childhood Obesity in Southern California in 2020. Trident University International; 2021. https://www.proquest.com/dissertations-theses/cross-sectional-study-household-size-family-their/docview/2487420695/se-2 3. Clarke AE, Elliott SJ, St. Pierre Y, Soller L, La Vieille S, Ben-Shoshan M. Demographic characteristics associated with food allergy in a Nationwide Canadian Study. Allergy, Asthma & Clinical Immunology. 2021;17(1):72. 4. Levin ME, Botha M, Basera W, et al. Environmental factors associated with allergy in urban and rural children from the South African Food Allergy (SAFFA) cohort. Journal of Allergy and Clinical Immunology. 2020;145(1):415-426. 5. Lu C, Norbäck D, Zhang Y, et al. Onset and remission of eczema at pre-school age in relation to prenatal and postnatal air pollution and home environment across China. Sci Total Environ. 2021;755:142467. 6. Mpairwe H, Nkurunungi G, Tumwesige P, et al. Risk factors associated with rhinitis, allergic conjunctivitis and eczema among schoolchildren in Uganda. Clinical & Experimental Allergy. 2021;51(1):108-119. 7. Paudel U, Pant KP. Beyond Smoking: Environmental Determinants of Asthma Prevalence in Western Nepal. Journal of Health and Pollution. 2020;10(25). 8. Rhedin S, Lundholm C, Osvald EC, Almqvist C. Pneumonia in Infancy and Risk for Asthma: The Role of Familial Confounding and Pneumococcal Vaccination. Chest. 2021;160(2):422-431. 9. Severcan EU, Bal CM, Gülen F, Tanaç R, Demir E. Identifying wheezing phenotypes in a pediatric Turkish cohort. J Asthma. 2021:1-7. 10. Simons E, Loewen K, Becker AB, et al. Prenatal egg consumption and infant sensitization to egg and peanut in the CHILD cohort. Allergy: European Journal of Allergy and Clinical Immunology. 2020;75:91-92. European Academy of Allergy and Clinical Immunology Digital Congress. London United Kingdom. 11. Skinner A, Falster K, Gunasekera H, et al. Asthma in urban Aboriginal children: A cross-sectional study of socio-demographic patterns and associations with pre-natal and current carer smoking. Journal of Paediatrics and Child Health. 2020;56(9):1448-1457. 12. Smew A, Lundholm C, Lichtenstein P, Savendahl L, Almqvist C. Familial co-aggregation of asthma and type 1 diabetes mellitus in children- a Swedish population-based cohort study. European Respiratory Journal. 2020;56European Respiratory Society International Congress, ERS 2020. Virtual. 13. Talaei M, Hughes DA, Mahmoud O, et al. Dietary intake of vitamin A, lung function, and incident asthma in childhood. European Respiratory Journal. 2021. 14. Turi KN, Gebretsadik T, Ding T, et al. Dose, timing, and spectrum of prenatal antibiotic exposure and risk of childhood asthma. Clinical Infectious Diseases. 2021;72(3):455-462. 15. Wee JH, Park MW, Min C, Park IS, Park B, Choi HG. The association between high hygiene scores and allergic rhinitis in Korean adolescents. Wiley Online Library; 1024-1030. 16. Yuenyongviwat A, Koosakuchai V, Treepaiboon Y, Jessadapakorn W, Sangsupawanich P. Risk factors of food sensitization in young children with atopic dermatitis. Asian Pac J Allergy Immunol. 2021;8:10. |
| **No abstract or full-text (reports not retrieved)** | 1. Araujo M, Martins-Dos-Santos G, Sangalho I, et al. Food allergy in siblings - doctor, is my younger child also going to be allergic? ALLERGY. 2020;75:552-552. 2. Brew B, Gibberd A, Marks G, et al. Late Breaking Abstract - Identifying preventable early risk factors for asthma in Indigenous children: a population cohort study in Western Australia. European Respiratory Journal. 2020;56European Respiratory Society International Congress, ERS 2020. Virtual. 3. Gaimei W, Yunxiao S. Risk factors of recurrent wheezing after bronchiolitis. International Journal of Pediatrics. 2021;(6):239-243. 4. Gowett MQ, Aggarwal R, Zhou LT, et al. REPRODUCTIVE ORIGINS OF ALLERGIES IN CHILDREN. Fertility and Sterility. 2021;116(3):e406-e407. 77th Scientific Congress of the American Society for Reproductive Medicine. Baltimore United States. 5. Mallet MC, Mozun R, Ardura-Garcia C, Kuehni CE, Latzin P, Moeller A. Prevalence and risk factors of chronic cough in Swiss schoolchildren. European Respiratory Journal. 2020;56European Respiratory Society International Congress, ERS 2020. Virtual. 6. Ronmark E, Bunne J, Andersson M, et al. The increase of allergic sensitization in school children in Northern Sweden has leveled. European Respiratory Journal. 2020;56European Respiratory Society International Congress, ERS 2020. Virtual. 7. Soriano V, Peters R, Koplin J, et al. Has the Prevalence of Peanut Allergy Changed Following Earlier Introduction of Peanut? The EarlyNuts Study. Journal of Allergy and Clinical Immunology. 2021;147(2):AB236. 2021 AAAAI Virtual Annual Meeting. Virtual, Online. 8. Terebessy E, Zhu J, Fong I, Zhang K, To T. Do Siblings Have the Same Childhood Risks of Asthma or Allergic Disease in Ontario, Canada? AMERICAN JOURNAL OF RESPIRATORY AND CRITICAL CARE MEDICINE. 2020;201 9. Urayama K, Furusawa Y, Hoshino E, et al. 1473 Early life immune modulatory exposures and allergy risk in Japanese children. International Journal of Epidemiology. 2021;50:dyab168-673. |
| **Duplicate** | 1. Chatenoud L, Bertuccio P, Turati F, et al. Markers of microbial exposure lower the incidence of atopic dermatitis. Allergy: European Journal of Allergy and Clinical Immunology. 2020;75(1):104-115. 2. Furuhata M, Otsuka Y, Kaneita Y, et al. Factors Associated with the Development of Childhood Asthma in Japan: A Nationwide Longitudinal Study. Maternal and Child Health Journal. 2020;24(7):911-922. 3. Ha EK, Park JH, Lee SJ, et al. Shared and unique individual risk factors and clinical biomarkers in children with allergic rhinitis and obstructive sleep apnea syndrome. Clinical Respiratory Journal. 2020;14(3):250-259. 4. Haby MM, Woolcock AJ, Leeder SR, Peat JK, Marks GB. Asthma in preschool children: Prevalence and risk factors. Thorax. 2001;56(8):589-595. 5. Larenas-Linnemann D, Romero-Tapia SJ, Virgen C, Mallol J, Baeza Bacab MA, García-Marcos L. Risk factors for wheezing in primary health care settings in the tropics. Annals of Allergy, Asthma and Immunology. 2020;124(2):179-184.e1. 6. Lyons SA, Knulst AC, Burney PGJ, et al. Predictors of Food Sensitization in Children and Adults Across Europe. J Allergy Clin Immunol Pract. 2020;8(9):3074-3083.e32. 7. Rhedin S, Lundholm C, Oswald EC, Almqvist C. Risk for asthma following pneumonia in infancy - a nationwide register-based cohort study with family design. European Respiratory Journal. 2020;56European Respiratory Society International Congress, ERS 2020. Virtual. 8. McLeod C. Prematurity, Socioeconomic Status, And Childhood Asthma: A Canadian Cohort Study. Electronic Thesis and Dissertation Repository. 2020/08/11/ 2020. |
| **Incorrect study design** | 1. Barni S, Liccioli G, Sarti L, Giovannini M, Novembre E, Mori F. Immunoglobulin E (IgE)-mediated food allergy in children: epidemiology, pathogenesis, diagnosis, prevention, and management. Medicina. 2020;56(3):111. 2. Genuneit J, Standl M. Epidemiology of Allergy: Natural Course and Risk Factors of Allergic Diseases. Handb Exp Pharmacol. 2021. 3. Lee KH, Song Y, Wu W, Yu K, Zhang G. The gut microbiota, environmental factors, and links to the development of food allergy. Clinical and Molecular Allergy. 2020;18(1):1-11. |
| **No data** | 1. Sivaswamy S, Sambandan S, Ramasamy P, Surianarayanan M. Prevalence and risk factors associated with wheezing among children and adolescents from Chennai, South India. Indian Journal of Science and Technology. 2020;13(12):1342-1346. 2. Zhou L, Xu Z, Li J, Hu F, Ren J. Recurrent wheezing after bronchiolitis caused by respiratory syncytial virus in children younger than 3 years: A 1-year follow-up study. European Journal of Inflammation. 2021;19. |
| **Incorrect outcome** | 1. Pyrhönen K, Kulmala P. Atopic diseases of the parents predict the offspring’s atopic sensitization and food allergy. Pediatric Allergy and Immunology. 2021. 2. Conrad LA, Rauh VA, Hoepner LA, et al. Report of prenatal maternal demoralization and material hardship and infant rhinorrhea and watery eyes. Ann Allergy Asthma Immunol. 2020;125(4):399-404.e2. |

# Figures

## Figure S1A. Forest plot (birth order on any wheezing)

**
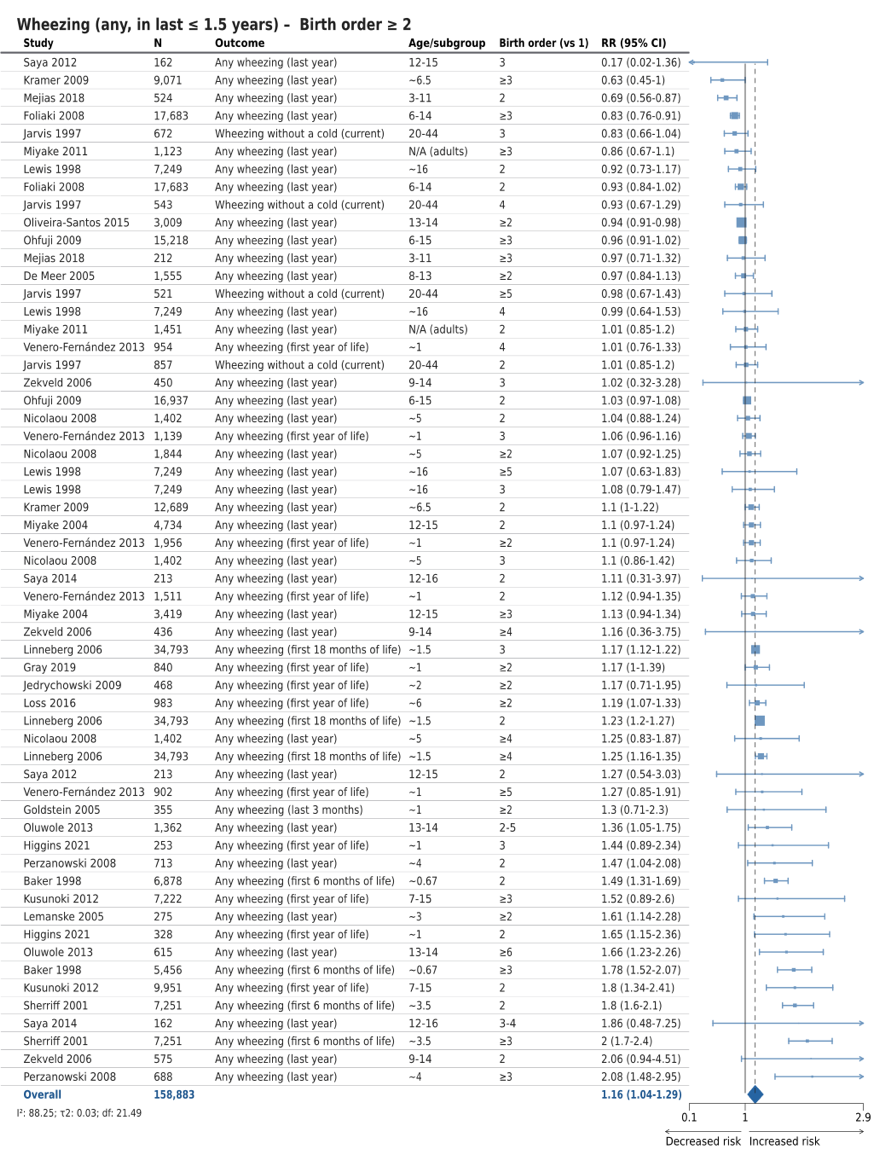
**

**Figure S1A**. Forest plot for birth order ≥ 2 vs 1 in relation to any wheezing (≥ 1 episode in last ≤ 1.5 years). **Abbrevations.** N: number of subjects (if not available, the number of subjects for the most similar exposure-outcome pair or for the whole study is stated). RR: risk ratio.

## Figure S1B. Forest plot (sibship size on any wheezing)

**
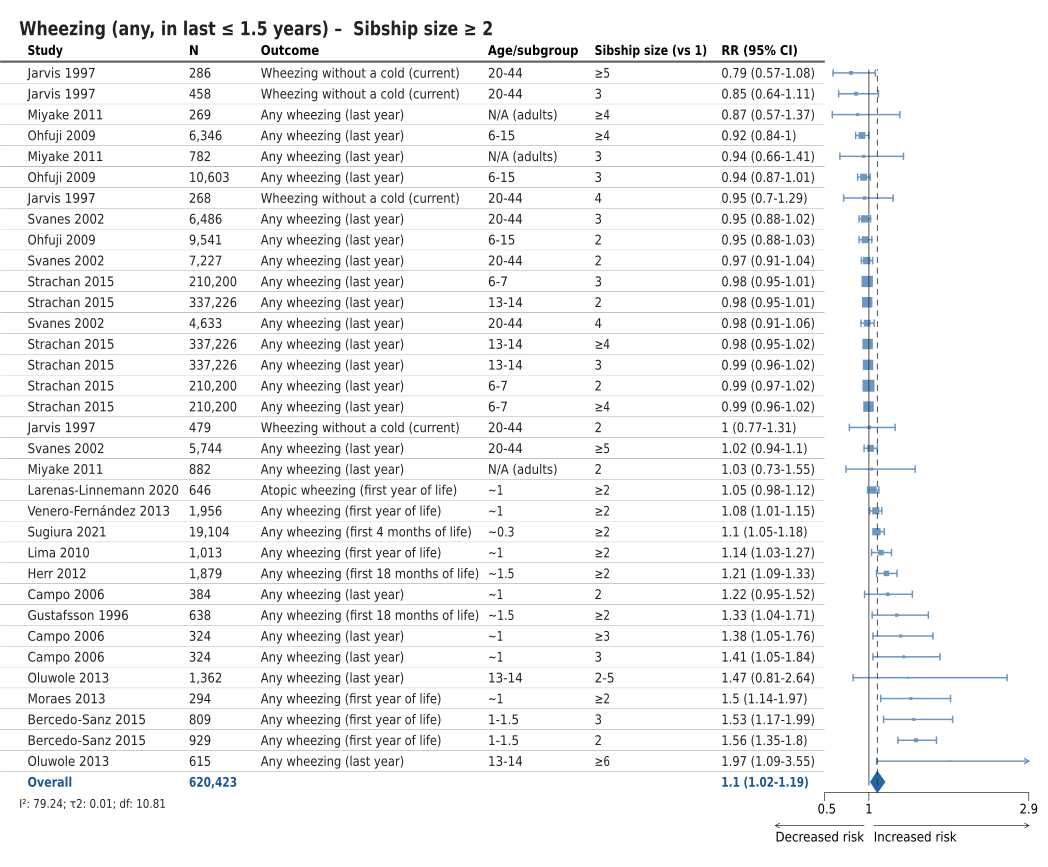
**

**Figure S1B**. Forest plot for sibship size ≥ 2 vs 1 in relation to any wheezing (≥ 1 episode in last ≤ 1.5 years). **Abbrevations.** N: number of subjects (if not available, the number of subjects for the most similar exposure-outcome pair or for the whole study is stated). RR: risk ratio.

## Figure S2A. Forest plot (birth order on recurrent wheezing)

**
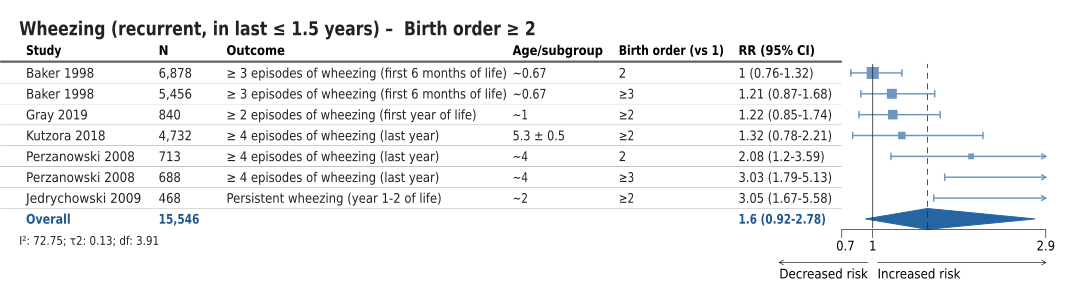
**

**Figure S2A**. Forest plot for birth order ≥ 2 vs 1 in relation to recurrent wheezing (≥ 2 episodes in last ≤ 1.5 years). **Abbrevations.** N: number of subjects (if not available, the number of subjects for the most similar exposure-outcome pair or for the whole study is stated). RR: risk ratio.

## Figure S2B. Forest plot (sibship size on recurrent wheezing)

**
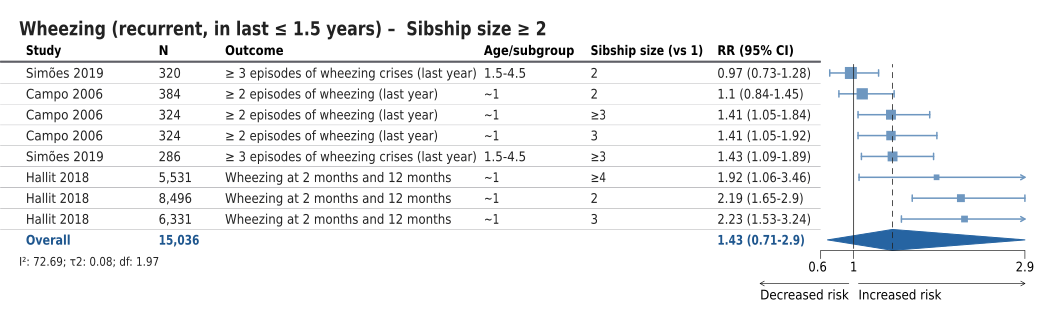
**

**Figure S2B**. Forest plot for sibship size ≥ 2 vs 1 in relation to recurrent wheezing (≥ 2 episodes in last ≤ 1.5 years). **Abbrevations.** N: number of subjects (if not available, the number of subjects for the most similar exposure-outcome pair or for the whole study is stated). RR: risk ratio.

## Figure S3A. Forest plot (birth order on current asthma)

**
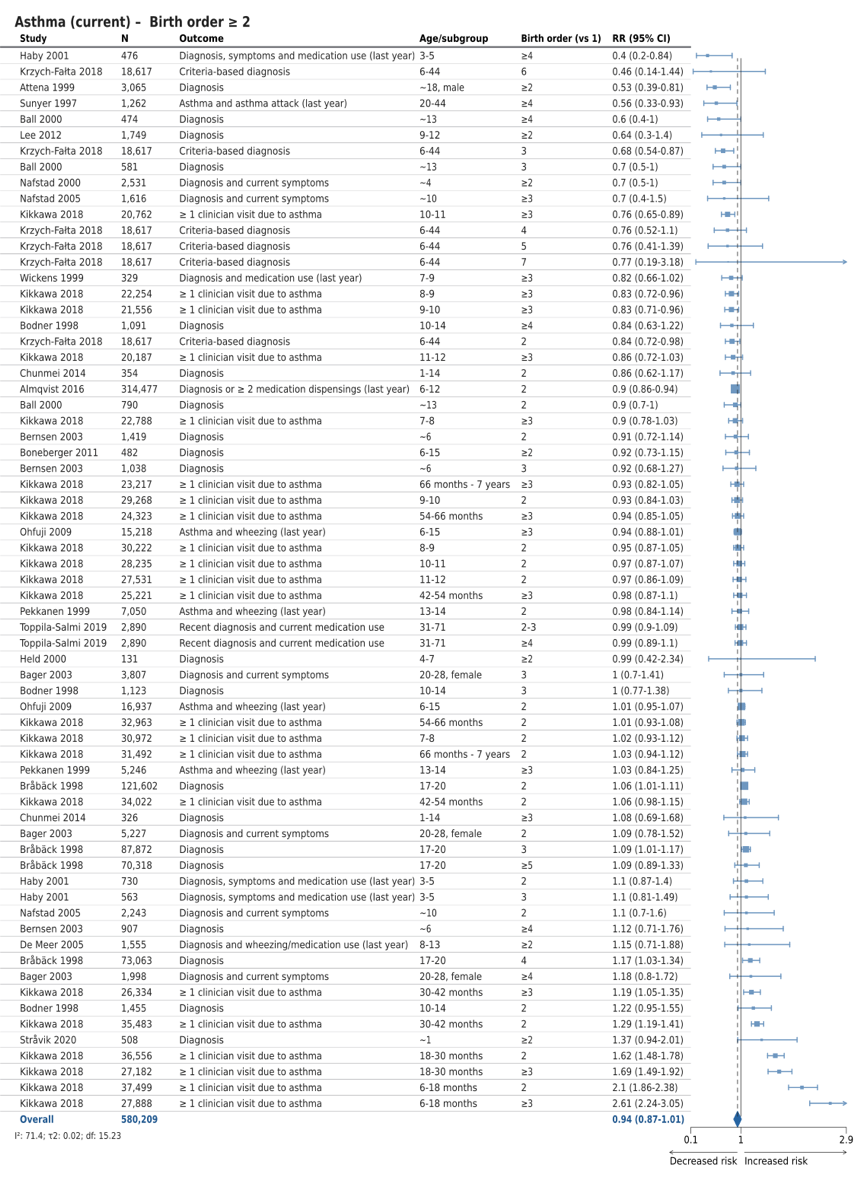
**

**Figure S3A**. Forest plot for birth order ≥ 2 vs 1 in relation to current asthma (in last year). **Abbrevations.** N: number of subjects (if not available, the number of subjects for the most similar exposure-outcome pair or for the whole study is stated). RR: risk ratio.

## Figure S3B. Forest plot (sibship size on current asthma)

**
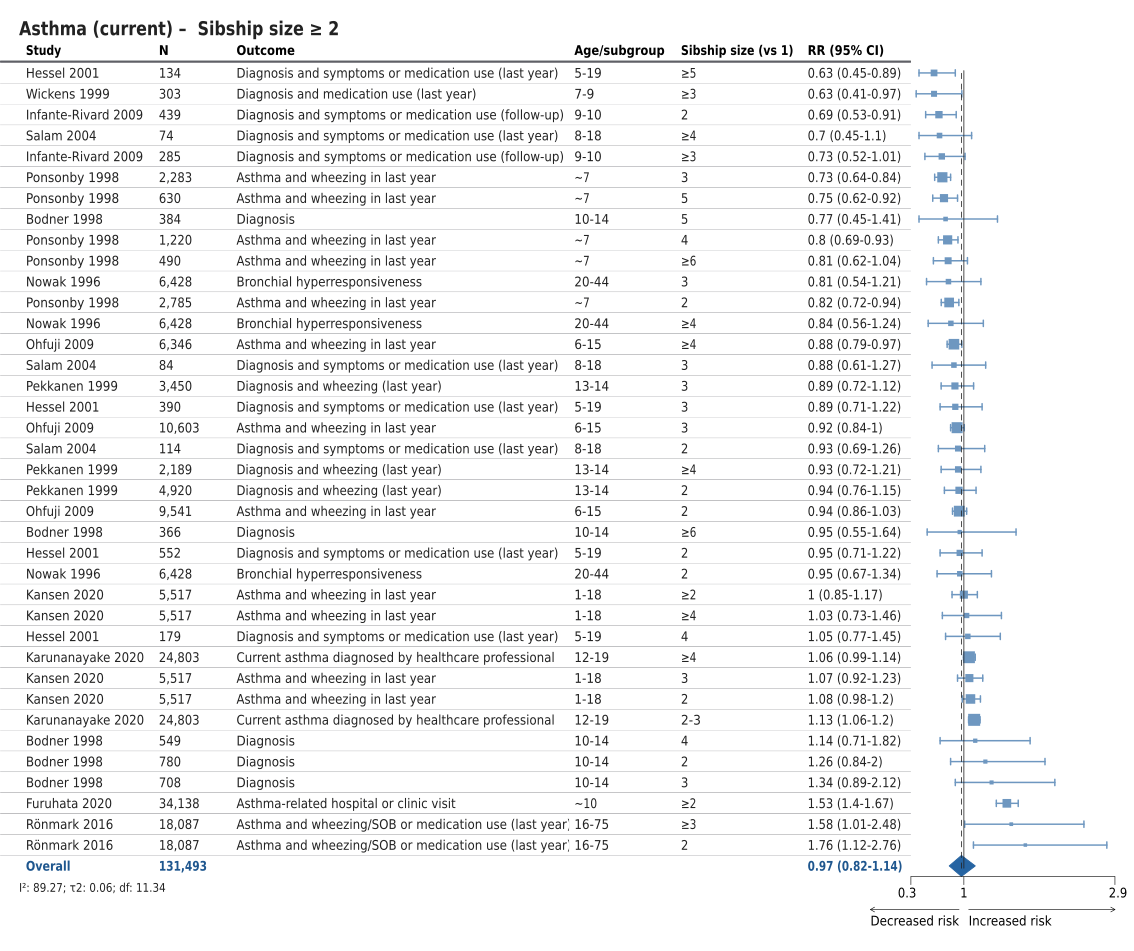
**

**Figure S3B**. Forest plot for sibship size ≥ 2 in relation to current asthma (in last year). **Abbrevations.** N: number of subjects (if not available, the number of subjects for the most similar exposure-outcome pair or for the whole study is stated). RR: risk ratio.

## Figure S4A. Forest plot (birth order on ever asthma)

**
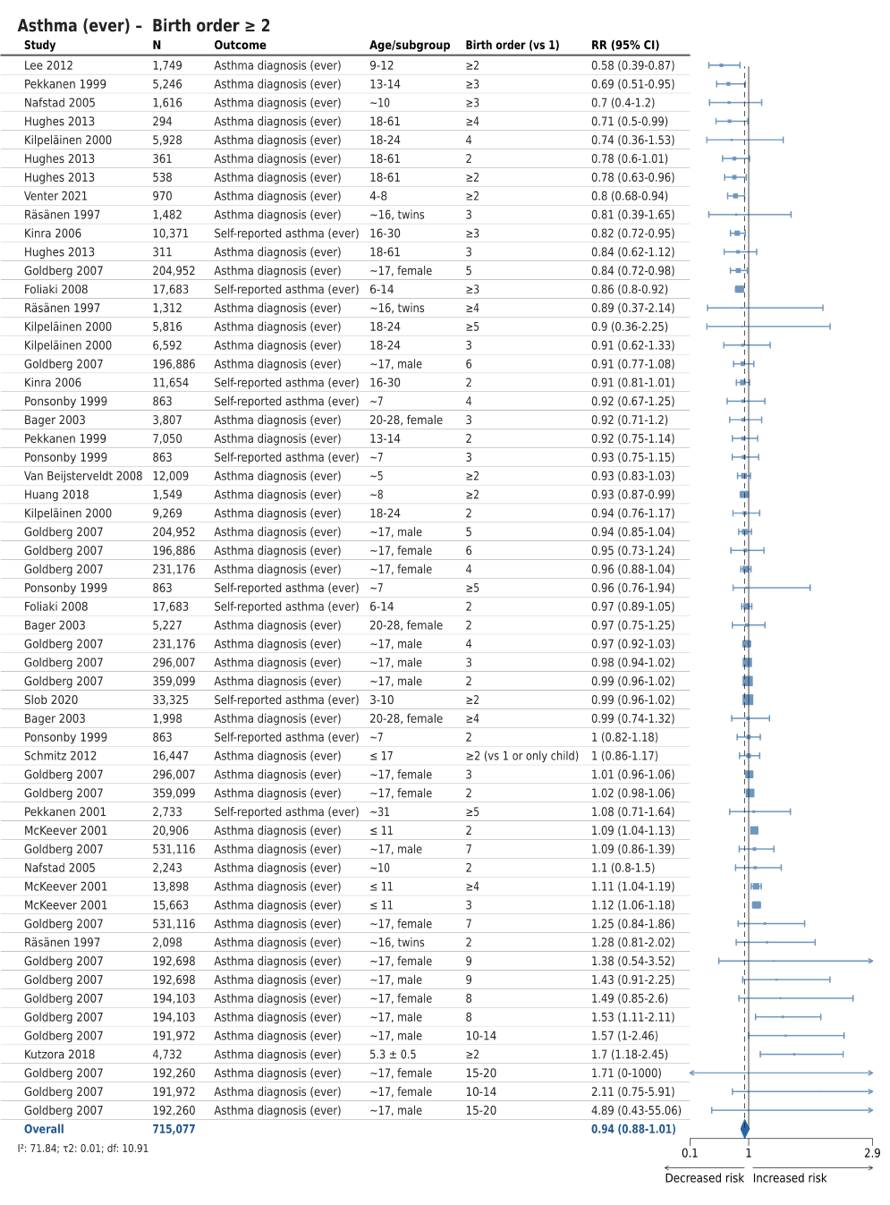
**

**Figure S4A**. Forest plot for birth order ≥ 2 vs 1 in relation to ever asthma. **Abbrevations.** N: number of subjects (if not available, the number of subjects for the most similar exposure-outcome pair or for the whole study is stated). RR: risk ratio.

## Figure S4B. Forest plot (sibship size on ever asthma)

**
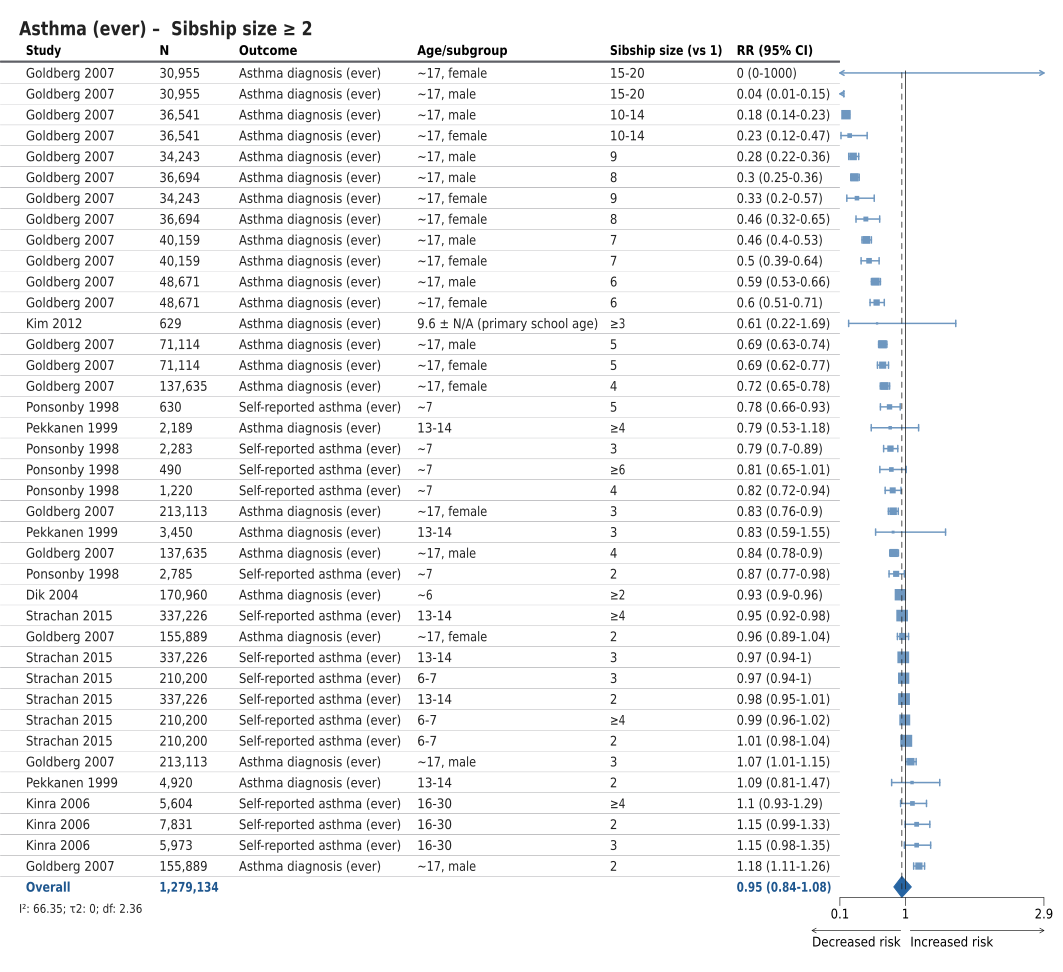
**

**Figure S4B**. Forest plot for sibship size ≥ 2 in relation to ever asthma. **Abbrevations.** N: number of subjects (if not available, the number of subjects for the most similar exposure-outcome pair or for the whole study is stated). RR: risk ratio.

## Figure S5. Funnel plots (prior to trim-and-fill)

**
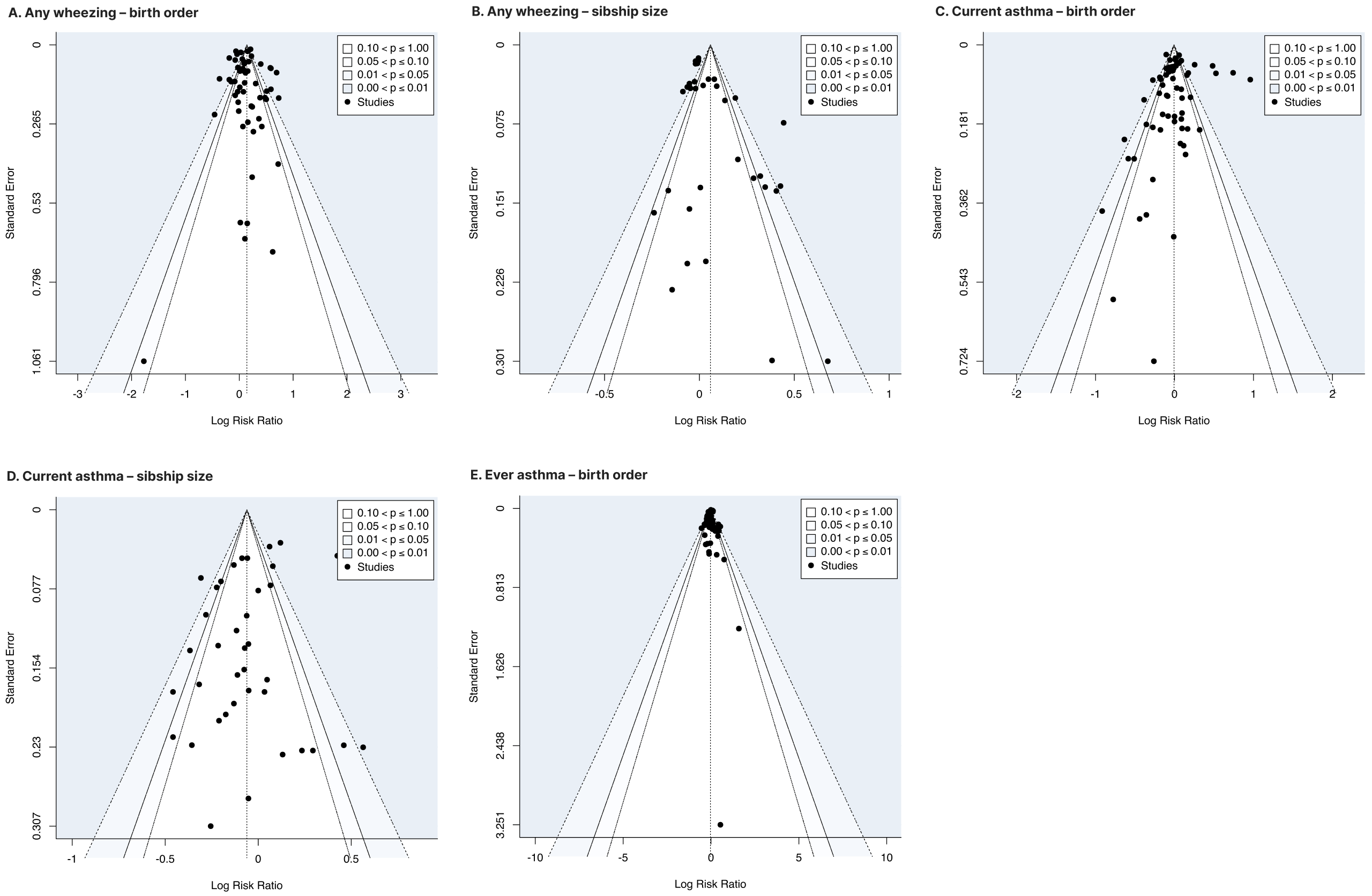
**

**Figure S5**. Funnel plots for exposure-outcome pairs with ≥ 10 studies.

## Figure S6. Funnel plots (after trim-and-fill)

**
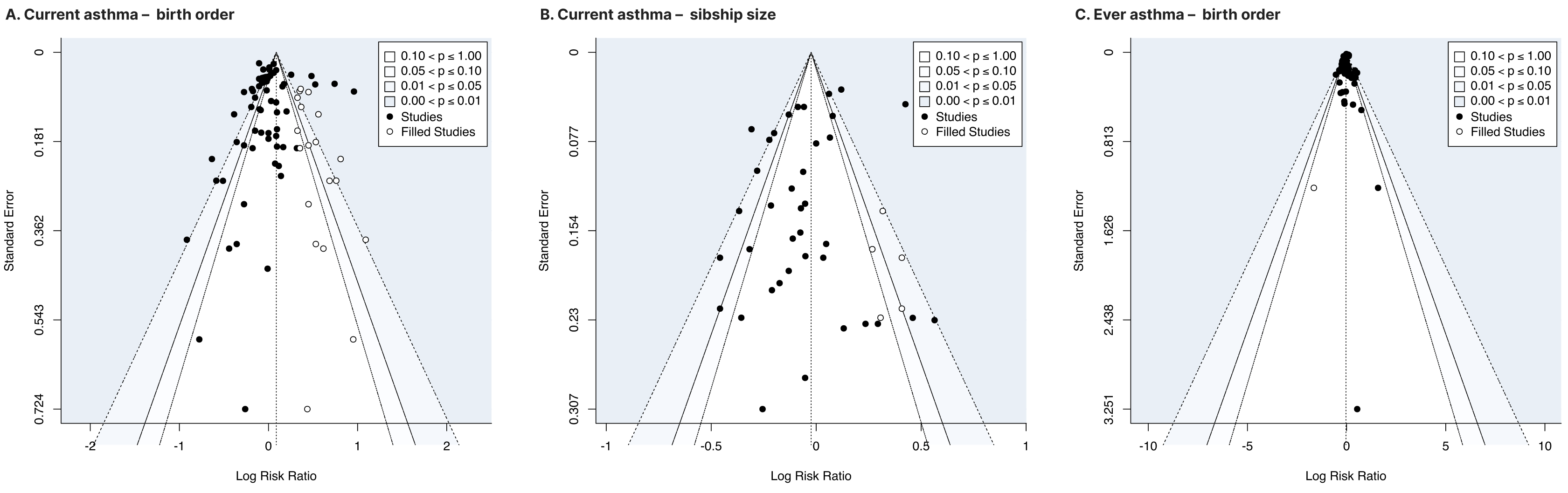
**

**Figure S6**. Funnel plots for exposure-outcome pairs with ≥ 10 studies, in which the trim-and-fill function identified asymmetry and imputed ≥ 1 estimate.

# References to all included studies

1. Aaron NS. Some personality differences between asthmatic, allergic and normal children. J Clin Psychol. 1967;23(3):336-40.

2. Addo-Yobo EO, Custovic A, Taggart SC, Craven M, Bonnie B, Woodcock A. Risk factors for asthma in urban Ghana. J Allergy Clin Immunol. 2001;108(3):363-8.

3. Adler A, Tager I, Quintero DR. Decreased prevalence of asthma among farm-reared children compared with those who are rural but not farm-reared. J Allergy Clin Immunol. 2005;115(1):67-73.

4. Akcay A, Tamay Z, Hocaoglu AB, Ergin A, Guler N. Risk factors affecting asthma prevalence in adolescents living in Istanbul, Turkey. Allergol Immunopathol (Madr). 2014;42(5):449-58.

5. Almqvist C, Olsson H, Fall T, Lundholm C. Sibship and risk of asthma in a total population: A disease comparative approach. J Allergy Clin Immunol. 2016;138(4):1219-22.e3.

6. Attena F, Agozzino E, Toscano G, Fedele N. Prevalence of asthma among young men in a military recruitment office of South Italy. Eur J Epidemiol. 1999;15(6):569-72.

7. S A, H G. [Asthma and its correlates in primary school children in Shiraz]. 2006.

8. Ayuk AC, Ramjith J, Zar HJ. Environmental risk factors for asthma in 13-14 year old African children. Pediatr Pulmonol. 2018;53(11):1475-84.

9. Bager P, Melbye M, Rostgaard K, Benn CS, Westergaard T. Mode of delivery and risk of allergic rhinitis and asthma. J Allergy Clin Immunol. 2003;111(1):51-6.

10. Baker D, Taylor H, Henderson J. Inequality in infant morbidity: causes and consequences in England in the 1990s. ALSPAC Study Team. Avon Longitudinal Study of Pregnancy and Childhood. J Epidemiol Community Health. 1998;52(7):451-8.

11. Ball TM, Castro-Rodriguez JA, Griffith KA, Holberg CJ, Martinez FD, Wright AL. Siblings, Day-Care Attendance, and the Risk of Asthma and Wheezing during Childhood. New England Journal of Medicine. 2000;343(8):538-43.

12. Batlles-Garrido J, Torres-Borrego J, Rubi-Ruiz T, Bonillo-Perales A, Gonzalez-Jimenez Y, De Cabo JM, et al. Prevalence and factors linked to atopy in 10-and 11-year-old children in Almeria, Spain. Allergologia Et Immunopathologia. 2010;38(1):13-9.

13. Bedolla-Barajas M, Javier Ramírez-Cervantes F, Morales-Romero J, Jesús Pérez-Molina J, Meza-López C, Delgado-Figueroa N. A rural environment does not protect against asthma or other allergic diseases amongst Mexican children. Allergol Immunopathol (Madr). 2018;46(1):31-8.

14. Bercedo-Sanz A, Lastra-Martínez L, Pellegrini-Belinchón J, Vicente-Galindo E, Lorente-Toledano F, García-Marcos L. Wheezing and risk factors in the first year of life in Cantabria, Spain. The EISL study. Allergol Immunopathol (Madr). 2015;43(6):543-52.

15. Bernsen RM, de Jongste JC, van der Wouden JC. Birth order and sibship size as independent risk factors for asthma, allergy, and eczema. Pediatr Allergy Immunol. 2003;14(6):464-9.

16. Bodner C, Godden D, Seaton A. Family size, childhood infections and atopic diseases. The Aberdeen WHEASE Group. Thorax. 1998;53(1):28-32.

17. Bodner C, Andersen WJ, Reid TS, Godden DJ. Childhood exposure to infection and risk of adult onset wheeze and atopy. Thorax. 2000;55(5):383-7.

18. Boneberger A, Haider D, Baer J, Kausel L, Von Kries R, Kabesch M, et al. Environmental risk factors in the first year of life and childhood asthma in the Central South of Chile. J Asthma. 2011;48(5):464-9.

19. Bråbäck L, Hedberg A. Perinatal risk factors for atopic disease in conscripts. Clin Exp Allergy. 1998;28(8):936-42.

20. Brooks K, Samms-Vaughan M, Karmaus W. Are oral contraceptive use and pregnancy complications risk factors for atopic disorders among offspring? Pediatr Allergy Immunol. 2004;15(6):487-96.

21. Burr ML, Merrett TG, Dunstan FD, Maguire MJ. The development of allergy in high-risk children. Clin Exp Allergy. 1997;27(11):1247-53.

22. Campo P, Kalra HK, Levin L, Reponen T, Olds R, Lummus ZL, et al. Influence of dog ownership and high endotoxin on wheezing and atopy during infancy. Journal of Allergy and Clinical Immunology. 2006;118(6):1271-8.

23. Chunmei JIA, Junqing W, Xiaoqin C, Dongmei W, Cairong J, Li J. A study on the relationship between pregnant, neonatal risk factors and childhood asthma. Journal of Clinical Pediatrics. 2014(12):716-9.

24. Cifuentes L, Caussade S, Villagrán C, Darrigrande P, Bedregal P, Valdivia G, et al. Risk factors for recurrent wheezing following acute bronchiolitis: a 12-month follow-up. Pediatr Pulmonol. 2003;36(4):316-21.

25. Clough JB, Keeping KA, Edwards LC, Freeman WM, Warner JA, Warner JO. Can we predict which wheezy infants will continue to wheeze? Am J Respir Crit Care Med. 1999;160(5):1473-80.

26. Cooper PJ, Vaca M, Rodriguez A, Chico ME, Santos DN, Rodrigues LC, et al. Hygiene, atopy and wheeze-eczema-rhinitis symptoms in schoolchildren from urban and rural Ecuador. Thorax. 2013;69(3):232-9.

27. Dahlen E, Wettermark B, Ekberg S, Kull I, Lundholm C, Almqvist C. Sibship and asthma medication among pre-school children. Pharmacoepidemiology and Drug Safety. 2017;26:588.

28. Davis JB, Bulpitt CJ. Atopy and wheeze in children according to parental atopy and family size. Thorax. 1981;36(3):185-9.

29. de Meer G, Janssen NA, Brunekreef B. Early childhood environment related to microbial exposure and the occurrence of atopic disease at school age. Allergy. 2005;60(5):619-25.

30. Dik N, Tate RB, Manfreda J, Anthonisen NR. Risk of physician-diagnosed asthma in the first 6 years of life. Chest. 2004;126(4):1147-53.

31. Elder DE. Recurrent wheezing in very preterm infants. Archives of Disease in Childhood: Fetal and Neonatal Edition. 1996;74(3):F165-F71.

32. Farooqi IS, Hopkin JM. Early childhood infection and atopic disorder. Thorax. 1998;53(11):927-32.

33. Foliaki S, Annesi-Maesano I, Tuuau-Potoi N, Waqatakirewa L, Cheng S, Douwes J, et al. Risk factors for symptoms of childhood asthma, allergic rhinoconjunctivitis and eczema in the Pacific: an ISAAC Phase III study. Int J Tuberc Lung Dis. 2008;12(7):799-806.

34. Freitas MS, Monteiro JC, Camelo-Nunes IC, Solé D. Prevalence of asthma symptoms and associated factors in schoolchildren from Brazilian Amazon islands. J Asthma. 2012;49(6):600-5.

35. Furuhata M, Otsuka Y, Kaneita Y, Nakagome S, Jike M, Itani O, et al. Factors Associated with the Development of Childhood Asthma in Japan: A Nationwide Longitudinal Study. Matern Child Health J. 2020;24(7):911-22.

36. Gao Z. Prevalence, incidence and risk factors of asthma and wheezing in children from Yukon, Nunavut and Northwest Territories. Ann Arbor: University of Alberta (Canada); 2005.

37. Gao H, Miao C, Li H, Bai M, Zhang H, Wu Z, et al. The effects of different parity and delivery mode on wheezing disorders in the children-a retrospective cohort study in Fujian, China. J Asthma. 2021:1-8.

38. Goldberg S, Israeli E, Schwartz S, Shochat T, Izbicki G, Toker-Maimon O, et al. Asthma prevalence, family size, and birth order. Chest. 2007;131(6):1747-52.

39. Goldstein IF, Perzanowski MS, Lendor C, Garfinkel RS, Hoepner LA, Chew GL. Prevalence of allergy symptoms and total IgE in a New York City cohort and their association with birth order. International Archives of Allergy and Immunology. 2005;137(3):249-57.

40. Grabenhenrich LB, Gough H, Reich A, Eckers N, Zepp F, Nitsche O, et al. Early-life determinants of asthma from birth to age 20 years: a German birth cohort study. J Allergy Clin Immunol. 2014;133(4):979-88.

41. Gray LEK, Ponsonby AL, Lin TX, O'Hely M, Collier F, Ranganathan S, et al. High incidence of respiratory disease in Australian infants despite low rate of maternal cigarette smoking. J Paediatr Child Health. 2019;55(12):1437-44.

42. Greenough A, Limb E, Marston L, Marlow N, Calvert S, Peacock J. Risk factors for respiratory morbidity in infancy after very premature birth. Arch Dis Child Fetal Neonatal Ed. 2005;90(4):F320-3.

43. Gupta RS, Singh AM, Walkner M, Caruso D, Bryce PJ, Wang X, et al. Hygiene factors associated with childhood food allergy and asthma. Allergy Asthma Proc. 2016;37(6):e140-e6.

44. Gustafsson D, Andersson K, Fagerlund I, Kjellman NI. Significance of indoor environment for the development of allergic symptoms in children followed up to 18 months of age. Allergy. 1996;51(11):789-95.

45. Haby MM, Peat JK, Marks GB, Woolcock AJ, Leeder SR. Asthma in preschool children: prevalence and risk factors. Thorax. 2001;56(8):589-95.

46. Hallit S, Leynaert B, Delmas MC, Rocchi S, De Blic J, Marguet C, et al. Wheezing phenotypes and risk factors in early life: The ELFE cohort. PLoS One. 2018;13(4):e0196711.

47. Hedman L, Andersson M, Bjerg A, Forsberg B, Lundbäck B, Rönmark E. Environmental risk factors related to the incidence of wheeze and asthma in adolescence. Clin Exp Allergy. 2015;45(1):184-91.

48. Held KB. A prospective cohort study of maternal factors in childhood asthma: Parity, obesity, fetal growth, and social stressors: University of Oklahoma; 2000.

49. Herr M, Just J, Nikasinovic L, Foucault C, Le Marec AM, Giordanella JP, et al. Influence of host and environmental factors on wheezing severity in infants: findings from the PARIS birth cohort. Clin Exp Allergy. 2012;42(2):275-83.

50. Hessel PA, Klaver J, Michaelchuk D, McGhan S, Carson MM, Melvin D. The epidemiology of childhood asthma in Red Deer and Medicine Hat, Alberta. Can Respir J. 2001;8(3):139-46.

51. Higgins D, Karmaus W, Jiang Y, Banerjee P, Sulaiman IM, Arshad HS. Infant wheezing and prenatal antibiotic exposure and mode of delivery: a prospective birth cohort study. Journal of Asthma. 2021;58(6):770-81.

52. Hijazi N, Abalkhail B, Seaton A. Diet and childhood asthma in a society in transition: a study in urban and rural Saudi Arabia. Thorax. 2000;55(9):775-9.

53. Hosoki K, Nagao M, Hiraguchi Y, Tokuda R, Fujisawa T. Factors related to recurrent wheezing after hospitalization with RSV infection with the children who were aged three years old or younger: a questionnaire survey. Arerugi. 2009;58(11):1513-20.

54. Hossain MM, Islam MR, Kamruzzaman M. Childhood Asthma in Bangladeshi Children: A Study in a Tertiary Care Level Hospital, Dhaka, Bangladesh.

55. Huang CC, Chiang TL, Chen PC, Lin SJ, Wen HJ, Guo YL. Risk factors for asthma occurrence in children with early-onset atopic dermatitis: An 8-year follow-up study. Pediatr Allergy Immunol. 2018;29(2):159-65.

56. Hughes AM, Lucas RM, McMichael AJ, Dwyer T, Pender MP, Mei Ivd, et al. Early-life hygiene-related factors affect risk of central nervous system demyelination and asthma differentially. Clinical & Experimental Immunology. 2013;172(3):466-74.

57. Infante-Rivard C, Amre D, Gautrin D, Malo J-L. Family Size, Day-Care Attendance, and Breastfeeding in Relation to the Incidence of Childhood Asthma. Am J Epidemiol. 2001;153(7):653-8.

58. Jarvis D, Chinn S, Luczynska C, Burney P. The association of family size with atopy and atopic disease. Clin Exp Allergy. 1997;27(3):240-5.

59. Jedrychowski W, Perera FP, Maugeri U, Mrozek-Budzyn D, Mroz E, Flak E, et al. Early wheezing phenotypes and severity of respiratory illness in very early childhood: study on intrauterine exposure to fine particle matter. Environ Int. 2009;35(6):877-84.

60. Just J, Belfar S, Wanin S, Pribil C, Grimfeld A, Duru G. Impact of innate and environmental factors on wheezing persistence during childhood. J Asthma. 2010;47(4):412-6.

61. Kansen HM, Lebbink MA, Mul J, van Erp FC, van Engelen M, de Vries E, et al. Risk factors for atopic diseases and recurrent respiratory tract infections in children. Pediatric Pulmonology. 2020;55(11):3168-79.

62. Kaplan BA, Mascie‐Taylor CGN. Varying Biosocial Correlates of Asthma and Wheezy Bronchitis. Medical Anthropology Quarterly. 1989;3(2):175-89.

63. Kaplan BA, Mascie-Taylor CG, Boldsen J. Birth order and health status in a British national sample. J Biosoc Sci. 1992;24(1):25-33.

64. Karunanayake CP, Amin K, Abonyi S, Dosman JA, Pahwa P. Prevalence and determinants of asthma among aboriginal adolescents in Canada. Journal of Asthma. 2020;57(1):40-6.

65. Kearney PM, Kearney PJ. The prevalence of asthma in schoolboys of travellers' families. Ir Med J. 1998;91(6):203-6.

66. Kiechl-Kohlendorfer U, Horak E, Mueller W, Strobl R, Haberland C, Fink F-M, et al. Neonatal characteristics and risk of atopic asthma in schoolchildren: Results from a large prospective birth-cohort study. Acta Paediatrica. 2007;96(11):1606-10.

67. Kikkawa T, Yorifuji T, Fujii Y, Yashiro M, Okada A, Ikeda M, et al. Birth order and paediatric allergic disease: A nationwide longitudinal survey. Clin Exp Allergy. 2018;48(5):577-85.

68. Kilpeläinen, Terho, Helenius, Koskenvuo. Farm environment in childhood prevents the development of allergies. Clinical and Experimental Allergy. 2000;30(2):201-8.

69. Do-Soo KIM, Mi-Ran P, Jung-Seok YU, Ho-Suk LEE, Jung-Hyun LEE, Jungmin SUH, et al. Prevalence and Risk Factors of Asthma and Allergic Rhinitis in Elementary School Children in Jinan-Gun. Pediatric Allergy and Respiratory Disease. 2012:374-82.

70. Kingsley A, Reynolds EL. The relation of illness patterns in children to ordinal position in the family. J Pediatr. 1949;35(1):17-23.

71. Kinra S, Davey Smith G, Jeffreys M, Gunnell D, Galobardes B, McCarron P. Association between sibship size and allergic diseases in the Glasgow Alumni Study. Thorax. 2006;61(1):48-53.

72. Kramer MS, Matush L, Bogdanovich N, Dahhou M, Platt RW, Mazer B. The low prevalence of allergic disease in Eastern Europe: are risk factors consistent with the hygiene hypothesis? Clin Exp Allergy. 2009;39(5):708-16.

73. Krzych-Fałta E, Furmańczyk K, Lisiecka-Biełanowicz M, Sybilski A, Tomaszewska A, Raciborski F, et al. The effect of selected risk factors, including the mode of delivery, on the development of allergic rhinitis and bronchial asthma. Postepy Dermatol Alergol. 2018;35(3):267-73.

74. Kuschnir FC, Alves Da Cunha AJL. Environmental and socio-demographic factors associated to asthma in adolescents in Rio de Janeiro, Brazil. Pediatric Allergy and Immunology. 2007;18(2):142-8.

75. Kusunoki T, Mukaida K, Morimoto T, Sakuma M, Yasumi T, Nishikomori R, et al. Birth order effect on childhood food allergy. Pediatr Allergy Immunol. 2012;23(3):250-4.

76. Kutzora S, Weber A, Heinze S, Hendrowarsito L, Nennstiel-Ratzel U, von Mutius E, et al. Asthmatic/wheezing phenotypes in preschool children: Influential factors, health care and urban-rural differences. Int J Hyg Environ Health. 2018;221(2):293-9.

77. Larenas-Linnemann D, Romero-Tapia SJ, Virgen C, Mallol J, Bacab MAB, Garcia-Marcos L. Risk factors for wheezing in primary health care settings in the tropics. Annals of Allergy Asthma & Immunology. 2020;124(2):179-+.

78. Larsson M, Hägerhed-Engman L, Sigsgaard T, Janson S, Sundell J, Bornehag CG. Incidence rates of asthma, rhinitis and eczema symptoms and influential factors in young children in Sweden. Acta Paediatr. 2008;97(9):1210-5.

79. Latzin P, Frey U, Roiha HL, Baldwin DM, Regamey N, Strippoli MPF, et al. Prospectively assessed incidence, severity, and determinants of respiratory symptoms in the first year of life. Pediatric Pulmonology. 2007;42(1):41-50.

80. Lee S-L, Wong W, Lau Y-L. Increasing prevalence of allergic rhinitis but not asthma among children in Hong Kong from 1995 to 2001 (Phase 3 International Study of Asthma and Allergies in Childhood). Pediatric Allergy and Immunology. 2004;15(1):72-8.

81. Lee SY, Kwon JW, Seo JH, Song YH, Kim BJ, Yu J, et al. Prevalence of atopy and allergic diseases in Korean children: associations with a farming environment and rural lifestyle. Int Arch Allergy Immunol. 2012;158(2):168-74.

82. Lemanske Jr RF, Jackson DJ, Gangnon RE, Evans MD, Li Z, Shult PA, et al. Rhinovirus illnesses during infancy predict subsequent childhood wheezing. Journal of Allergy and Clinical Immunology. 2005;116(3):571-7.

83. Lewis S, Richards D, Bynner J, Butler N, Britton J. Prospective study of risk factors for early and persistent wheezing in childhood. Eur Respir J. 1995;8(3):349-56.

84. Lewis S, Butland B, Strachan D, Bynner J, Richards D, Butler N, et al. Study of the aetiology of wheezing illness at age 16 in two national British birth cohorts. Thorax. 1996;51(7):670-6.

85. Lewis SA, Britton JR. Consistent effects of high socioeconomic status and low birth order, and the modifying effect of maternal smoking on the risk of allergic disease during childhood. Respir Med. 1998;92(10):1237-44.

86. Lima JA, Fischer GB, Sarria EE, Mattiello R, Solé D. Prevalence of and risk factors for wheezing in the first year of life. J Bras Pneumol. 2010;36(5):525-31.

87. Lin CH, Wang JL, Chen HH, Hsu JY, Chao WC. Shared prenatal impacts among childhood asthma, allergic rhinitis and atopic dermatitis: a population-based study. Allergy Asthma Clin Immunol. 2019;15:52.

88. Linneberg A, Simonsen JB, Petersen J, Stensballe LG, Benn CS. Differential effects of risk factors on infant wheeze and atopic dermatitis emphasize a different etiology. J Allergy Clin Immunol. 2006;117(1):184-9.

89. Loss GJ, Depner M, Hose AJ, Genuneit J, Karvonen AM, Hyvarinen A, et al. The early development of wheeze environmental determinants and genetic susceptibility at 17q21. American Journal of Respiratory and Critical Care Medicine. 2016;193(8):889-97.

90. Martins M, Pereira N, Reis R, Tomaz E. Risk factors for recurrent wheezing after the first hospitalization for bronchiolitis. Revista Portuguesa de Imunoalergologia. 2015;23(4):223-30.

91. McCandless RE. Birth order and age as factors in emotionally-triggered asthma. Aust J Physiother. 1970;16(4):151-4.

92. McKeever TM, Lewis SA, Smith C, Collins J, Heatlie H, Frischer M, et al. Siblings, multiple births, and the incidence of allergic disease: a birth cohort study using the West Midlands general practice research database. Thorax. 2001;56(10):758-62.

93. McLeod C. Prematurity, Socioeconomic Status, And Childhood Asthma: A Canadian Cohort Study. Electronic Thesis and Dissertation Repository. 2020.

94. Mejias SG, Ramphul K. Prevalence and Associated Risk Factors of Bronchial Asthma in Children in Santo Domingo, Dominican Republic. Cureus. 2018;10(2):e2211.

95. Midodzi WK. Early life determinants of asthma and wheezing: A longitudinal study of Canadian children. Ann Arbor: University of Alberta (Canada); 2007.

96. Miyake Y, Yura A, Iki M. Cross-sectional study of allergic disorders in relation to familial factors in Japanese adolescents. Acta Paediatr. 2004;93(3):380-5.

97. Miyake Y, Tanaka K, Arakawa M. Sibling number and prevalence of allergic disorders in pregnant Japanese women: baseline data from the Kyushu Okinawa Maternal and Child Health Study. BMC Public Health. 2011;11:561.

98. Mommers M, Weishoff-Houben M, Swaen GM, Creemers H, Freund H, Dott W, et al. Infant immunization and the occurrence of atopic disease in Dutch and German children: a nested case-control study. Pediatr Pulmonol. 2004;38(4):329-34.

99. Moncayo AL, Vaca M, Oviedo G, Erazo S, Quinzo I, Fiaccone RL, et al. Risk factors for atopic and non-atopic asthma in a rural area of Ecuador. Thorax. 2010;65(5):409-16.

100. Moraes LS, Takano OA, Mallol J, Solé D. Risk factors associated with wheezing in infants. J Pediatr (Rio J). 2013;89(6):559-66.

101. Morata-Alba J, Romero-Rubio MT, Castillo-Corullón S, Escribano-Montaner A. Respiratory morbidity, atopy and asthma at school age in preterm infants aged 32-35 weeks. Eur J Pediatr. 2019;178(7):973-82.

102. Nafstad P, Magnus P, Jaakkola JJ. Early respiratory infections and childhood asthma. Pediatrics. 2000;106(3):E38.

103. Nafstad P, Brunekreef B, Skrondal A, Nystad W. Early respiratory infections, asthma, and allergy: 10-year follow-up of the Oslo Birth Cohort. Pediatrics. 2005;116(2):e255-62.

104. Nicolai A, Frassanito A, Nenna R, Cangiano G, Petrarca L, Papoff P, et al. Risk Factors for Virus-induced Acute Respiratory Tract Infections in Children Younger Than 3 Years and Recurrent Wheezing at 36 Months Follow-Up After Discharge. Pediatr Infect Dis J. 2017;36(2):179-83.

105. Nicolaou NC, Simpson A, Lowe LA, Murray CS, Woodcock A, Custovic A. Day-care attendance, position in sibship, and early childhood wheezing: a population-based birth cohort study. J Allergy Clin Immunol. 2008;122(3):500-6.e5.

106. Nowak D, Heinrich J, Jorres R, Wassmer G, Berger J, Beck E, et al. Prevalence of respiratory symptoms, bronchial hyperresponsiveness and atopy among adults: West and East Germany. European Respiratory Journal. 1996;9(12):2541-52.

107. Ohfuji S, Miyake Y, Arakawa M, Tanaka K, Sasaki S. Sibship size and prevalence of allergic disorders in Japan: the Ryukyus Child Health Study. Pediatr Allergy Immunol. 2009;20(4):377-84.

108. Oliveira-Santos S, Motta-Franco J, Barreto I, Solé D, Gurgel R. Asthma in adolescents--Prevalence trends and associated factors in northeast Brazil. Allergol Immunopathol (Madr). 2015;43(5):429-35.

109. Oluwole O, Arinola OG, Falade GA, Ige MA, Falusi GA, Aderemi T, et al. Allergy sensitization and asthma among 13-14 year old school children in Nigeria. African Health Sciences. 2013;13(1):144-53.

110. Ozmert EN, Kale-Cekinmez E, Yurdakök K, Sekerel BE. Determinants of allergic signs and symptoms in 24- 48-month-old Turkish children. Turk J Pediatr. 2009;51(2):103-9.

111. Parthasarathi A, Padukudru S, Rajgopal N, Holla AD, Krishna MT, Mahesh PA. Allergic disease prevalence in school children in Bengaluru, India: A cross-sectional survey. Clinical and Experimental Allergy. 2021;51(7):955-8.

112. Pekkanen J, Remes S, Kajosaari M, Husman T, Soininen L. Infections in early childhood and risk of atopic disease. Acta Paediatr. 1999;88(7):710-4.

113. Pekkanen J, Xu B, Järvelin MR. Gestational age and occurrence of atopy at age 31--a prospective birth cohort study in Finland. Clin Exp Allergy. 2001;31(1):95-102.

114. Pérez Tarazona S, Alfonso Diego J, Amat Madramany A, Chofre Escrihuela L, Lucas Sáez E, Bou Monterde R. Incidence of wheezing and associated risk factors in the first 6 months of life of a cohort in Valencia (Spain). An Pediatr (Barc). 2010;72(1):19-29.

115. Perzanowski M, Canfield S, Chew G, Mellins R, Hoepner L, Jacobson J, et al. Birth order, atopy, and symptoms of asthma among low-income inner-city children attending preschool in New York City. Allergy. 2008;63:307-.

116. Ponsonby A-L, Couper D, Dwyer T, Carmichael A. Cross sectional study of the relation between sibling number and asthma, hay fever, and eczema. Archives of Disease in Childhood. 1998;79(4):328-33.

117. Ponsonby A-L, Couper D, Dwyer T, Carmichael A, Kemp A. Relationship between early life respiratory illness, family size over time, and the development of asthma and hay fever: a seven year follow up study. Thorax. 1999;54(8):664-9.

118. Ponsonby AL, Dwyer T, Kemp A, Lim L, Cochrane J, Carmichael A. The use of mutually exclusive categories for atopic sensitization: a contrasting effect for family size on house dust mite sensitization compared with ryegrass sensitization. Pediatr Allergy Immunol. 2003;14(2):81-90.

119. Räsänen M, Laitinen T, Kaprio J, Koskenvuo M, Laitinen LA. Hay fever, asthma and number of older siblings--a twin study. Clin Exp Allergy. 1997;27(5):515-8.

120. Räsänen M, Kaprio J, Laitinen T, Winter T, Koskenvuo M, Laitinen LA. Perinatal risk factors for asthma in Finnish adolescent twins. Thorax. 2000;55(1):25-31.

121. Rehalia N, Sharma J, Chaudhary S. Clinical profile and environmental risk factors of asthma in children at a tertiary care teaching hospital in the sub-Himalayan belt of Northern India. Indian Journal of Allergy, Asthma and Immunology. 2020;34(2):74.

122. Rona RJ, Duran-Tauleria E, Chinn S. Family size, atopic disorders in parents, asthma in children, and ethnicity. J Allergy Clin Immunol. 1997;99(4):454-60.

123. Rona RJ, Hughes JM, Chinn S. Association between asthma and family size between 1977 and 1994. J Epidemiol Community Health. 1999;53(1):15-9.

124. Rönmark E, Bjerg A, Perzanowski M, Platts-Mills T, Lundbäck B. Major increase in allergic sensitization in schoolchildren from 1996 to 2006 in northern Sweden. J Allergy Clin Immunol. 2009;124(2):357-63, 63.e1-15.

125. Ronmark EP, Ekerljung L, Mincheva R, Sjolander S, Hagstad S, Wennergren G, et al. Different risk factor patterns for adult asthma, rhinitis and eczema: Results from West Sweden Asthma Study. Clinical and Translational Allergy. 2016;6(1):28.

126. Rusconi F, Galassi C, Corbo GM, Forastiere F, Biggeri A, Ciccone G, et al. Risk factors for early, persistent, and late-onset wheezing in young children. SIDRIA Collaborative Group. Am J Respir Crit Care Med. 1999;160(5):1617-22.

127. Rusconi F, Galassi C, Bellasio M, Piffer S, Lombardi E, Bonci E, et al. Risk factors in the pre-, perinatal and early life (first year) for wheezing in young children. Epidemiol Prev. 2005;29(2):47-51.

128. Rutter CE, Silverwood RJ, Asher MI, Ellwood P, Pearce N, Garcia-Marcos L, et al. Comparison of individual-level and population-level risk factors for rhinoconjunctivitis, asthma, and eczema in the International Study of Asthma and Allergies in Childhood (ISAAC) Phase Three. World Allergy Organization Journal. 2020;13(6).

129. Salam MT, Li YF, Langholz B, Gilliland FD. Early-life environmental risk factors for asthma: findings from the Children's Health Study. Environ Health Perspect. 2004;112(6):760-5.

130. Ganesh Kumar S, Premarajan KC, Sarkar S, Sahu SK, Sahana, Ambika, et al. Prevalence and factors associated with asthma among school children in rural Puducherry, India. Current Pediatric Research. 2012;16(2):159-63.

131. Kumar G, Roy G, Subitha L, Sahu S. Prevalence of bronchial asthma and its associated factors among school children in urban Puducherry, India. Journal of Natural Science, Biology and Medicine. 2014;5(1):59-62.

132. Schmitz R, Atzpodien K, Schlaud M. Prevalence and risk factors of atopic diseases in German children and adolescents. Pediatr Allergy Immunol. 2012;23(8):716-23.

133. Sears MR, Holdaway MD, Flannery EM, Herbison GP, Silva PA. Parental and neonatal risk factors for atopy, airway hyper-responsiveness, and asthma. Arch Dis Child. 1996;75(5):392-8.

134. Sheldrake P, Cormack M, McGuire J. Psychosomatic illness, birth order and intellectual preference - I. Men. J Psychosom Res. 1976;20(1):37-44.

135. Sheldrake P, Cormack M, McGuire J. Psychosomatic illness, birth order and intellectual preference - II. Women. J Psychosom Res. 1976;20(1):45-9.

136. Sherriff A, Peters TJ, Henderson J, Strachan D. Risk factor associations with wheezing patterns in children followed longitudinally from birth to 3(1/2) years. Int J Epidemiol. 2001;30(6):1473-84.

137. Shohat T, Green MS, Davidson Y, Livne I, Tamir R, Garty BZ. Differences in the prevalence of asthma and current wheeze between Jews and Arabs: results from a national survey of schoolchildren in Israel. Ann Allergy Asthma Immunol. 2002;89(4):386-92.

138. Simões M, Inoue Y, Matsunaga NY, Carvalho MRV, Ribeiro GLT, Morais EO, et al. Recurrent wheezing in preterm infants: Prevalence and risk factors. J Pediatr (Rio J). 2019;95(6):720-7.

139. Slob EMA, Brew BK, Vijverberg SJH, Kats CJAR, Longo C, Pijnenburg MW, et al. Early-life antibiotic use and risk of asthma and eczema: results of a discordant twin study. European Respiratory Journal. 2020;55(4).

140. Smith JM. Asthma in School Children: University of Glasgow; 1960.

141. Strachan DP, Aït-Khaled N, Foliaki S, Mallol J, Odhiambo J, Pearce N, et al. Siblings, asthma, rhinoconjunctivitis and eczema: a worldwide perspective from the International Study of Asthma and Allergies in Childhood. Clin Exp Allergy. 2015;45(1):126-36.

142. Stråvik M, Barman M, Hesselmar B, Sandin A, Wold AE, Sandberg A-S. Maternal intake of cow’s milk during lactation is associated with lower prevalence of food allergy in offspring. Nutrients. 2020;12(12):3680.

143. Sugiura S, Hiramitsu Y, Futamura M, Kamioka N, Yamaguchi C, Umemura H, et al. Prevalence and associated factors of wheeze in early infancy. Pediatr Int. 2021;63(7):818-24.

144. Sunyer J, Anto JM, Kogevinas M, Barcelo MA, Soriano JB, Tobias A, et al. Risk factors for asthma in young adults. European Respiratory Journal. 1997;10(11):2490-4.

145. Svanes C, Jarvis D, Chinn S, Omenaas E, Gulsvik A, Burney P. Early exposure to children in family and day care as related to adult asthma and hay fever: results from the European Community Respiratory Health Survey. Thorax. 2002;57(11):945-50.

146. Teijeiro A, Cuello MN, Raiden MG, Vieyra RE. Risk factors and protective factors for recurrent wheezing in the first year of life in the city of Cordoba, Argentina. American Journal of Respiratory and Critical Care Medicine. 2019;199(9).

147. Toppila-Salmi S, Chanoine S, Karjalainen J, Pekkanen J, Bousquet J, Siroux V. Risk of adult-onset asthma increases with the number of allergic multimorbidities and decreases with age. Allergy. 2019;74(12):2406-16.

148. Toppila-Salmi S, Lemmetyinen R, Chanoine S, Karjalainen J, Pekkanen J, Bousquet J, et al. Risk factors for severe adult-onset asthma: a multi-factor approach. BMC Pulm Med. 2021;21(1):214.

149. van Beijsterveldt T, Boomsma DI. An exploration of gene-environment interaction and asthma in a large sample of 5-year-old Dutch twins. Twin Research and Human Genetics. 2008;11(2):143-9.

150. Venero-Fernández SJ, Suárez-Medina R, Mora-Faife EC, García-García G, Valle-Infante I, Gómez-Marrero L, et al. Risk factors for wheezing in infants born in Cuba. Qjm. 2013;106(11):1023-9.

151. Venter C, Palumbo MP, Sauder KA, Glueck DH, Liu AH, Yang IV, et al. Incidence and timing of offspring asthma, wheeze, allergic rhinitis, atopic dermatitis, and food allergy and association with maternal history of asthma and allergic rhinitis. World Allergy Organization Journal. 2021;14(3):100526.

152. Victorino CC, Gauthier AH. The social determinants of child health: variations across health outcomes - a population-based cross-sectional analysis. BMC Pediatr. 2009;9:53.

153. Westergaard T, Rostgaard K, Wohlfahrt J, Andersen PK, Aaby P, Melbye M. Sibship characteristics and risk of allergic rhinitis and asthma. Am J Epidemiol. 2005;162(2):125-32.

154. Wickens KL, Crane J, Kemp TJ, Lewis SJ, D'Souza WJ, Sawyer GM, et al. Family size, infections, and asthma prevalence in New Zealand children. Epidemiology. 1999;10(6):699-705.

155. Wolff PT, Arison L, Rahajamiakatra A, Raserijaona F, Niggemann B. High asthma prevalence and associated factors in urban malagasy schoolchildren. J Asthma. 2012;49(6):575-80.

156. Yuan W, Fonager K, Olsen J, Sørensen HT. Prenatal factors and use of anti-asthma medications in early childhood: a population-based Danish birth cohort study. Eur J Epidemiol. 2003;18(8):763-8.

157. Zadnick JM. The effect of family structure on risk of familial and sporadic self-reported asthma in a cohort of California twin pairs: University of Southern California; 2010.

158. Zekveld C, Bibakis I, Bibaki-Liakou V, Pedioti A, Dimitroulis I, Harris J, et al. The effects of farming and birth order on asthma and allergies. Eur Respir J. 2006;28(1):82-8.
